# Supplementary material for: C-JUN overexpressing CAR-T cells in acute myeloid leukemia: preclinical characterization and phase I trial
Source: Nat Commun. 2024 Jul 22;15:6155. doi: 10.1038/s41467-024-50485-9 (PMC11263573; doi:10.1038/s41467-024-50485-9)
Supplement: Supplementary file 1 — Supplementary Information [file 41467_2024_50485_MOESM1_ESM.pdf]

Supplementary information for

C-JUN overexpressing CAR-T cells in acute myeloid leukemia: preclinical characterization and phase Ⅰ trial

Authors:

Shiyu Zuo[1,2†], Chuo Li[1,2,3†], Xiaolei Sun[1,2,†], Biping Deng[4], Yibing Zhang[1,2], Yajing Han[1,2], Zhuojun Ling[5], Jinlong Xu[5], Jiajia Duan[5], Zelin Wang[5], Xinjian Yu[6], Qinlong Zheng[6], Xiuwen Xu[6], Jiao Zong[6], Zhenglong Tian[7], Lingling Shan[1,2], Kaiting Tang[1,2], Huifang Huang[3], Yanzhi Song[8], Qing Niu[1,2], Dongming Zhou[9], Sizhou Feng[1,2], Zhongchao Han[10], Guoling Wang[11,1,2*], Tong Wu[8*], Jing Pan[12*], Xiaoming Feng[1,2,3*]

The PDF file includes:

Supplementary Figures (Fig. S#)

Fig. S1: Killing efficiency and phenotype of CAR T cells in myeloid and lymphoid leukemia cells.

Fig. S2: JNK_MAPK and NFAT pathway and total and phosphorylated ERK, C-JUN, and ZAP70 protein levels in CAR T cells.

Fig. S3: The expression of ROS and NO in tumor cells and the effect of blocking ROS and NO on CAR T cell-mediated cytotoxicity *in vitro*.

Fig. S4: The effect of CD155 on CAR T cells and the knockout efficiency of CD155-associated receptors on CAR T cells *in vitro*.

Fig. S5: The effect of overexpression of specific factors on the anti-tumor function of CAR T cells *in vivo*.

Fig. S6: C-JUN overexpression enhances the anti-tumor activity of CD33 CAR T cells.

Fig. S7: RNAseq analysis of U937-exposed CD33 CAR T cells with or without C-JUN overexpression and total and phosphorylated ERK, C-JUN, and JNK protein levels in CAR T cells..

Fig. S8: Effects of ERK inhibitor (U0126) on expression of costimulatory molecules and cytokines in control and C-JUN CAR T cells.

Fig. S9: Patient 3 relapsed with CD33 positive blasts one year after receiving C-JUN CAR T-cell infusion.

Fig. S10: Gating strategies for flow cytometry analysis.

Fig. S11: Gating strategies for flow cytometry analysis.

Supplementary Tables (Table. S#)

Table S1: Prior lines of therapy.

Table S2: Characteristics of CAR T cell products.

Table S3: Adverse events and grading post-CAR T cell infusion for each patient.

Table S4: Information of antibodies and other reagents.

Table S5: Characteristics of primary samples.

Table S6: List of gene set related to T-cell exhaustion features.

Supplementary Note

Clinical trial protocol

Statistical analysis plan

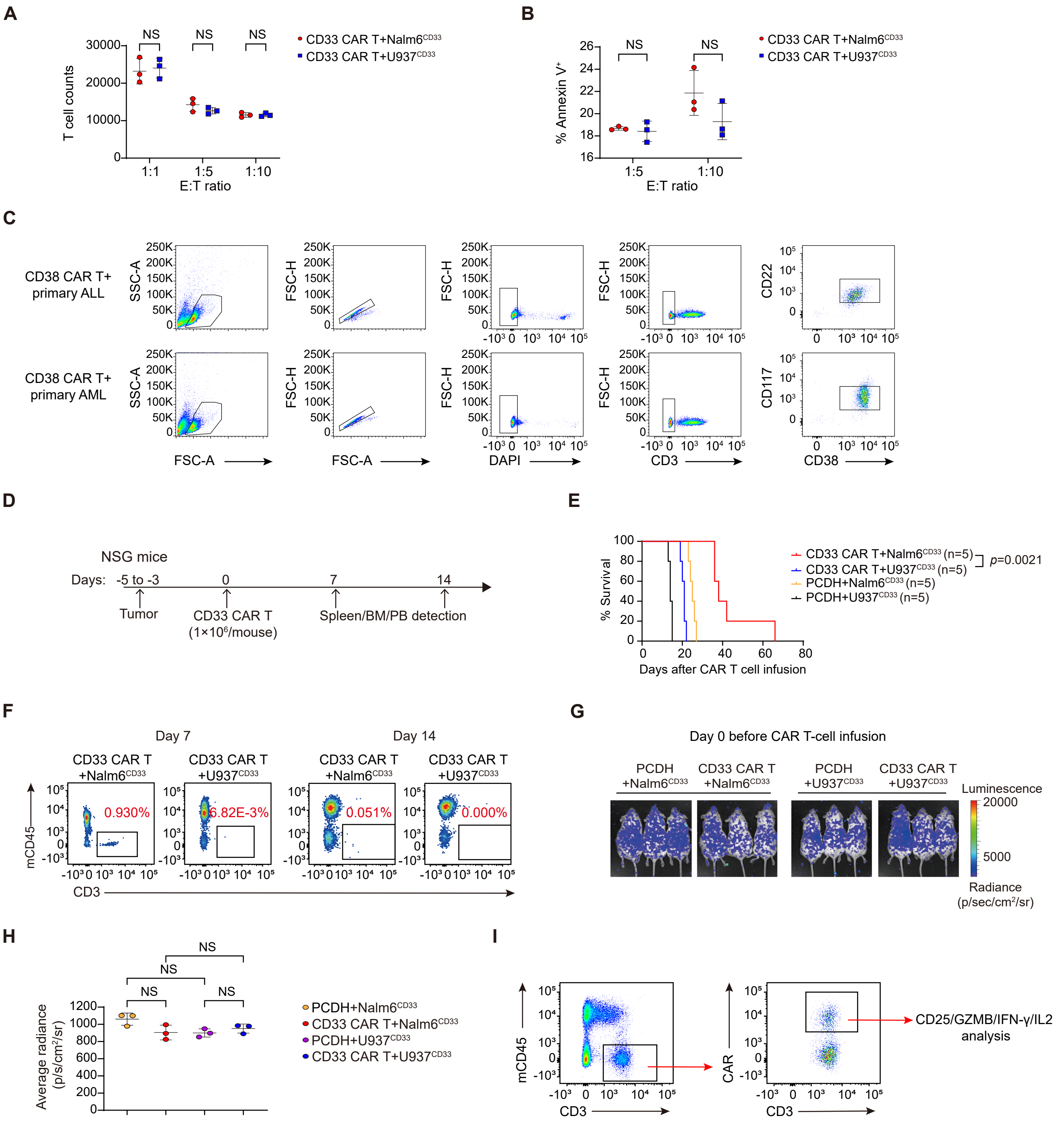

Supplementary Figure 1. Killing efficiency and phenotype of CAR T cells in myeloid and lymphoid leukemia cells. **(A)** T cell counts of CD33 CAR T cells co-incubated with $U937^{CD33}$ or $Nalm6^{CD33}$ cells at the indicated effector:target (E:T) ratios, n = 3. **(B)** Percentage of Annexin V^+ cells in T cells from **A** at 1:5 and 1:10 E:T ratios, n = 3. **(C)** Gating strategy of assessing the cytotoxicity of CD38 CAR T cells against primary $CD38^+$ AML and ALL samples. **(D)** Schematic of the mouse model. Tumor cells were intravenously injected in NSG mice followed by intravenous infusion of 1×10^6 CAR or PCDH T cells three or five days later. Mice were euthanized on days 7 to 14, and cells were obtained for analysis from peripheral blood (PB), spleen (SP), and bone marrow (BM). **(E)** Representative Kaplan-Meier curves summarizing from two experiments, n = 5. **(F)** Representative flow cytometric plots of PB from **D** on day 7 and day 14 post-infusion. **(G)** Representative tumor bioluminescence on day 0, n = 3. **(H)** Quantification of **(G)** showing the tumor burden as indicated by average radiance (p/s/cm^2/sr), n = 3. **(I)** Gating strategy for the analysis of CD25, granzymeB (GZMB), IFN-γ, and IL2 in CAR T cells from bearing $U937^{CD33}$ and $Nalm6^{CD33}$-bearing mice. For all bar plots, data are shown as mean ± SD. Assays were performed on day 10 after T-cell initial activation. Two-sided unpaired *t*-test or multiple two-sided unpaired *t* test was used in **(A, B)**. Survival curves were compared in **E** using the log-rank Mantel-Cox test. One-way ANOVA was used in **(H)**. All numbers defined by "n" indicate the number of biological replicates with different human donors or mice. Data are representative of two independent experiments. NS: not significant. Source data are provided in the Source Data file.

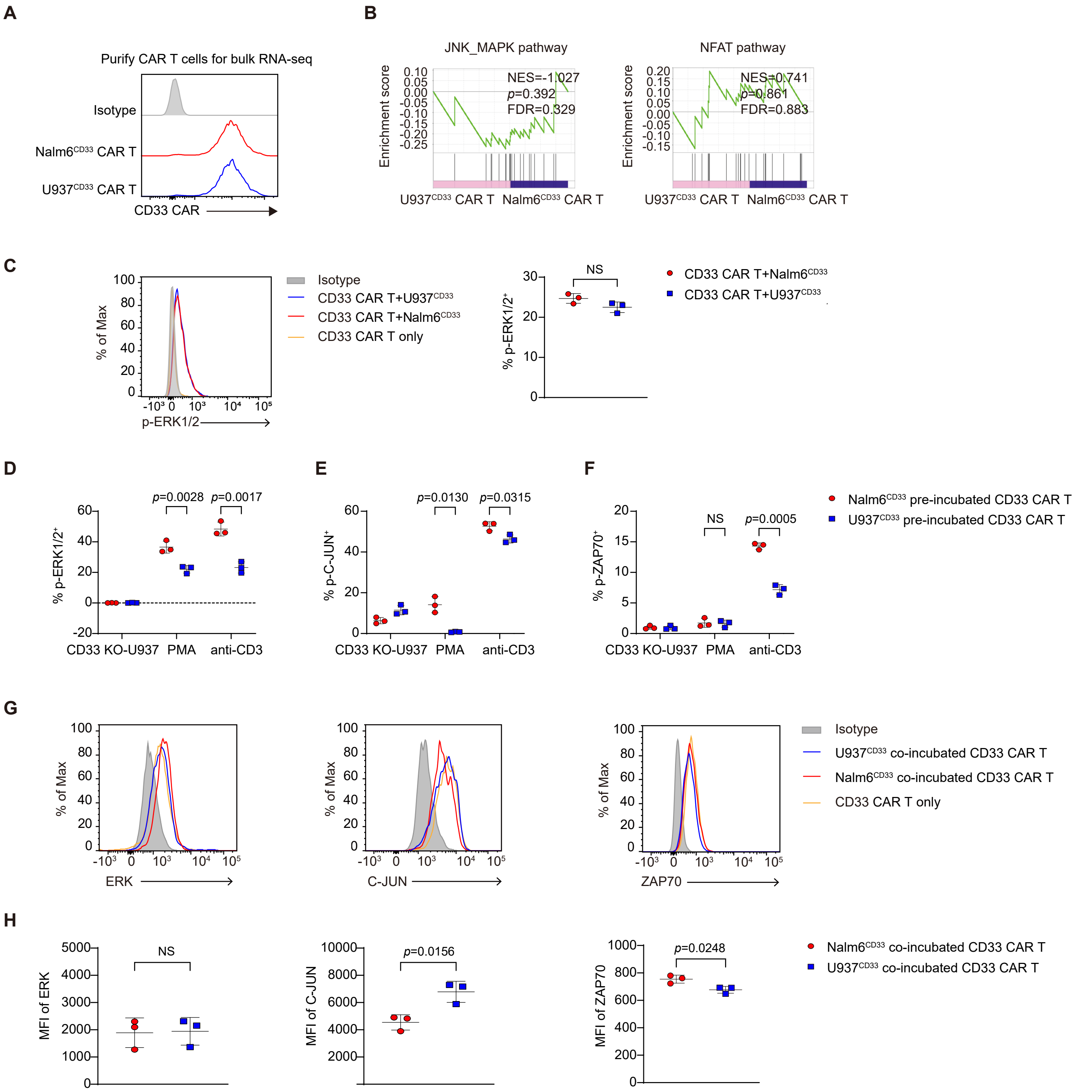

Supplementary Figure 2. JNK_MAPK and NFAT pathway and total and phosphorylated ERK, C-JUN, and ZAP70 protein levels in CAR T cells. (A) Representative histogram showing CAR expression on sorted and purified Nalm6^{CD33} co-incubated and U937^{CD33} co-incubated CD33 CAR T cells before RNA-seq. **(B)** GSEA of 'JNK_MAPK pathway' and 'NFAT pathway' with the RNA sequencing data of U937^{CD33} and Nalm6^{CD33} co-incubated CD33 CAR T cells. Nominal *P* values, FDR *q* values, and normalized enrichment score (NES) were calculated using GSEA software (Broad Institute). **(C)** The expression of phosphorylated ERK1/2 in CD33 CAR T cells. CD33 CAR T cells were not pre-incubated with Nalm6^{CD33} or U937^{CD33} cells, but co-incubated with Nalm6^{CD33} or U937^{CD33} cells for 15-30 minutes to stimulate phosphorylation events, n = 3. **(D-F)** The expression of phosphorylated ERK1/2 **(D)**, C-JUN **(E)**, and ZAP70 **(F)** in CD33 CAR T cells pre-incubated with U937^{CD33} and Nalm6^{CD33} cells, and restimulated with CD33-knockout (CD33-KO) U937 cells, PMA, and anti-CD3 antibody, n =3. **(G)** Representative histograms showing total ERK, ZAP70, and C-JUN protein expression in CAR T cells. The CAR T cells were co-incubated with Nalm6^{CD33} or U937^{CD33} cells at a 1:1 E:T ratio. **(H)** The mean fluorescence intensity (MFI) of total ERK, ZAP70, and C-JUN protein expression in CD33 CAR T cells. The CAR T cells were co-incubate with Nalm6^{CD33} or U937^{CD33} cells at a 1:1 E:T ratio, n = 3. For all bar plots, data are shown as mean ± SD. Assays were performed on day 10 after T-cell initial activation. Two-sided unpaired *t*-test or multiple two-sided unpaired *t* test was used in **(C, D-F, H)**. All numbers defined by "n" indicate the number of biological replicates with different human donors. Data are representative of two independent experiments. NS: not significant. Source data are provided in the Source Data file.

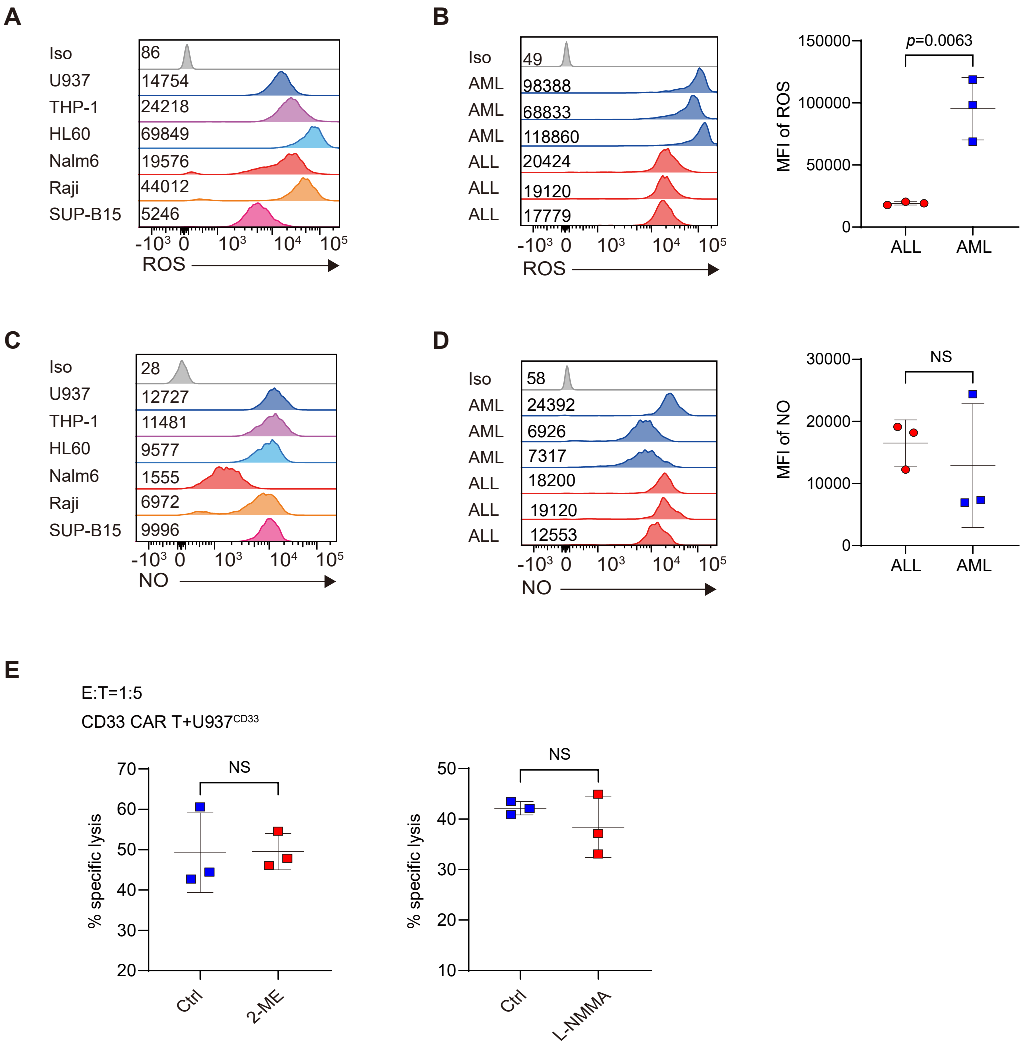

Supplementary Figure 3. The expression of ROS and NO in tumor cells and the effect of blocking ROS and NO on CAR T cell-mediated cytotoxicity *in vitro*. **(A)** Flow cytometric histograms showing ROS expression in AML and ALL cell lines. **(B)** Flow cytometric histograms and mean fluorescence intensity (MFI) of ROS expression in primary AML and ALL samples, n = 3. **(C)** Flow cytometric histograms showing NO expression in AML and ALL cell lines. **(D)** Flow cytometric histograms and MFI of NO expression in primary AML and ALL samples, n = 3. **(E)** CD33 CAR T-cell cytotoxicity against U937^{CD33} cells at 1:5 E:T ratio in the presence of 55 μM 2-ME (ROS inhibitor, left panel) or 10 μM L-NMMA (NO inhibitor, right panel), n = 3. For all bar plots, data are shown as mean ± SD. Assays were performed on day 10 after T-cell initial activation. Unpaired *t*-test was used in **(B, D, E)**. All numbers defined by "n" indicate the number of biological replicates with different human donors. Data are representative of two independent experiments. NS: not significant. Source data are provided in the Source Data file.

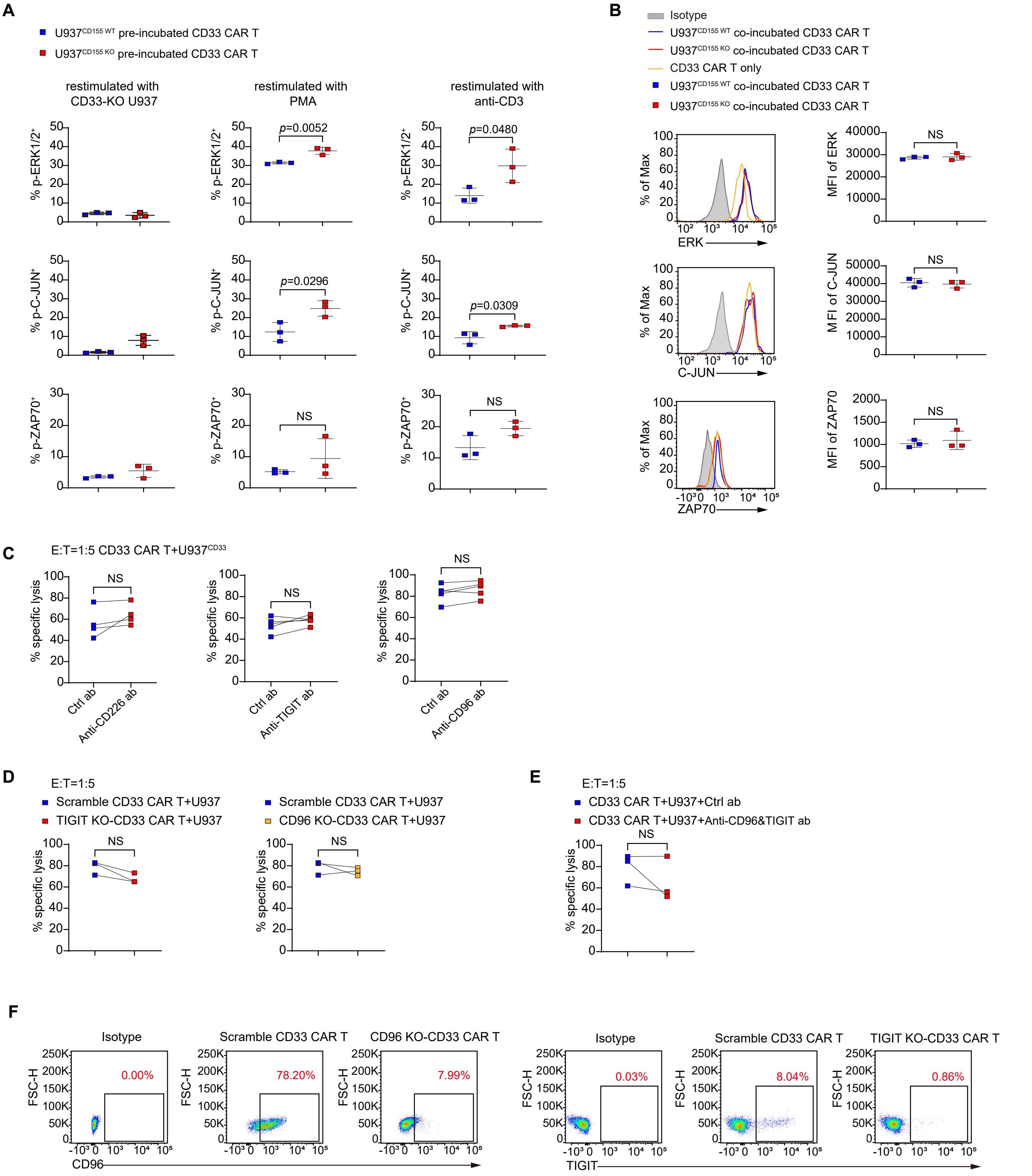

Supplementary Figure 4. The effect of CD155 on CAR T cells and the knockout efficiency of CD155-associated receptors on CAR T cells *in vitro*. (A) The expression of phosphorylated ERK1/2, phosphorylated C-JUN, and phosphorylated ZAP70 in CD33 CAR T cells pre-incubated with U937$^{CD155\ WT}$ and U937$^{CD155\ KO}$ cells, and restimulated with CD33-knockout (CD33-KO) U937 cells, PMA, and anti-CD3 antibody, n =3. **(B)** Representative histograms and the mean fluorescence intensity (MFI) of total ERK, ZAP70, and C-JUN protein expression in CAR T cells. The CAR T cells were co-incubated with U937$^{CD155\ WT}$ or U937$^{CD155\ KO}$ cells, n = 3. **(C)** Cytolytic activity of CD33 CAR T cells against U937 cells with 10 μg/ml CD226, 50 μg/ml TIGIT, or 20 μg/ml CD96 antibody, n = 4 in CD226 antibody group, n = 5 otherwise. **(D)** Cytolytic activity of scramble-, CD96 knockout (CD96 KO), and TIGIT knockout (TIGIT KO)-CD33 CAR T cells against U937 cells, n = 3. The scrambled CD33 CAR T were electroporated with non-targeting sgRNAs. **(E)** Cytolytic activity of CD33 CAR T cells against U937 cells with both 50 μg/ml TIGIT and 20 μg/ml CD96 antibody, n = 3. **(F)** Flow cytometric plots showing the CD96 and TIGIT knockout efficiency in CD33 CAR T cells. For all bar plots, data are shown as mean ± SD. Assays were performed on day 10 after T-cell initial activation. Unpaired *t*-test was used in **(A, B)**. Paired *t*-test was used in **(C, D, E)**. All numbers defined by "n" indicate the number of biological replicates with different human donors. Data are representative of two independent experiments. NS: not significant. Source data are provided in the Source Data file.

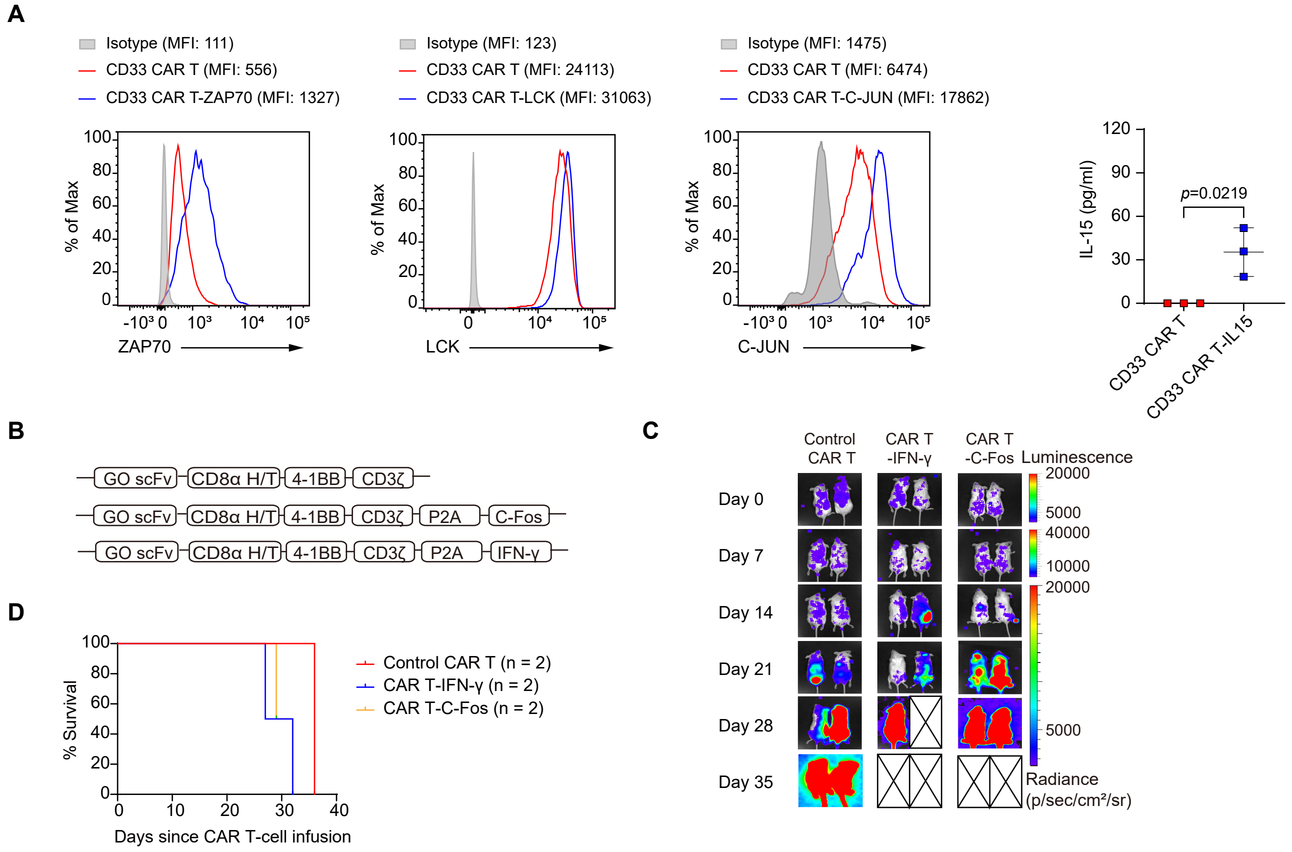

Supplementary Figure 5. The effect of overexpression of specific factors on the anti-tumor function of CAR T cells *in vivo*. **(A)** Representative flow cytometric histograms showing the expression of ZAP70, LCK, and C-JUN on CD33 CAR T cells. The expression of IL-15 was determined by ELISA. **(B)** Schematic illustration of CAR design, Gemtuzumab Ozogamicin (GO). **(C)** Representative tumor bioluminescence, using IVIS Lumina II *in vivo* imaging system, n = 2. **(D)** Kaplan-Meier curves showing the representative experiments, each line represents one treatment group, n = 2. Assays were performed on day 10 after T-cell initial activation. Unpaired *t*-test was used in **(A)**. All numbers defined by “n” indicate the number of biological replicates with different mice. Data are representative of two independent experiments. Source data are provided in the Source Data file.

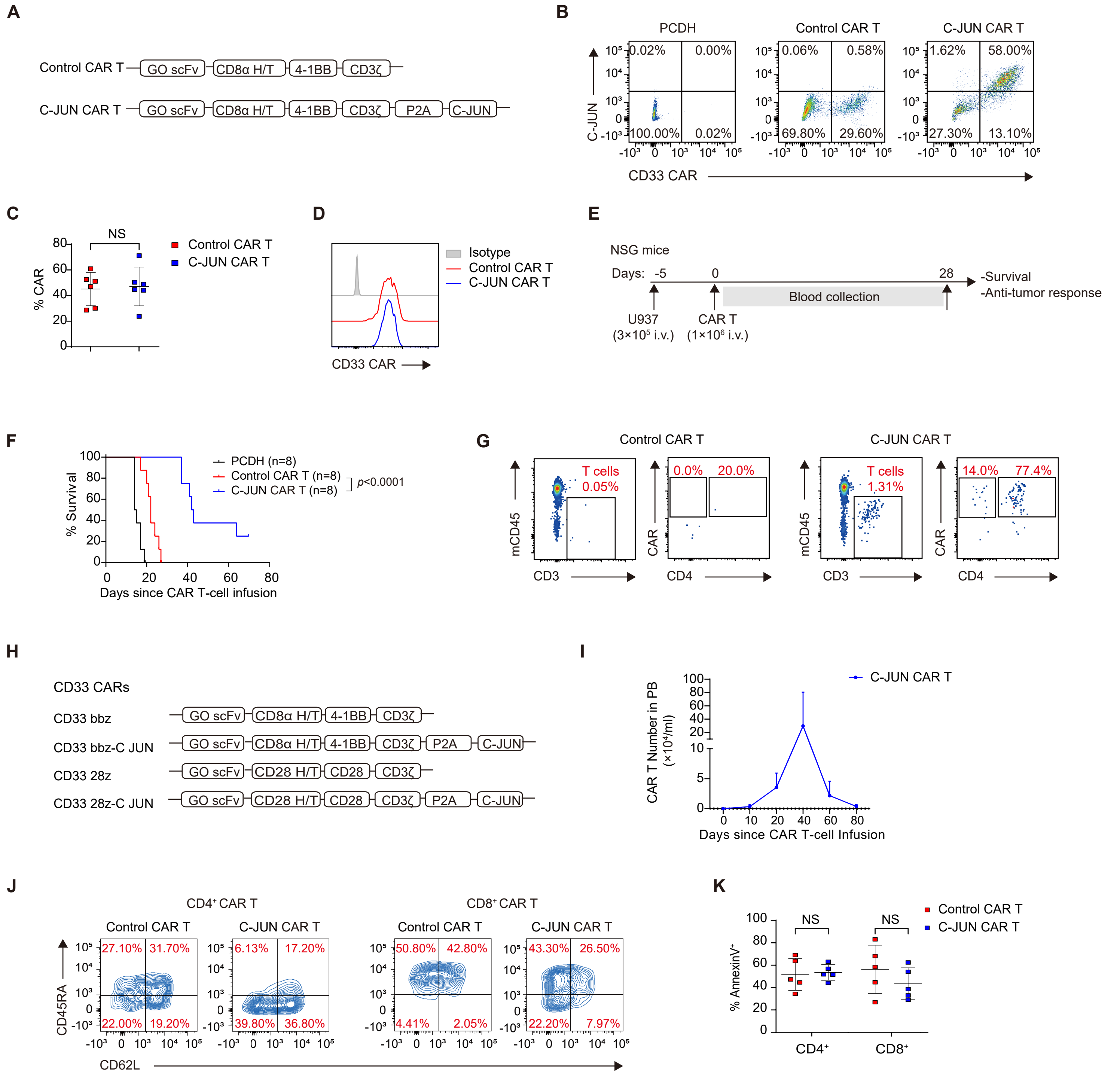

Supplementary Figure 6. C-JUN overexpression enhances the anti-tumor activity of CD33 CAR T cells. (A) Schematic illustration of CD33 CAR constructs, Gemtuzumab Ozogamicin (GO). **(B)** Representative flow cytometric plots showing CD33 CAR and C-JUN expression in T cells transduced with control CAR or C-JUN CAR. **(C)** The percentage of CAR transduction efficiency, summarized from multiple experiments, n = 6. **(D)** Representative histograms showing CAR⁺ expression in control CAR T and C-JUN CAR T cells which were sorted and purified. **(E)** Schematic of the mouse model. 3×10^5 U937 cells were intravenously injected into NSG mice followed by intravenous infusion of 1×10^6 control CAR T or C-JUN CAR T, or PCDH T cells five days later. **(F)** Kaplan-Meier curves summarizing three independent experiments, n = 8. Each line represents one treatment group. **(G)** Representative flow cytometric plots and gating strategy of peripheral blood (PB) from **E** on day 14 post-infusion. **(H)** Schematic illustration of different CD33 CARs design, Gemtuzumab Ozogamicin (GO). **(I)** CAR T-cell counts in PB, n = 3. **(J)** Representative flow cytometric plots of CD45RA and CD62L expression on control CAR T and C-JUN CAR T cells. **(K)** The percentage of Annexin V⁺ cells in control CAR T and C-JUN CAR T cells, n = 5. For all bar plots, data are shown as mean ± SD. Assays were performed on day 10 after T-cell initial activation. All numbers defined by "n" indicate the number of biological replicates with different human donors or mice. Two-sided unpaired *t*-test or multiple two-sided unpaired *t* test was used in **(C, K)**. Survival curves were compared using the log-rank Mantel-Cox test in **F**. Data are representative of two independent experiments. NS: not significant. Source data are provided in the Source Data file.

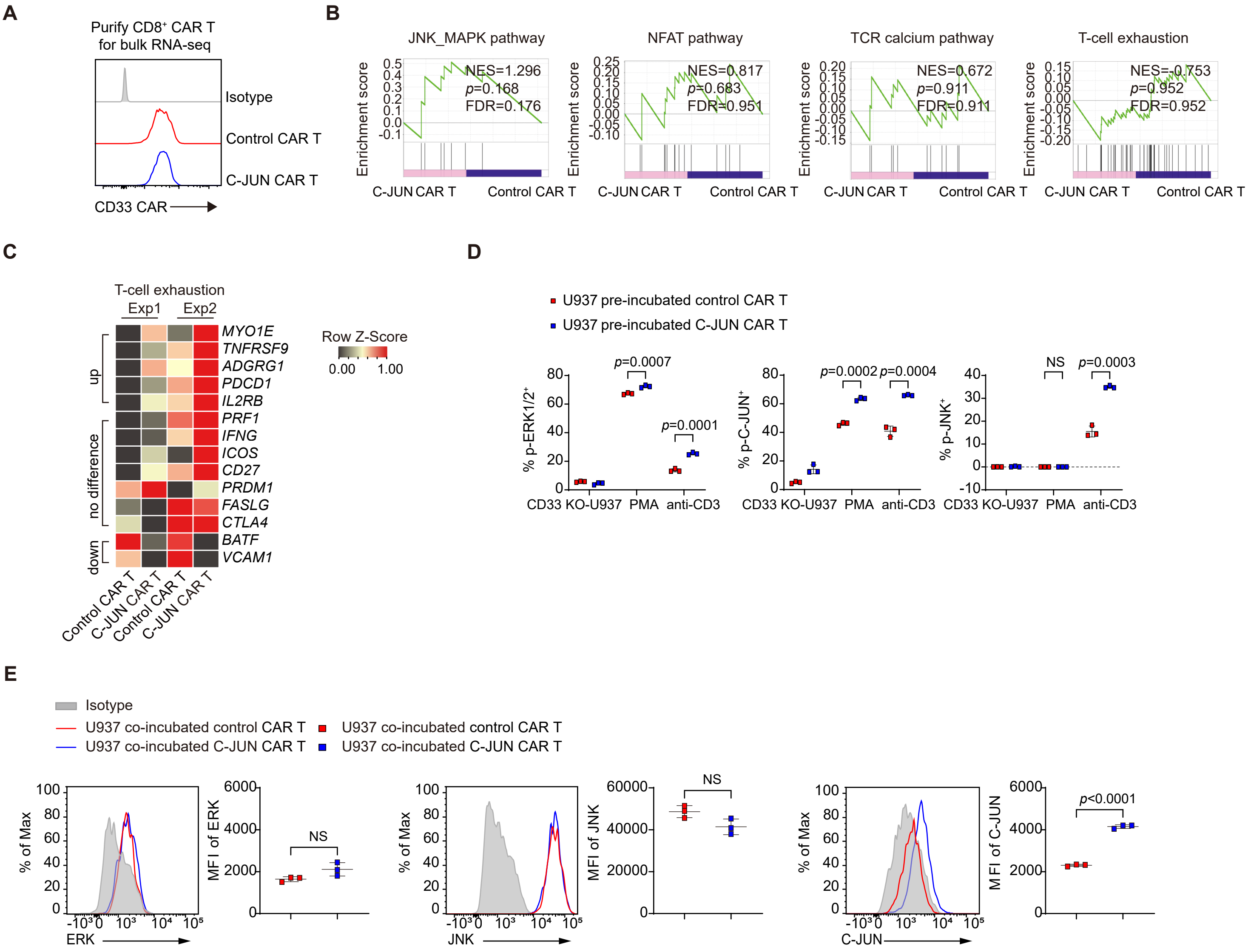

Supplementary Figure 7. RNAseq analysis of U937-exposed CD33 CAR T cells with or without C-JUN overexpression and total and phosphorylated ERK, C-JUN, and JNK protein levels in CAR T cells. (A) The histograms showing CAR expression on sorted and purified U937^{CD33} co-incubated control and C-JUN CAR T cells prior to RNA -seq. **(B)** GSEA of “JNK_MAPK pathway”, “NFAT pathway”, “TCR calcium pathway”, and “T-cell exhaustion” pathway with RNAseq data of control CAR T and C-JUN CAR T cells; nominal *P* values, FDR *q* values, and NES were calculated using GSEA software (Broad Institute). **(C)** Heat map showing differential expression of genes in the gene sets from **B**; some genes in exhaustion gene sets were of special interest and were also shown, despite that they were not differentially expressed. This experiment was performed twice with different donors, and each sample was pooled from three technical replicates. **(D)** The expression of phosphorylated ERK1/2, C-JUN, and JNK in control CAR T and C-JUN CAR T cells pre-incubated with U937 cells, and re-stimulated with CD33-knockout (CD33-KO) U937 cells, PMA, and anti-CD3 antibody, n =3. **(E)** Representative histograms and the mean fluorescence intensity (MFI) of total ERK, JNK, and C-JUN protein expression in CAR T cells. The CAR T cells were co-incubated U937 cells, n = 3. For all bar plots, data are shown as mean ± SD. Assays were performed on day 10 after T-cell initial activation. Two-sided unpaired *t*-test or multiple two-sided unpaired *t* test was used in **(D, E)**. All numbers defined by “n” indicate the number of biological replicates with different human donors. Data are representative of two independent experiments. NS: not significant. Source data are provided in the Source Data file.

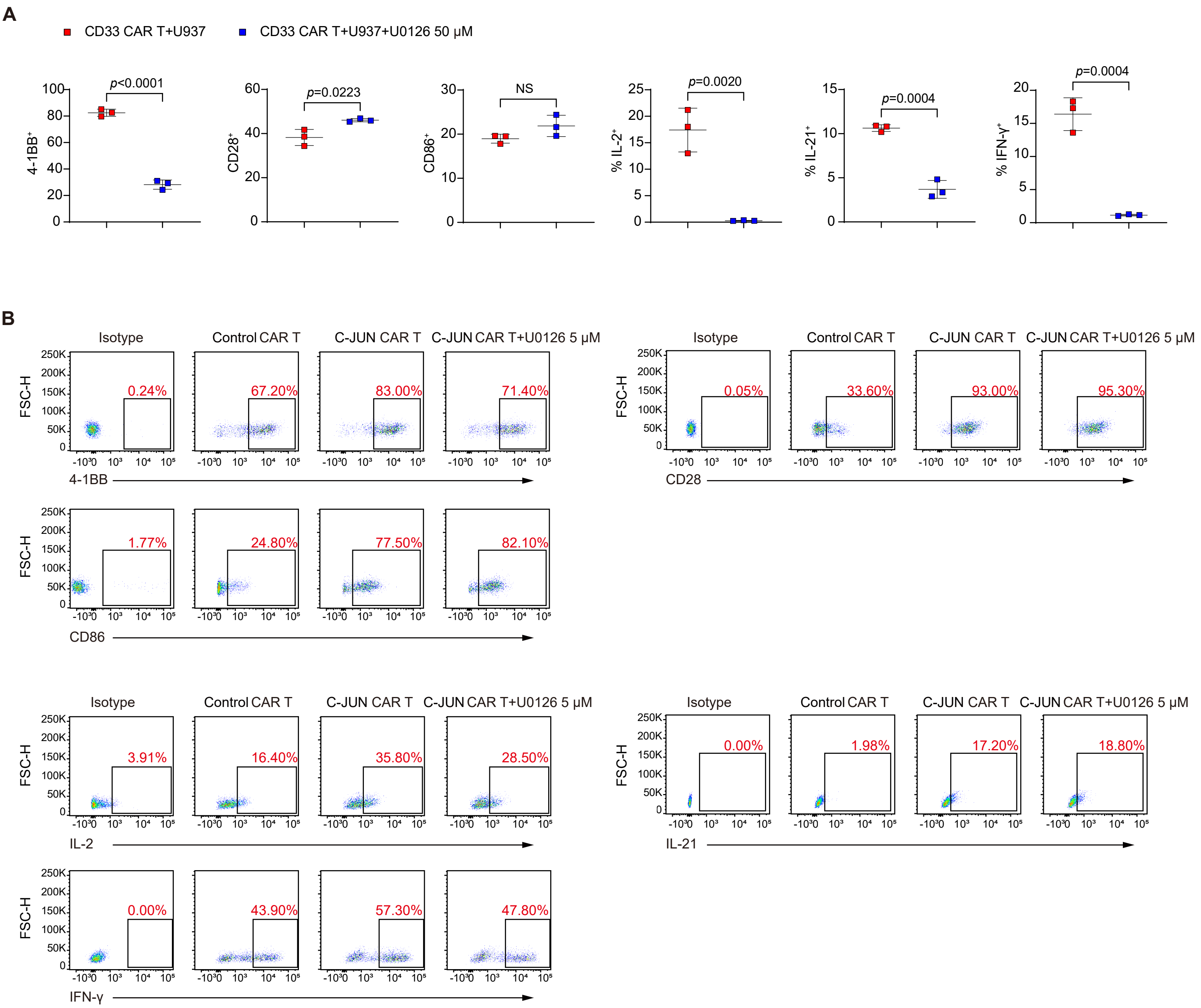

Supplementary Figure 8. Effects of ERK inhibitor (U0126) on expression of costimulatory molecules and cytokines in control and C-JUN CAR T cells. **(A)** The percentage of 4-1BB, CD28, CD86, IL-2, IL-21, and IFN-γ in control CD33 CAR T cells against U937 with or without 50 μM U0126 , n = 3. **(B)** Representative flow cytometric plots showing the percentage of 4-1BB, CD28, CD86, IL-2, IL-21, and IFN-γ positive cells in control and C-JUN CAR T cells cocultured with U937 cells in the presence of 5 μM U0126 (ERK inhibitor) .For all bar plots, data are shown as mean ± SD. Assays were performed on day 10 after T-cell initial activation. Unpaired *t*-test was used in **(A)**. All numbers defined by "n" indicate the number of biological replicates with different human donors. Data are representative of two independent experiments.

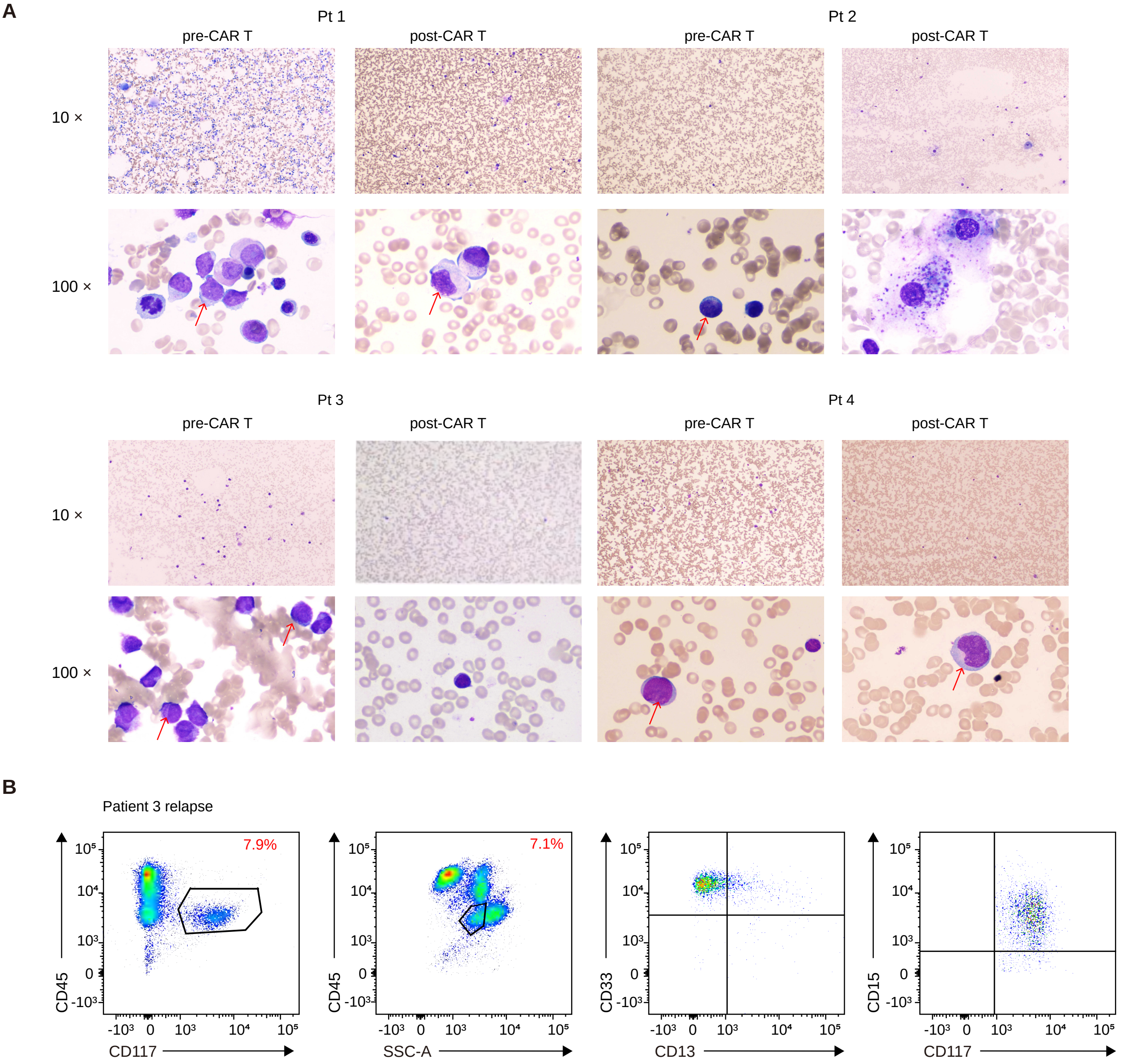

Supplementary Figure 9. Patient 3 relapsed with CD33 positive blasts one year after receiving C-JUN CAR T-cell infusion. **(A) T**he photomicrographs showing patient marrows at pre- and post CAR T cell infusion. Red arrows represent tumor cells that using Wright-Giemsa staining. Magnification, 10 × and 100 ×. **(B)** Representative flow cytometric plots showing the percentage of $CD33^{+}$ blasts in bone marrow (BM) samples in patient 3 who had a relapse one year after receiving C-JUN CAR T-cell infusion, as determined by flow cytometry.

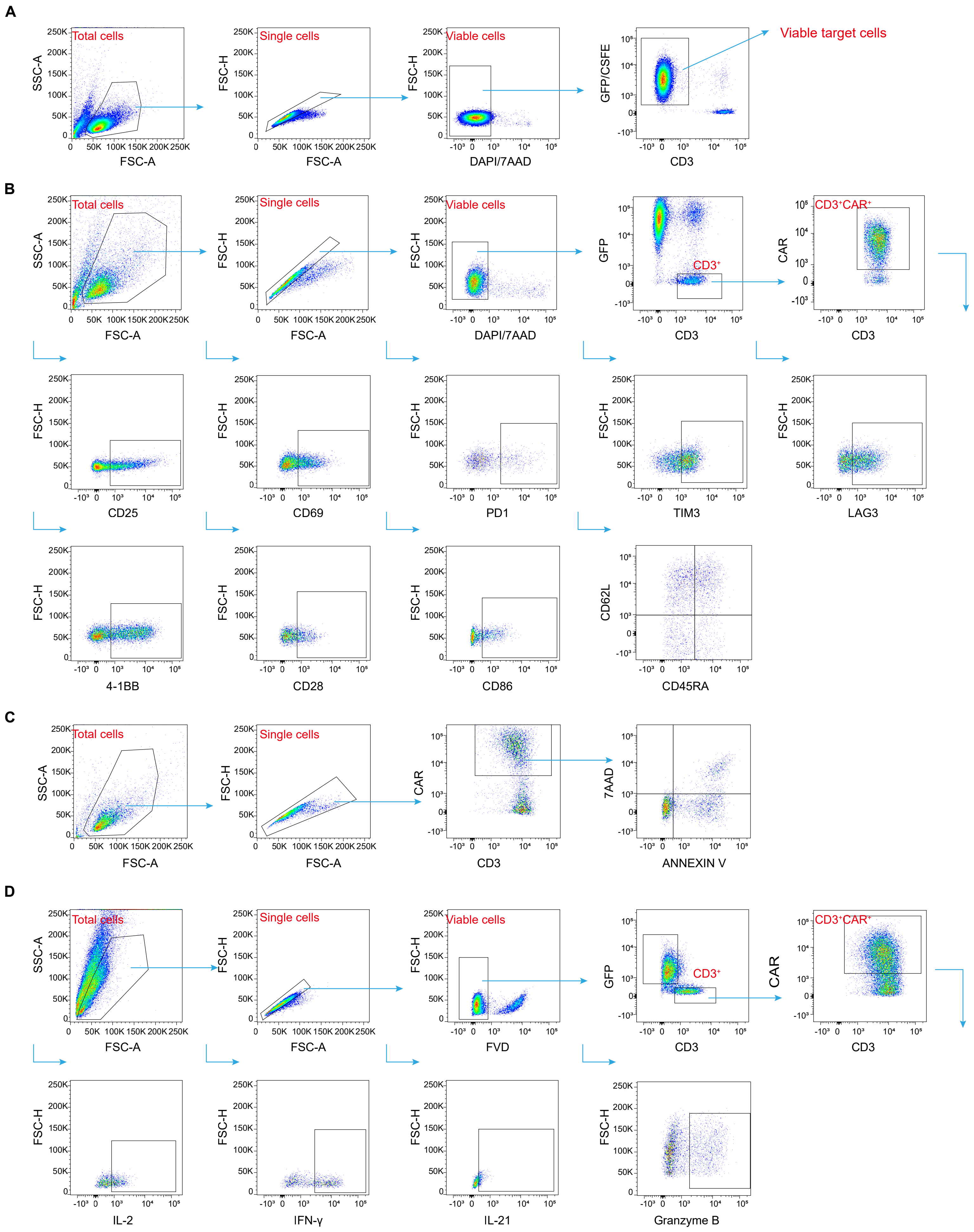

Supplementary Figure 10. Gating strategies for flow cytometry analysis. (A) Gating strategy for defining killing efficacy in Fig. 1B, 1C, 1E, 1F, 3C, 3E, 3G-L, 4R, 5G, 6J. **(B)** Gating strategy was used to analyze activation markers (CD25, CD69), exhaustion markers (PD1, TIM3, LAG3), co-stimulatory molecules (4-1BB, CD28, CD86), and memory (CD45RA and CD62L) phenotype of CAR T cells in Fig, 1D, 5J, 6I, 6K, 6N. **(C)** Gating strategy was used to analyze apoptosis (Annexin V and 7-AAD) of CAR T cells in Fig. 5I. **(D)** Gating strategy was used to analyze intracellular cytokine expression (IL-2, IFN-γ, IL-21, and Granzyme B (GZMB) of CAR T cells in Fig. 1D, 5H, 6L, 6O.

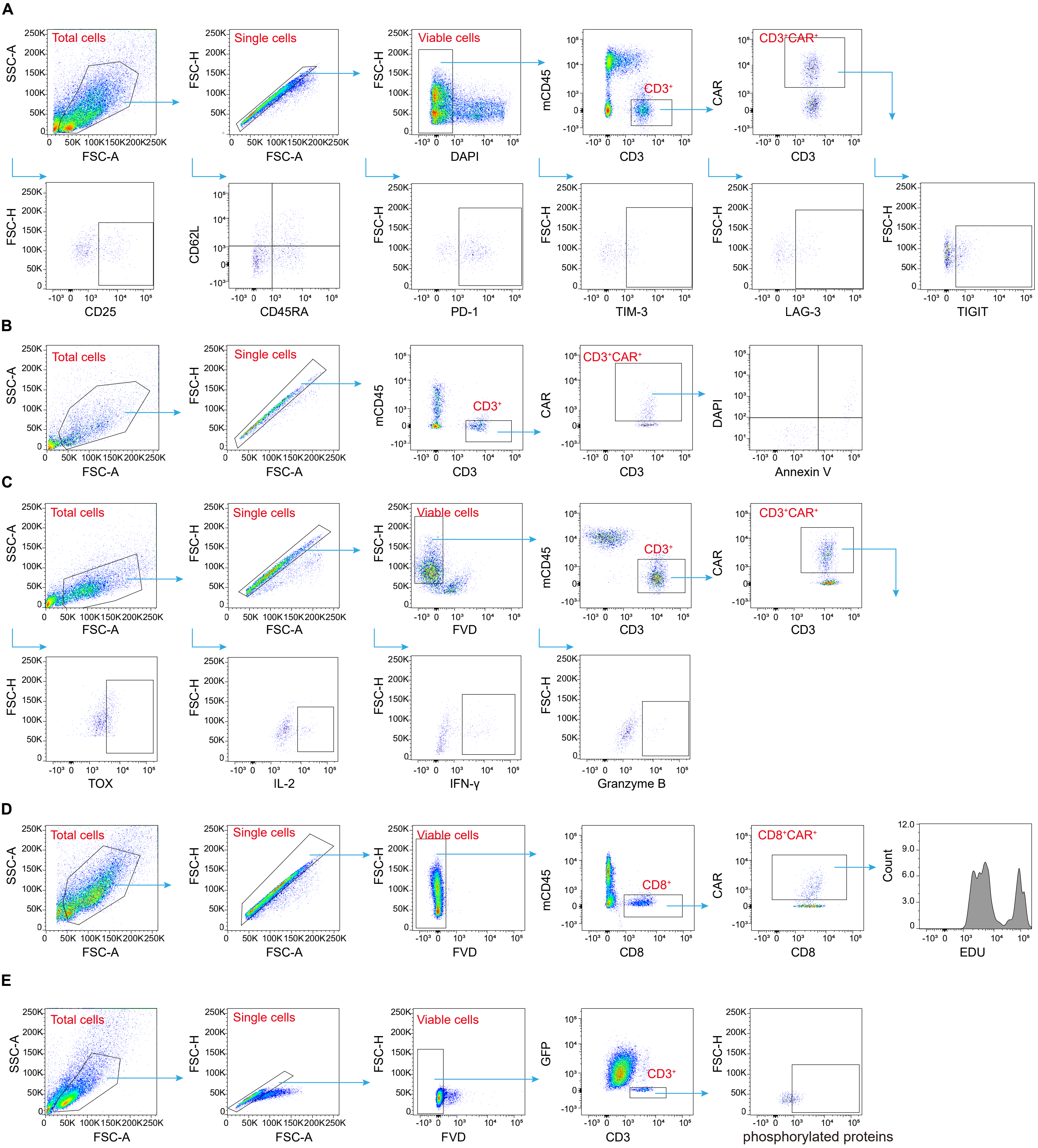

Supplementary Figure 11. Gating strategies for flow cytometry analysis. (A) Gating strategy was used to analyze activation marker (CD25), memory (CD45RA and CD62L), and exhaustion markers (PD1, TIM3, LAG3, TIGIT) phenotype of CAR T cells in mouse model in Fig, 1J, 1K, 1L, 5C, 5E, 5F. **(B)** Gating strategy was used to analyze apoptosis (Annexin V and DAPI) of CAR T cells in mouse model in Fig. 1I. **(C)** Gating strategy was used to analyze TOX and intracellular cytokine expression (IL-2, IFN-γ, and Granzyme B (GZMB) of CAR T cells in mouse model in Fig. 1J, 5D, 5F. **(D)** Gating strategy was used to analyze EDU of CD8^{+} CAR T cells in mouse model in Fig. 5B. **(E)** Gating strategy was used to analyze phosphorylated proteins of CAR T cells in Fig. 2D-G, 3F, 5K, 6E-H, 6M.

Supplementary Table 1. Prior lines of therapy.

Patient No.	Prior lines of therapy	Prior therapy
1	2	IA + IAE, DAC + Venetoclax
2	4	IA + IA, HSCT, HAA + Sorafenib, Venetoclax + midostaurin
3	6	IA + ATRA, CAG + ATRA, HA, DAC + HA, HSCT, CAG + HAG
4	4	DAE, HSCT, HA, HAA + HAG

Abbreviations: ATRA, all-trans retinoic acid; CAG, cytarabine, aclarubicin, and granulocyte-colony stimulating factor; DAC, decitabine; DAE, daunorubicin, cytarabine, and etoposide; HA, HHT and cytarabine; HAA, HHT, cytarabine, and aclarubicin; HAG, HHT, cytarabine, and granulocyte-colony stimulating factor; HSCT, hematopoietic stem cell transplantation; IA, idarubicin and cytarabine; IAE, idarubicin, cytarabine, and etoposide.

Supplementary Table 2. Characteristics of CAR T cell products.

**Patient No.**	**T-cell Origin**	**HLA Allelic Match (*n./total no.*)**	**Cell Viability (%)**	**CAR$^+$ (%)**	**Cryopreserved Before Infusion**	**Quality Control**
1	Auto	-	90.0	47.6	NO	Qualified
2	Donor	5/10	93.0	29.0	NO	Qualified
3	Donor	10/10	81.9	65.9	NO	Qualified
4	Donor	5/10	79.1	61.0	NO	Qualified
1*	Auto	-	84.4	47.6	YES	Qualified

1* Patient 1 received a second CAR T cell infusion on day 29 after the first infusion.

All CAR T cells were performed with endotoxin detection, mycoplasma test, and microscopic, and the results were qualified. To meet our product release criteria, the CAR transfection rate of CD3$^+$ T cells, as measured by flow cytometry, must be greater than 2%. This criterion has been used in many clinical trials with different T cell qualities, and in fact the CAR positivity rates of our CAR T products have been well above 2%.

Supplementary Table 3. Adverse events and grading post-CAR T cell infusion for each patient.

Adverse Event	Pt1	Pt2	Pt3	Pt4
**CRS**				
Total grading	1	4	2	2
Fever	1	1	1	1
Hypoxia	0	4	2	2
Hypotension	0	3	0	0
**ICANS**				
Total score	0	1	0	1
CAPD score	0	1	0	1
Depressed consciousness	0	0	0	0
Seizure	0	0	0	0
Motor weakness	0	0	0	0
Elevated ICP/cerebral edema	0	0	0	0
**GVHD**				
Total score	0	2	0	1
Skin	0	1	0	1
Liver	0	2	0	0
Intestinal	0	0	0	0
**Hematologic event**				
Neutropenia	4	4	4	4
Leukopenia	4	4	4	4
Thrombocytopenia	3	2	4	3
Anemia	3	3	3	3

Lymphocytopenia	3	4	4	4
**Infections**				
Virus	0	0	0	0
Bacteria	0	0	0	3
Fungus	0	0	0	0
**Gastrointestinal event**				
Abdominal distension	0	0	0	0
Vomiting	0	0	0	1
AST increased	0	3	3	3
ALT increased	2	0	2	2
Blood bilirubin increased	0	3	0	1
**Other**				
Capillary leak syndrome	0	2	1	2
Hypoalbuminemia	1	1	1	1
Hypocalcemia	2	2	2	2
Hypokalemia	1	3	1	1
Hyponatremia	1	1	1	1

Listed are all adverse events that occurred within 30 days of infusion in the four patients, regardless of whether the investigators attributed these events to CAR T-cell therapy.

Abbreviations: Pt, patient; CAPD score, Cornell Assessment of Pediatric Delirium score; CRS, cytokine release symptom; ICANS, immune effector cell-associated neurotoxicity syndrome; AST, Aspartate aminotransferase; ALT, Alanine aminotransferase.

Supplementary Table S4. Information of antibodies and other reagents.

**Antibodies**	**Fluorescein**	**Catalog number**	**Clone**	**Company**	**Dilution**
anti-human CD3	PerCP5.5	317335	OKT3	Biolegend	1/100
anti-human CD3	PE	300408	UCHT1	Biolegend	1/100
anti-human CD3	APC-Cy7	317341	OKT3	Biolegend	1/100
anti-human CD3	PE-Cy7	317333	OKT3	Biolegend	1/100
anti-human CD4	PE-Cy7	357410	A161A1	Biolegend	1/100
anti-human CD4	PE	357403	A161A1	Biolegend	1/100
anti-human CD8	PE-Cy7	344711	SK1	Biolegend	1/100
anti-human CD33	PE	366608	P67.6	Biolegend	1/100
anti-mouse CD45	PerCP	103129	30-F11	Biolegend	1/100
anti-mouse CD45	APC-Cy7	103116	30-F11	Biolegend	1/100
anti-human CD45	FITC	304006	HI30	Biolegend	1/100
anti-human CD34	PerCP	343519	581	Biolegend	1/100
anti-human CD22	APC	363505	S-HCL-1	Biolegend	1/100
anti-human CD38	PE-Cy7	356608	HB-7	Biolegend	1/100
anti-human CLL1	APC	353605	50C1	Biolegend	1/100
anti-human CD123	PE	306005	6H6	Biolegend	1/100
anti-human CD117(c-kit)	APC	375203	S18022G	Biolegend	1/100
anti-His-Tag	PE-Cy7	362620	J095G46	Biolegend	1/100
anti-His-Tag	APC	362605	J095G46	Biolegend	1/100
anti-human IgG Fc	PE-Cy7	410721	M1310G05	Biolegend	1/100
anti-human IgG Fc	APC	410711	M1310G05	Biolegend	1/100

anti-human CD366(Tim-3)	APC-Cy7	345025	F38-2F2	Biolegend	1/100
anti-human CD233(LAG-3)	BV510	369317	11C3C65	Biolegend	1/100
TOX Monoclonal Antibody (TXRX10)	PE	12-6502-82	TXRX10	eBioscience	1/100
anti-human CD62L	PE-Cy7	304821	DREG-56	Biolegend	1/100
anti-human CD45RA	APC-Cy7	304127	HI100	Biolegend	1/100
anti-human CD279(PD-1)	PE	329905	EH12.2H7	Biolegend	1/100
anti-human CD25	APC	302610	BC96	Biolegend	1/100
anti-human CD86	PE	374205	BU 63	Biolegend	1/100
anti-human CD69	PE-Cy7	310911	FN50	Biolegend	1/100
anti-human CD28	PE-Cy7	302925	CD28.2	Biolegend	1/100
anti-human CD137 (4-1BB)	APC	309809	4B4-1	Biolegend	1/100
anti-human/mouse Granzyme B Recombinant Antibody	APC	372203	QA16A02	Biolegend	1/100
anti-human IFN-γ	PerCP5.5	506527	B27	Biolegend	1/100
anti-human IL-2	PE	500306	MQ1-17H12	Biolegend	1/100
anti-human IL-21	PE	513003	3A3-N2	Biolegend	1/100
anti-human CD155	PE	337609	SKIL4	Biolegend	1/100
PVRL2 Antibody		CSB-PA006		CUSABIO	1/100

		259			
Human CD3ε Activating (OKT3) Mouse mAb		92511	OKT3	CST	1/150
Human CD28 Activating (CD28.2) Mouse mAb		91920	CD28.2	CST	1/150
Purified anti-human CD226 (DNAM-1) Antibody		338302	11A8	Biolegend	
Ultra-LEAF™ Purified anti-human CD112 (Nectin-2) Antibody		337423	TX31	Biolegend	
Purified anti-human CD96 (TACTILE) Antibody		338402	NK92.39	Biolegend	
Purified anti-human TIGIT (VSTM3) Antibody		372702	A15153G	Biolegend	
anti-human TIGIT (VSTM3)	APC	372705	A15153G	Biolegend	1/100

Antibody					
anti-human CD96 (TACTILE) Antibody	PE/Cy7	338415	NK92.39	Biolegend	1/100
anti-Lck Antibody	Alexa Fluor® 647	628303	LCK-01	Biolegend	1/100
anti-ZAP70 Antibody	Alexa Fluor® 647	691205	A 15114B	Biolegend	1/100
JNK1+JNK2+JNK3 Rabbit Monoclonal Antibody		AF1048		Beyotime	1/50
Phospho-CD247 (CD3 zeta) (Tyr142) Monoclonal Antibody (3ZBR4S), PE, eBioscience™	PE	12-2478-42	3ZBR4S	eBioscience	1/100
anti-ERK1/2 phospho(Thr202/Thr204)	PE-Cy7	369516	6B8B69	Biolegend	1/100
Phospho-c-Jun (Ser73) (D47G9) XP® Rabbit mAb		3270T	D47G9	CST	1/100
Rabbit (DA1E) mAb IgG XP® Isotype Control		3900S	DA1E	CST	1/100
Phospho-JNK1/JN		AF1762		Beyotime	1/100

K2/JNK3 (Thr183/Thr183/Thr221) Rabbit Monoclonal Antibody					
Phospho-Zap-70 (Tyr319)/Syk (Tyr352) Antibody		2701S		CST	1/100
Goat anti-Rabbit IgG	PE	HS121		TransGen Biotech	1/100
Mouse IgG2b,κ isotype Ctrl	PerCP5.5	400337	MPC-11	Biolegend	1/100
Mouse IgG1,κ isotype Ctrl	APC-Cy7	400127	MOPC-21	Biolegend	1/100
Mouse IgG1,κ isotype Ctrl	PE-Cy7	400125	MOPC-21	Biolegend	1/100
Mouse IgG1,κ isotype Ctrl	PE	400112	MOPC-21	Biolegend	1/100
Mouse IgG1,κ isotype Ctrl	APC	400119	MOPC-21	Biolegend	1/100

**Reagents**	**Catalog number**	**Company**
EasySep™ Human T Cell Enrichment Kit	19051C.2	STEMCELL
Foxp3/Transcription factor staining set	00-5523	eBioscience
250 x PMA/ ionomycin	CS1001	Multi-bioscience
DAPI (4′ ,6-diamidino-2-phenylindole)	D1306	Invitrogen
Fixable viability dye eF780	65-0865-18	eBioscience
Tag-it Violet™ Proliferation and Cell Tracking	425101	Biolegend

Dye		
BD GolgiStop™ Protein Transport Inhibitor (Containing Monensin)	554724	BD Bioscience
Annexine V Binding buffer	AB2000-H	SUNGENE BIOTECH
ImmunoCult™ Human CD3/CD28 T Cell Activator	10971	STEMCELL
Recombinant Human IL-2	200-02-100	PeproTech
Human Siglec-3 / CD33 Protein, Fc Tag (MALS verified)	CD3-H5257	Acro
Annexin V-APC/7-AAD Detection Kit	A5001-03A-L	SIMUBIOTECH
Rabbit IgG	A7016	Beyotime
BeyoClick™ EdU-488 Cell Proliferation Detection Kit	C0071L	Beyotime
Fluo-4 calcium ion detection kit	S1061S	CST
D-Luciferin,Potassium Salt. D-luciferin, potassium salt	40902ES03	YEASEN
BD Pharmingen™ 7-AAD	559925	BD Bioscience
TrueCut HiFi Cas9 Protein	A50576	Thermo
Precision Count Beads™	424902	biolegend
Human CLEC12A / MICL / CLL-1 Protein, Fc Tag (MALS verified)	CLA-H5266	Acro
Human IL-3R alpha / CD123 Protein, His Tag (MALS & SPR verified)	ILA-H52H6	Acro
LEGENDplex™ Human CD8/NK Panel (13-plex)	740267	biolegend
2-Mercaptoethanol	21985023	Thermo
L-NMMA acetate	HY-18732A	MCE
SP600125	HY-12041	MCE

U0126-EtOH	HY-12031	MCE
DAF-FM DA	S0019S	Beyotime
QuantiCyto® Human IL-15 ELISA kit	EHC013.48	NEOBIOSCIENCE

Supplementary Table S5. Characteristics of primary samples.

patient no.	organ	diagnosis	gender	age	cell type	tumor cells postive rate (%)	CD19 expression (+/-)	CD33 expression (+/-)	CD38 expression (+/-)
1	PB	B-ALL	female	5	tumor cells	55.35%	+		+
2	PB	B-ALL	male	8	tumor cells	31.70%	+		+
3	PB	AML	female	37	tumor cells	13.39%		+	
4	PB	AML	male	18	tumor cells	44.00%		+	
5	PB	AML	female	44	tumor cells	82.80%		+	
6	PB	AML	male	32	tumor cells	12.83%		+	+
7	BM	pre-B-ALL	male	25	tumor cells	40.57%	+		
8	BM	B-ALL	male	7	tumor cells	64.36%	+		
9	BM	B-ALL	female	3	tumor cells	61.70%	+		+
10	PB	AML	male	35	tumor cells	86.50%		+	
11	PB	AML	male	26	tumor cells	83.90%		+	+
12	BM	B-ALL	male	3	tumor cells	86.23%			+

13	PB	AML	male	16	tumor cells	2.22%		+	+
14	PB	AML	female	40	tumor cells	87.60%		+	+
15	BM	B-ALL	male	3	tumor cells	79.30%	+		+
16	PB	B-ALL	female	47	tumor cells	53.10%	+		+

AML: Acute Myelocytic Leukemia, B-ALL: B-cell Acute Lymphoblastic Leukemia, PB:Peripheral blood，BM：Bone marrow.

Supplementary Table S6. List of gene set related to T-cell exhaustion features.

ACP5, ADGRG1, AFAP1L2, AKAP5, ANXA5, APOBEC3C, APOBEC3G, ATP6V1C2, BATF, BST2, CCL3, CCL4, CCL4L1, CCND2, CCR1, CD27, CD27-AS1, CD2BP2, CD38, CD63, CD7, CD82, CDK2AP2, CHST12, CKS2, COTL1, COX5A, CREM, CSF1, CTLA4, CTSD, CTSW, CXCL13, CXCR6, DDIT4, DNPH1, DUSP4, DYNLL1, ENTPD1, ENTPD1-AS1, FABP5, FASLG, FKBP1A, FKBP1A-SDCBP2, FUT8, GALM, GPR25, GSTO1, GZMB, GZMH, HAVCR2, HLA-DMA, HLA-DQA1, HLA-DQB1, HLA-DRA, HLA-DRB1, HLA-DRB5, HLA-DRB6, HMGN1, HMGN3, ICOS, ID3, IDH2, IFI27L2, IFI35, IFI6, IFNG, IGFLR1, IL2RB, ISG15, ITGAE, ITM2A, KRT81, KRT86, LAG3, LAYN, LINC00299, LYST, MIR155, MIR155HG, MIR3917, MIR4632, MIR497HG, MS4A6A, MTHFD1, MTHFD2, MYO1E, MYO7A, NAB1, NDFIP2, PARK7, PDCD1, PDIA6, PHLDA1, PKM, PRDM1, PRDX3, PRDX5, PRF1, PRKAR1A, PSMB3, PSMC3, PSMD4, PSMD8, PTTG1, RAB27A, RALGDS, RANBP1, RBPJ, RGS1, RGS2, SAMSN1, SARDH, SIRPG, SIT1, SNAP47, SNRPB, SNX9, STAT3, STMN1, STRA13, SYNGR2, TIGIT, TNFRSF18, TNFRSF1B, TNFRSF9, TNFSF4, TNIP3, TOX, TPI1, TRAFD1, UBE2F, UBE2F-SCLY, UBE2L6, VAPA, VCAM1, WARS, YARS

Protocol

The authors have provided this trial protocol to provide readers with additional information about their work. It has been redacted from original Chinese documents for Journal use.

Protocol for: Zuo et al. **C-JUN overexpressing CAR-T cells in acute myeloid leukemia: preclinical characterization and phase I trial.**

CLINICAL STUDY PROTOCOL

Open-Label, Nonrandomized, Single-Arm Phase 1 Study to Evaluate the Safety and Tolerability of Functionally Enhanced CD33 CAR T Cells in Subjects with Relapsed or Refractory Acute Myeloid Leukemia

Clinical Trial Phase: I

Sponsor: Beijing Boren Hospital

Protocol Number: BRYY-IIT-LCYJ-2021-003

Version: 1.2

Effective Date: 2021.08.23

CONFIDENTIAL

This document contains confidential information, which should not be copied, released, or published without written approval from **Beijing Boren Hospital.**

Protocol Amendments

Protocol Version 1.0, dated 1 March 2021 (Original)

Summary of Key Changes:

Protocol Version 1.1, dated 23 March 2021

- **Added the patients with extramedullary relapse as meeting the inclusion criteria.**

Patients with extramedullary relapse of acute myeloid leukemia have a poor prognosis, few treatment options are available, and extramedullary lesions in leukemia patients have also responded well to CAR-T therapy in reported clinical trials, so patients with extramedullary relapse will be included in this study.

- **Specified the pretreatment chemotherapy dose and CAR-T infusion dose for Newborn and Infant (0-1 year).**

Newborn and Infant (0-1 year) has special body systems, so the pretreatment chemotherapy dose and CAR-T infusion dose are clearly stated. The pretreatment chemotherapy dose is fludarabine 30 mg/m^2 (body surface area) and cyclophosphamide 250 mg/m^2 (body surface area) infused intravenously for 3 days; the CAR-T infusion dose follows the traditional 3+3 dose escalation protocol, with dose 1 (DL-1) for 5×10^5 (±20%) CAR-T cells/kg, dose 2 (DL-2) for 1×10^6 (±20%) CAR-T cells/kg, and dose 3 (DL-3) for 5×10^6 (±20%) CAR-T cells/kg.

- **Changed "bone marrow transplantation" to "hematopoietic stem cell transplantation" in the exclusion criteria.**

Change "bone marrow transplant" to "hematopoietic stem cell transplant".

- **Changed the Ethics Committee contact person to Yan Sun.**

Protocol Version 1.2, dated 23 August 2021

- **Changed the trial design from the traditional 3+3 dose-escalation scheme to the BOIN12 (Bayesian Optimized Interval Phase I/II trial design) scheme.**

Based on recent research results and the design of several CAR-T clinical trials, the BOIN12 design is based on both toxicity and efficacy considerations to explore the optimal biological dose compared to the traditional design. The toxicity of CAR-T therapy for AML is severe, and more attention should be paid to toxicity risk control. For safety reasons, we adopted the "BOIN12" design to evaluate the efficacy and toxicity of each dose.

- **Changed the preset infusion dose from 3 dose levels to 2 dose levels.**

Due to the effectiveness of the current low-dose group and the published data from other centers showing the high toxicity of CAR T treatment in AML, the maximum dose of 3: 5×10^6 (± 20%) CAR T cells/kg was dropped for safety reasons.

- **Added "Patients required to infuse autologous CAR T cells, with a tumor load higher than 30%" to the exclusion criteria.**

High tumor load will affect the proliferation of T cells, and the quality and activity of T cells are not good. So the preparation of CAR T cells under such circumstances will greatly increase the risk of CAR T cells preparation failure, which is very unfavorable to the treatment of patients.

- **Added the use of TNF-α inhibitors.**

We have observed that the enrolled patients had a dramatic increase in TNF-α after CD33 CAR T-cell infusion, and developed severe CRS and that symptoms improved with the TNF-α inhibitors, and there were preclinical studies suggesting that early use of TNF-α inhibitors may help control CAR T-therapy associated side effects.

- **Added adverse events associated with TNF-α inhibitor to the definitions of DLT, adverse events, and serious adverse events.**

Due to the use of TNF-α inhibitors, the associated toxicity should be considered.

- **Added "management of GVHD".**

Because some patients who receive an infusion of donor-derived CAR T cells are at risk for GVHD, prevention and management of GVHD in these patients is necessary.

- **Changed the inclusion criteria to "male or female, aged 1-70 years old"**

Newborns and infants (0-1 year old) were excluded from this study due to their vulnerability and immature body systems, which make them difficult to treat with CD33 CAR T cells.

TABLE OF CONTENTS

LIST OF ABBREVIATION 11

PROTOCOL SUMMARY 15

1 INTRODUCTION 25

1.1 Background 25

2 OBJECTIVES AND ENDPOINTS 26

2.1 Objectives 26

2.1.1 Primary Objectives 26

2.1.2 Secondary Objectives 26

2.2 Endpoints 27

2.2.1 Primary Endpoints 27

2.2.2 Secondary Endpoints 27

3 STUDY POPULATION 27

3.1 Inclusion Criteria 27

3.2 Exclusion Criteria 28

3.3 Screening Failure 29

3.4 Removal from Study Criteria 30

3.4.1 Off-Study Criteria 30

3.4.2 Subject Requests to be Withdrawn from Study or Follow-Up 30

3.4.3 Management of Subject Withdrawal 30

3.4.4 Criteria for Replacement of Subjects 31

4 STUDY DRUG 31

4.1 Drug Information 31
4.2 Drug Labels 31
4.3 Drug Preparation and Verification 32
4.4 Drug Usage 32
4.5 Drug Management 32
5 STUDY DESIGN 33
5.1 Basis of Design 33
5.2 Overall Design 33
5.3 Study Scheme and Group Scale 33
5.3.1 Patient Enrolment, Drug Dose and Treatment Scheme 33
5.4 Definition of Dose-limiting Toxicities (DLTs) 41
5.5 Treatment Regimen 42
5.5.1 Premedication 42
5.5.2 CD33 CAR T Infusion Dose 42
5.5.3 Use of TNF-α Inhibitors 43
5.6 Subject Numbering 43
5.7 Clinical Procedures 44
5.7.1 Screening and Preparation Period (D-14 to D-1) 44
5.7.1.1 Screening Procedure 44
5.7.1.2 Peripheral Blood Mononuclear Cell Collection 49
5.7.1.3 Lymphodepleting Chemotherapy (D-5 to D-3) 50
5.7.2 CAR T Treatment (D0) and Observation Period (D1 to D30) 51

5.7.2.1 Before CAR T-cell Infusion 51

5.7.2.2 CD33 CAR T-Cell Infusion (D0) 52

5.7.2.3 Post Infusion Period (D1 to D30) 52

5.7.3 Post-Treatment Expanding Period (after D30) 54

5.8 Concomitant Medications 54

5.9 Treatment Compliance 55

5.10 Termination Criteria 55

6. TRIAL OBSERVATION INDICATORS 55

6.1 Safety Indicators 55

6.2 Pharmacokinetics (PK) Indicators 56

6.3 Clinical Efficacy Indicators 56

6.4 Follow-up Visits 56

7. ADVERSE EVENTS (AES) 57

7.1 Definitions of AEs 57

7.2 Obtaining Information of AEs 57

7.3 Record of AEs 58

7.4 Follow-Up of AEs 58

7.5 Categorization of AEs 58

7.6 Relationship between Study Drug and AEs 59

7.7 Serious Adverse Events (SAEs) 60

7.8 Management of SAEs 61

7.9 SAE Record and Report to IRB 61

8. DATA MANAGEMENT 61
8.1 Requirements for Data Filling 61
8.2 Requirements for Data Monitoring by Inspectors 62
8.3 Database Establishment, Data Entry and Inspection 62
8.4 Coding of Standard Terms 62
8.5 Data Locking and Exporting 62
8.6 Data Confidentiality 62
9. STATISTICAL ANALYSIS PLAN 63
10. QUALITY ASSURANCE AND QUALITY CONTORL 65
11. EMERGENCY MEASURES 65
12. INSPECTION 65
12.1 Inspections before the Start of the Trial 66
12.2 Inspections during the Trial 66
12.3 Inspections of the Trial Sites 66
13. ETHICS AND INFORMED CONSENT 67
13.1 Regulations of Ethics 67
13.2 Informed Consent 67
13.3 Confidentiality and Privacy 68
14 ADVERSE EVENTS AND RISKS MANAGEMENT 68
14.1 Adverse Events Management during leukapheresis 68
14.2 Adverse Events Management during CAR T-Cell Infusion 69
14.3 Adverse Events Management after CAR T-Cell Infusion 69

14.4 Management of CRS and ICANS 70

14.5 Management of GVHD 71

14.6 Management of Virus Activation 74

14.7 Risk Control Measures 74

15 PROTOCOL DEVIATION 75

16 PUBLICATION OF ARTICLES AND CONFLICT OF INTEREST 76

17 PTOTOCOL REVISION 76

18 REFERENCE 77

LIST OF ABBREVIATION

AE	Adverse event
ALB	Albumin
ALP	Alkaline phosphatase
ALT	Alanine aminotransferase
AML	Acute myeloid leukemia
ANC	Absolute neutrophil count
APTT	Activated partial thromboplastin time
AST	Aspartate aminotransferase
BIL	Bilirubin
BUN	Blood urea nitrogen
Ca	Calcium
CAR	Chimeric Antigen Receptor
CHO	Cholesterol
CI	Confidence interval
CK	Creatine kinase
CMV	Cytomegalovirus
CR	Complete remission
CREA	Creatinine
CRi	CR with incomplete hematologic recovery
CRP	C-reactive protein
CRS	Cytokine release syndrome

CT	Computed tomography
CTCAE	Common Terminology Criteria for Adverse Events
DBIL	Direct bilirubin
DLT	Dose-limiting toxicity
DOR	Duration of response
EBV	Epstein-Barr virus
ECG	Electrocardiogram
ECOG	Eastern Cooperative Oncology Group
eCRF	Electronic case report form
EEG	Electroencephalogram
EOT	End of treatment
FAS	Full analysis set
GCP	Good Clinical Practice
GGT	Glutamyl transpeptidase
GLP	Good laboratory practice
GLU	Glucose
GVHD	Graft-versus-host disease
HBcAb	Antibody to hepatitis B core antigen
HBD	Hydroxybutyric dehydrogenase
HBeAb	Antibody to hepatitis B e-antigen
HBeAg	Hepatitis B e-antigen
HBsAb	Antibody to hepatitis surface antigen

HBsAg	Hepatitis B surface antigen
HBV	Hepatitis B virus
HIV	Human immunodeficiency virus
HCG	Human Chorionic Gonadotrophin
HCV	Hepatitis C virus
ICF	Informed consent form
ICH	International Conference on Harmonization
IEC	Independent Ethics Committee
IP	Inorganic phosphate
INR	International standardization ratio
IRB	Institutional Review Board
LDH	Lactate dehydrogenase
MFI	Mean fluorescence intensiy
MRI	Magnetic resonance imaging
MRD	Minimum residual disease
MTD	Maximum tolerated dose
NCI	National Cancer Institute
NIT	Nitrite
ORR	Objective response rate
OS	Overall survival
PFS	Progression-free survival
PR	Partial remission

QC	Quality control
RP2D	Recommended phase II dose
SAE	Serious Adverse Event
SCT	Stem cell transplantation
SG	Specific gravity
SOP	standard operating procedure
SS	Safety Set
TBIL	Total bilirubin
TEAE	Treatment-emergent adverse event
TG	Triglyceride
TP	Total protein
TP-Ab	Treponema pallidum antibody
TT	Thromboplastin time
UBG	Urobilinogen

PROTOCOL SUMMARY

**Sponsor Name**	Beijing Boren Hospital
**Study Drug**	Functionally Enhanced CD33 CAR T cells
**Title of Study**	Open-Label, Non-randomized, Single-Arm Phase 1 Study to Evaluate the Safety and Tolerability of Functionally Enhanced CD33 CAR T Cells in Subjects with Relapsed or Refractory Acute Myeloid Leukemia
**Study Sites/facilities**	The study will be conducted at Beijing Boren Hospital
**Clinical Trial Phase**	I
**Objectives**	**Primary Objectives**:<br>To assess the safety and tolerability of functionally enhanced CD33 CAR T cells when administered intravenously (IV) in subjects with relapsed/refractory acute myeloid leukemia.<br><br>**Secondary Objectives**:<br>1. To evaluate the anti-tumor activity and toxicity of functionally enhanced CD33 CAR T cells administered in subjects with r/r AML.<br>2. To characterize the pharmacokinetic (PK) profile of functionally enhanced CD33 CAR T cells in r/r AML.
**Endpoints**	**Primary Endpoints:**

	Overall safety and tolerability profile of functionally enhanced CD33 CAR T cells. The safety profile of CD33 CAR T cells will be assessed by monitoring the adverse events (AE) per ASTCT Consensus Grading for Cytokine Release Syndrome and Neurologic Toxicity Associated with Immune Effector Cells and the Common Terminology Criteria for Adverse Events (CTCAE) Version 5.0.<br><br>**Secondary Endpoints:**<br>Efficacy: Objective response rate (ORR) according to NCCN, Version 2.2021 at 15 days, 30 days, and within three months, Duration of response (DOR) by IRR per NCCN, progression-free survival (PFS), and overall survival (OS).
**Study Design**	**Overall design:**<br>This is a single-center, open-label, non-randomized, single-arm, Phase I clinical trial to evaluate the safety and tolerability of functionally enhanced CD33 CAR T cells in patients with r/r AML. 25 subjects will be enrolled. Subjects will be pretreated with chemotherapy before the infusion of CAR T cells: about 5 days before cells infusion, the patients who planned to infuse CAR T cells were treated with fludarabine 30 mg/m^2 (body surface area) and cyclophosphamide 250 mg/m^2 (body surface area) per day for 3 days.<br>The trial design follows the BOIN12 (Bayesian Optimized Interval Phase I/II trial design) protocol. The protocol is pre-defined with 2 dose

	levels: Dose 1 (DL-1) at 5×10^5 (± 20%) CAR T cells/kg and Dose 2 (DL-2) at 1×10^6 (± 20%) CAR T cells/kg. Below the lowest dose was infused at the PI's discretion.
**Clinical Procedures**	**Screening and Preparation Period: D-14 to D-1**<br>**Screening procedure:**<br>The subjects need to sign the informed consent form before participating in the screening. The patients aged 19-70 with self-knowledge ability need to sign the informed consent voluntarily; after the legal representative (Guardian) signs the informed consent, the children aged 1-7 can be recruited; the children aged 8-18 with self-knowledge ability need to sign the informed consent voluntarily, and the legal representative (Guardian) also needs to sign the informed consent. The screening work will be carried out at about D-14, and only those subjects who meet all the inclusion criteria and do not meet the exclusion criteria are allowed to participate in the clinical trial.<br>During the screening period, demographic information, medical history, vital signs, body weight, ECOG score, physical examination, nervous system examination, 12-lead ECG, blood routine, blood biochemistry, urine routine, blood pregnancy examination (only fertile female patients), biological effect study, bone marrow examination, cranial magnetic resonance imaging (MRI) and cerebrospinal fluid examination should be collected. If there is an extramedullary lesion, the corresponding site of

	PET-CT, should be examined.<br><br>**Peripheral blood mononuclear cell (PBMC) collection:**<br><br>Subjects who meet all the inclusion criteria and do not meet any inclusion criteria will receive peripheral blood mononuclear cell collection through a blood cell separator for total peripheral blood lymphocytes of 3×10^6/kg collected from the subject or transplant donor.<br><br>CAR T cell preparation: About 5 days before infusion, the cells were cultured in a serum-free medium containing anti-CD3/CD28 antibody, IL-2, IL-7, IL-15, IL-21, transduced with humanized CD33 scFv-41BB-CD3ζ lentivirus vectors for about 5-8 days, and then the cells were washed and concentrated in 10-20 ml liquid for infusion.<br><br>**Lymphodepleting chemotherapy：**<br><br>1. After PBMCs were collected, about 5 days before cell infusion, patients planning to infuse CAR T cells were treated with fludarabine 30 mg/m^2 (body surface area) and cyclophosphamide 250 mg/m^2 (body surface area) for 3 days;<br><br>2. CAR T-cell infusion must be performed 48 hours after the completion of lymphodepletion;<br><br>3. After approval by the investigator, the chemotherapy regimen can be

	adjusted according to the tumor burden and other circumstances of the subjects.<br><br>**CAR T Treatment (Day 0) and Observation Period (Day 1 to Day 30)**<br>Functionally enhanced CD33 CAR T cells infusion:<br>Subjects would be given an intramuscular injection of 25 mg promethazine hydrochloride (12.5 mg if body weight is less than 50 kg) and a slow intravenous injection of 10ml 10% calcium gluconate about 30min before intravenous infusion of CAR T cells.<br>During the observation period, the researchers also need to complete the safety evaluation, pharmacokinetic evaluation, and efficacy evaluation of the subjects. The researchers are required to evaluate subjects based on evaluation indicators such as the safety and efficacy of the subjects.<br><br>**Post-Treatment Extending Period: after Day 30**<br>After the observation of the first month, the subjects enter the extended observation period, who are evaluated as unsuitable for SCT or other treatments that have a direct impact on the safety and efficacy evaluation of this study. Safety and disease assessments should be performed every month or whenever necessary.
**Assessments**	**Safety Indicators:**<br>Safety evaluation indexes include:

	1. Dose-limiting toxicity (DLT);<br>2. The incidence and severity of the treatment-emergent adverse event (TEAE);<br>3. Other safety indicators include vital signs, a 12-lead electrocardiogram, and clinical laboratory examination results.<br><br>**Pharmacokinetics (PK) Indicators:**<br>The evaluation index of pharmacokinetics is the expansion and persistence of functionally enhanced CD33 CAR T cells in the peripheral blood of the subjects.<br><br>**Clinical Efficacy Indicators:**<br>response evaluation criteria including:<br>1. Objective response rate (ORR), Complete remission (CR), Partial remission (PR), Duration of response (DOR);<br>2. Progression-free survival (PFS) and Overall survival (OS).
**Study Population**	**Inclusion Criteria:**<br>To be eligible to participate in this study, an individual must meet all of the following criteria:<br>1. Candidates with relapse or refractory $CD33^+$ acute myeloid leukemia, who have progressed after treatment with all standard therapies or are intolerant of standard therapy, have limited prognosis with currently

	available therapies and had no available curative treatment options (such as SCT or chemotherapy)<br>2. Male or female, aged 1-70 years<br>3. No serious allergic constitution<br>4. Eastern Cooperative Oncology Group (ECOG)[1] performance status score 0 to 2<br>5. Have a life expectancy of at least 60 days based on the investigator's judgment<br>6. CD33 positive in bone marrow or cerebrospinal fluid (CSF) by flow cytometry, or CD33 positive in tumor tissues by immunohistochemistry; (CD33 positive criteria: Flow cytometry: Positive: > 80% of tumor cells expressed CD33 and the MFI of CD33 is the same as that in normal myeloid cells; Dim: > 80% of tumor cells expressed CD33, but the MFI of CD33 is lower than that in normal myeloid cells as least as 1log; Partial positive: 20%-80% of tumor cells expressed CD33 and the MFI of CD33 is the same as that in normal myeloid cells. tumor tissue immunohistochemistry: Positive > 30% tumor cells expressed CD33)<br>7. Provide a signed informed consent before any screening procedure; subjects who voluntarily participate in the study should have the ability to understand and sign the informed consent form and be willing to follow the study visit schedule and relevant study procedure, as specified in the protocol. Candidates aged 19-70 years need to be sufficiently conscious and

	able to sign the treatment consent form and voluntary consent form. Pediatric patients aged 1-7 years could be recruited after signing an informed consent form by a legal surrogate (Guardian); pediatric patients aged 8-18 years need to be sufficiently conscious and voluntarily signed an informed consent form, and their legal surrogates (guardians) were also required to sign a written informed consent form.<br><br>**Exclusion Criteria:**<br>An individual who meets any of the following criteria will be excluded from participation in this study:<br>1. Intracranial hypertension or disorder of consciousness<br>2. Symptomatic heart failure or severe arrhythmia<br>3. Symptoms of severe respiratory failure<br>4. Complicated with other types of malignant tumors<br>5. Diffuse intravascular coagulation<br>6. Serum creatinine and/or blood urea nitrogen $\geq$ 1.5 times the normal value<br>7. Suffering from septicemia or other uncontrollable infections<br>8. Patients with uncontrollable diabetes<br>9. Severe mental disorders<br>10. Obvious and active intracranial lesions were detected by cranial magnetic resonance imaging (MRI)

11. Have received organ transplantation (excluding hematopoietic stem cell transplantation)

12. Reproductive-aged female patients with positive blood HCG test

13. Screened to be positive for infection of hepatitis (including hepatitis B and C), AIDS, or syphilis

14. Patients required to infuse autologous CAR T cells, with a tumor load higher than 30%

Off-Study Criteria

If a subject, for whatever reason is no longer appropriate to continue receiving study therapy, they will be notified and withdrawn from the study. Once a subject is taken off the study, no further data can be collected.

1. Subject requests to be withdrawn from the study for ethical consideration

2. SAEs

3. Participants will benefit from other interventions

4. Poor compliance

   a. Irregular monitoring and usage of drugs

   b. Using other anti-leukemia therapies that affect the assessment

   c. Other behaviors that can affect the trial.

Subject Requests to be Withdrawn from Study or Follow-Up:

	Participation in this study is completely voluntary. Subjects are free to withdraw from this study at any time by informing the investigator. Furthermore, if the subject is non-compliant (e.g. non-compliant with visits, and concomitant medications) they will be withdrawn from the study and a replacement subject may be recruited. The reasons for study withdrawal should be collected and documented.<br><br>**Termination Criteria**<br>1. Significant errors in the trial protocol were found during the study, making it difficult to evaluate the drug;<br>2. Under the premise of fully protecting the safety and rights of the subjects, the researcher requires termination (for example, management reasons)

1 INTRODUCTION

1.1 Background

The University of Pennsylvania's Carl June team, in 2011, reported successful treating of chronic lymphocytic leukemia with CD19-dierected CAR T cells, representing a significant milestone for CAR T-cell immunotherapy. Since then, CAR T-cell therapy has achieved remarkable breakthroughs in the treatment of B-cell originated tumors, particularly refractory and relapsed acute B lymphoblastic leukemia. Many cases have demonstrated complete remissions. Beijing Boren Hospital conducted several clinical trials targeting refractory/relapsed acute B lymphoblastic leukemia, which yielded convincing preliminary results previously reported in Blood 2019 and Leukemia 2019, 2020, featuring pertinent clinical trial data[2-4]. There is substantial clinical experience with side effect control of CAR T-related immunotherapy. CAR T cell therapy toward acute myeloid leukemia (AML) patients has not elicited satisfactory results up to date. Most AML tumor cells express CD33 antigen, which can be used for CAR-T therapy target. Liu et al. reported the efficacy and safety of CLL1-CD33 CAR T for refractory AML[5]. However, there are still challenges in the expansion and persistence of CAR T cells in r/r AML patients, which limits the application of this technology on a large scale. To evaluate the efficacy and safety of CAR T therapy, we plan to conduct a single-center, open, non-randomized, single-arm clinical trial of functionally optimized CD33 CAR T cells for the treatment of refractory or relapsed AML.

During a clinical trial of autologous CD33 CAR T cells for r/r AML, three patients did not achieve remission and suffered from severe CRS, ICANS, and other adverse events, indicating significant barriers to CAR T treatment in AML[6]. TNF-α is a cytokine produced by monocytes,

macrophages, and T cells, and is closely associated with inflammation and autoimmune diseases. It has been reported that TNF-α was dramatically elevated in mice treated with CLL-1 IL15 CAR T in an AML model, and early use of TNF-α inhibitors could control the occurrence of side effects while maintaining the antitumor effect[7], suggesting that abnormal elevation of TNF-α may be closely related to the serious adverse events of CAR T treatment of AML. TNF-α inhibitors have been widely used clinically in the treatment of rheumatoid arthritis and ankylosing spondylitis, commonly adalimumab and etanercept, preventing CAR T-mediated inflammatory toxicity.

To further investigate the efficacy and safety of CAR T therapy in AML, our center designed a single-center, open, non-randomized, single-arm clinical trial of functionally enhanced CD33 CAR T cells for refractory/relapsed acute myeloid leukemia at Beijing Boren Hospital.

The primary objective of our trial is to assess the safety and tolerability of CD33 CAR T cells when administered intravenously (IV) to patients with r/r AML. Secondary objectives are: (1) To evaluate the anti-tumor activity and toxicity of functionally optimized CD33 CAR T cells administered in patients with r/r AML; (2) To characterize the pharmacokinetic (PK) profile of CD33 CAR T cells in r/r AML.

The Ethics Committee has approved this experiment and this consent form.

2 OBJECTIVES AND ENDPOINTS

2.1 Objectives

2.1.1 Primary Objectives

To evaluate the safety and tolerability of functionally optimized CD33 CAR T cells when administered intravenously (IV) in patients with r/r AML.

2.1.2 Secondary Objectives

(1) To evaluate antitumor activity and toxicity of functionally enhanced CD33 CAR T cells administered in subjects with r/r AML;

(2) To determine the pharmacokinetic (PK) profile of functionally enhanced CD33 CAR T cells in r/r AML.

2.2 Endpoints

2.2.1 Primary Endpoints

The overall safety and tolerability of the functionally enhanced CD33 CAR T cells were as follows. The safety profile of functionally enhanced CD33 CAR T cells is being evaluated concerning severity, the incidence of adverse events (AEs) and serious adverse events (SAEs), and dose-limiting toxicities (DLTs) in subjects with $CD33^{+}$ myeloid cell malignancies in the study. ASTCT and CTCAE v5.0, EBMT consensus, laboratory assessments, vital signs, physical examinations, and ECG are used to grade and code all AEs.

2.2.2 Secondary Endpoints

Anti-tumor activity: Objective response rate (ORR), progression-free survival (PFS), and overall survival (OS) will be used to characterize preliminary evidence of anti-tumor activity.

3 STUDY POPULATION

3.1 Inclusion Criteria

To be eligible to take part in this study, an individual will need to meet all of the following criteria:

1. candidates with relapsed or refractory $CD33^+$ acute myeloid leukemia, who have progressed after treatment with all standard therapies or are intolerant of standard therapy, have limited prognosis with currently available therapies and had no available curative treatment options (such as SCT or chemotherapy).

2. Male or female, aged 1-70 years

3. No serious allergic constitution

4. Eastern Cooperative Oncology Group (ECOG)[1] performance status score 0 to 2

5. Have a life expectancy of at least 60 days based on the investigator's judgment

6. CD33 positive in bone marrow or cerebrospinal fluid (CSF) by flow cytometry, or CD33 positive in tumor tissues by immunohistochemistry; (CD33 positive criteria: Flow cytometry: Positive: > 80% of tumor cells expressed CD33 and the MFI of CD33 is the same as that in normal myeloid cells; Dim: > 80% of tumor cells expressed CD33, but the MFI of CD33 is lower than that in normal myeloid cells as least as 1log; Partial positive: 20%-80% of tumor cells expressed CD33 and the MFI of CD33 is the same as that in normal myeloid cells. tumor tissue immunohistochemistry: Positive > 30% tumor cells expressed CD33).

7. Before any screening procedure, a signed informed consent form is provided; volunteers should be able to understand and sign the informed consent form and be willing to follow the study visit schedule and procedures, as specified in the protocol. Candidates aged 19-70 years must be conscious and able to sign the consent form and the informed consent form. Pediatric patients aged 1-7 years could be recruited after signing an informed consent form by a legal surrogate (Guardian); pediatric patients aged 8-18 years need to be sufficiently conscious and

voluntarily signed an informed consent form, and their legal surrogates (guardians) were also required to sign a written informed consent form.

3.2 Exclusion Criteria

Anyone found to meet any of the following criteria will be excluded from participating in this study.

1. Intracranial hypertension or disorder of consciousness
2. Symptomatic heart failure or severe arrhythmia
3. Symptoms of severe respiratory failure
4. Complicated with other types of malignant tumors
5. Diffuse intravascular coagulation
6. Serum creatinine and/or blood urea nitrogen ≥ 1.5 times the normal value
7. Suffering from septicemia or other uncontrollable infections
8. Patients with uncontrollable diabetes
9. Severe mental disorders
10. Cranial magnetic resonance imaging (MRI) reveals obvious and active intracranial lesions
11. Have received organ transplantation (excluding hematopoietic stem cell transplantation)
12. Reproductive-aged female patients with positive blood HCG test
13. Have been screened to be positive for infection with hepatitis (including hepatitis B and C), AIDS, and syphilis
14. Patients required to infuse autologous CAR T cells, with a tumor load higher than 30%

3.3 Screening Failure

Detailed documentation, including demographic information and reasons for non-participation, should be provided for subjects who do not participate in screening. There will be no repeat screening of subjects in this clinical trial. A unique screening number is assigned to each subject participating in the screening. Do not assign different screening numbers to the same subject.

3.4 Removal from Study Criteria

3.4.1 Off-Study Criteria

If, for any reason, it is no longer appropriate for a subject to continue receiving study treatment, the subject will be notified and withdrawn from the study. No further data can be collected once a subject has been withdrawn from the study.

- Request by the subject to be withdrawn from the trial on ethical grounds
- SAEs
- Participants will benefit from other interventions
- Poor compliance
  a. Irregular monitoring
  b. Use of other anti-leukemia therapies that affect the assessment
  c. Other behaviors that can affect the trial

3.4.2 Subject Requests to be Withdrawn from Study or Follow Up

In accordance with the terms of the informed consent form, subjects are entitled to withdraw from participating, or if the subjects do not revoke their informed consent, they stop following up for various reasons (also known as withdrawing or shedding). As far as possible, the reasons for withdrawal should be known and recorded.

3.4.3 Management of Subject Withdrawal

The reason for the withdrawal of any subject should be documented. Relevant investigations and safety assessments should be completed if subjects withdraw from the clinical trial, and specific testing should be determined by the investigator.

3.4.4 Criteria for Replacement of Subjects

Participants who withdraw from the trial for the following reasons may be replaced.

1. Any subject who withdraws from the trial before the 21-day DLT evaluation for reasons other than DLT should be considered ineligible for the determination of the MTD and should be replaced.

2. After discussion between the investigator and the sponsor, subjects withdrawn for protocol violations may be replaced.

4. STUDY DRUG

4.1 Drug Information

Functionally enhanced CD33 CAR T cells were manufactured with T cells and transduced with a lentiviral vector that carries a functionally enhanced CD33 CAR construct that is composed of CD33 antigen recognition, 4-1BB costimulatory, and CD3ζ signaling domains.

The CAR construct was preclinically developed by the Dr. Xiaoming Feng laboratory, Institute of Hematology, Chinese Academy of Medical Sciences. The CD33 CAR T cells were produced and tested in the good manufacturing practices (GMP) laboratory of Beijing Boren Hospital.

4.2 Drug Labels

The investigational product will be labeled with the clinical trial number, the CAR T cell product number, the subject number, and the subject's initials, and will be marked 'for clinical trial use only'.

4.3 Drug Preparation and Verification

About 5 days before infusing, the cells were cultured in a serum-free medium containing anti-CD3/28 antibody, IL-2, IL-7, IL-15, IL-21, transduced with CD33 CAR lentivirus vector after one day, and cultured for a total of about 5-8 days. The cells were then washed and concentrated in 10-20 ml of liquid to infuse. Strict controls for endotoxin, bacteria, and mycoplasma are performed on the virus and functionally optimized CD33 CAR T cells. The person responsible for cell quality control should affix drug information labels to the qualified cell product and sign the functionally enhanced CD33 CAR T cell Final Product Quality Verification Report. The detailed procedures are listed in functionally enhanced CD33 CAR T cell Manufacture and Verification Regulations, functionally enhanced CD33 CAR T cell Verification Standard Operating Procedures, and functionally enhanced CD33 CAR T cell Manufacture, Inspection, and Quality Assurance Management System.

4.4 Drug Usage: IV

4.5 Drug Management

All trial medications will be stored in a secure, temperature-controlled, locked environment with limited access. Provided that the trial is not compromised, the sponsor may, upon request, inspect supplies, storage, dispensing procedures, and records. After completion of the functionally enhanced CD33 CAR T cell preparation, when the cell infusion was performed 3 days later, the cells were first cryopreserved and then thawed before infusion.

5 STUDY DESIGN

5.1 Basis of Design

Technical Guiding Principles for Research and Evaluation of Cell Therapeutic Products, (formulated by China State Food and Drug Administration)[8]

Considerations on Clinical Trial Design of Chimeric Antigen Receptor T Lymphocytes in the Treatment of Myeloid Malignancies (developed by the Drug Evaluation Center of the National Medical Products Administration of China)[9]

5.2 Overall Design

This is an open-label, non-randomized, single-arm, Phase 1 study of functionally optimized CD33 CAR T cells in subjects with r/r acute myeloid leukemia.

5.3 Study Scheme and Group Scale

5.3.1 Patient Enrolment, Drug Dose and Administration Scheme

This is an open-label, nonrandomized, single-arm, Phase 1 Study to evaluate the safety and tolerability of CD33 CAR T cells in patients with r/r AML. 25 subjects will be enrolled. Subjects will be pretreated with chemotherapy before infusion of CAR T cells: about 5 days before cell infusion, the patients who planned to infuse CAR T cells were treated with fludarabine 30 mg/m^2 (body surface area) and cyclophosphamide 250 mg/m^2 (body surface area) for 3 days. The trial design follows the BOIN12 protocol. The protocol is pre-defined with 2 dose levels: Dose 1 (DL-1) at 5×10^5 (± 20%) CAR T cells/kg and Dose 2 (DL-2) at 1×10^6 (± 20%) CAR T cells/kg. Once the optimal biological dose is determined in Phase I, phase II will include 10 extra cases at the OBD dose. This will enroll up to 25 patients before the trial ends.

Sequential dosing principle: the first three subjects in each dose group will be enrolled in the one-by-one dosing regimen, the second subject can be dosed only after the first subject completes the safety evaluation 14 days after dosing, and the third subject can be dosed only after the second subject completes the safety evaluation 14 days after dosing. The trial can be continued only after the third subject has completed the safety evaluation. The decision to use the sequential dosing principle for subsequent subjects was made by the investigator after the safety evaluation.

The trial design follows the BOIN12 protocol with a dose exploration phase in Phase I and a dose expansion phase in Phase II. Patients enrolled sequentially in groups of 3. The upper target toxicity limit (ϕ_T) is proposed to be 35% and the lower target efficiency limit (ϕ_E) is proposed to be 25%. P_r (π_T>ϕ_T|data) < C_T, C_T=0.95, P_r (π_E<ϕ_E|data) < C_E, C_E=0.90. Below the minimum, the dose is decided by the PI whether to infuse. Figure 1 shows the BOIN12 flow chart:

1. Treat the first group of patients at the dose1 level.

2. Calculate the incidence of DLT at the current dose, assuming that the current dose is j:

(1) If the incidence of DLT > λ_d, lower the dose to j-1 to treat the next group of patients;

(2) If the incidence of DLT ≤ λ_e, select the dose with the highest utility score from {j-1, j, j+1} to treat the next group of patients according to the desirability score table;

(3) If the incidence of DLT is between (λ_e, λ_d], calculate the number of patients N treated at the current dose j:

If N < N^*, the next group of patients is treated with the dose with the highest utility score according to the desirability score table from {j-1, j, j+1};

If N ≥ N^*, the next group of patients is treated with the dose with the highest utility score

according to the desirability score table from {j-1, j}.

3. Repeat until the number of patients treated reaches the pre-specified maximum sample size of 25, stop the trial and select OBD as the dose that is acceptable and has the highest estimated utility.

Figure 1: Flow chart of the "BOIN12" design:

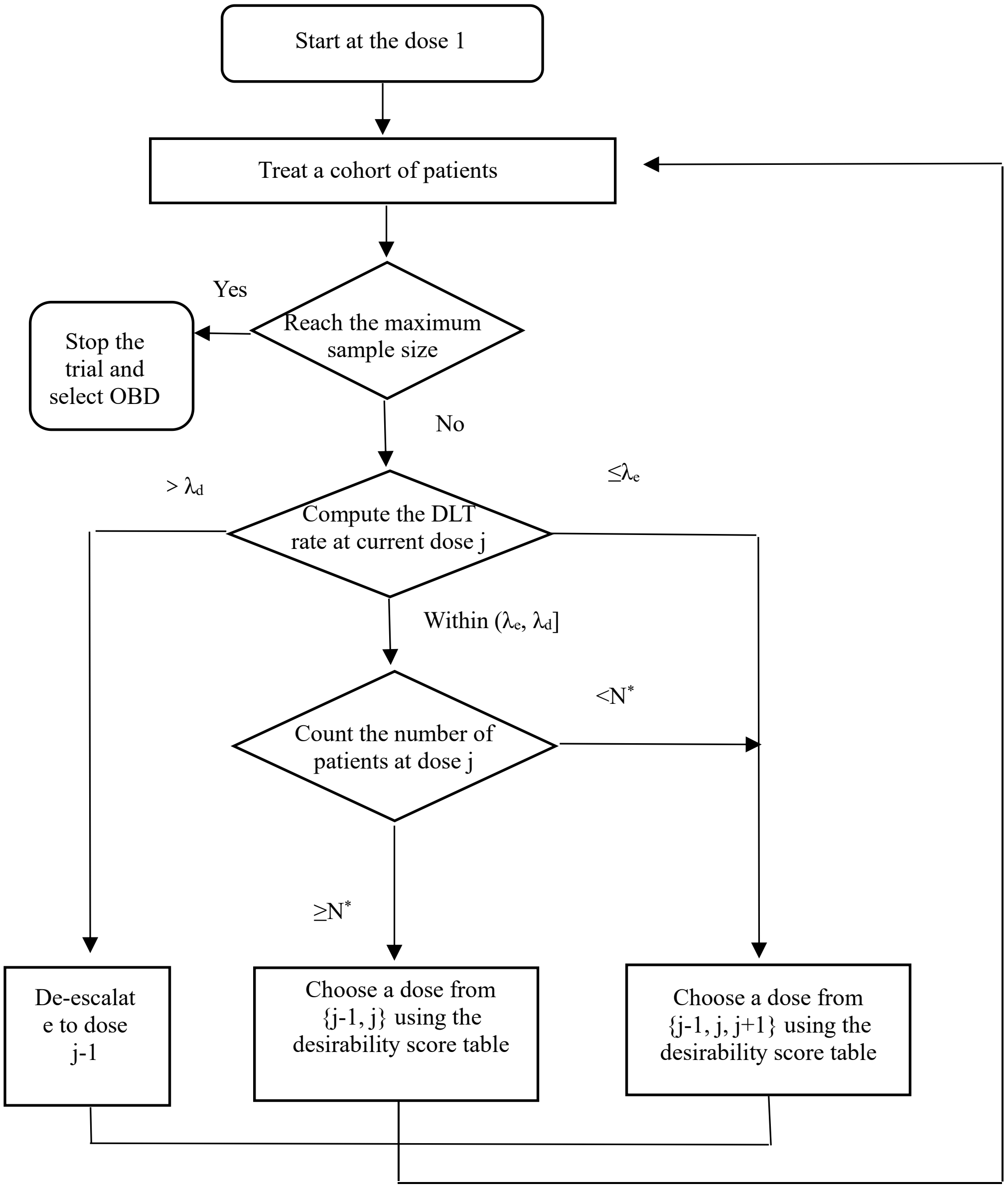

Note. λ_e=0.276 and λ_d=0.419 are escalation and de-escalation boundaries, respectively. N^*=6

Table 1: Escalation/De-escalation boundaries for the BOIN12 design

	1	2	3	4	5	6	7	8	9
Number of evaluable patients treated	1	2	3	4	5	6	7	8	9
Escalate if # of DLT≤	0	0	0	1	1	1	1	2	2
Deescalate if # of DLT≥	1	1	2	2	3	3	3	4	4
Eliminate if # of DLT≥	NA	NA	3	4	4	5	5	6	6

Table 2: Rank-Based Desirability Score (RDS) Table for the BOIN12 design.

#Pts	#Tox	#Eff	RDS		#Pts	#Tox	#Eff	RDS
0	0	0	60		9	0	6	85
3	0	0	35		9	0	7	94
3	0	1	55		9	0	8	99
3	0	2	76		9	0	9	102
3	0	3	91		9	1	≤0	E
3	1	0	24		9	1	1	17
3	1	1	44		9	1	2	29
3	1	2	63		9	1	3	40
3	1	3	80		9	1	4	53
3	2	0	13		9	1	5	65
3	2	1	31		9	1	6	78
3	2	2	48		9	1	7	88

3	2	3	69		9	1	8	97
3	≥3	Any	E		9	1	9	101
6	0	0	22		9	2	≤0	E
6	0	1	38		9	2	1	10
6	0	2	51		9	2	2	20
6	0	3	67		9	2	3	32
6	0	4	81		9	2	4	45
6	0	5	93		9	2	5	58
6	0	6	100		9	2	6	70
6	1	0	15		9	2	7	83
6	1	1	27		9	2	8	92
6	1	2	42		9	2	9	98
6	1	3	56		9	3	≤0	E
6	1	4	72		9	3	1	7
6	1	5	87		9	3	2	14
6	1	6	96		9	3	3	25
6	2	0	8		9	3	4	36
6	2	1	19		9	3	5	49
6	2	2	34		9	3	6	61
6	2	3	47		9	3	7	74
6	2	4	64		9	3	8	85

6	2	5	77		9	3	9	94
6	2	6	90		9	4	≤0	E
6	3	0	4		9	4	1	3
6	3	1	12		9	4	2	9
6	3	2	22		9	4	3	17
6	3	3	38		9	4	4	29
6	3	4	51		9	4	5	40
6	3	5	67		9	4	6	53
6	3	6	81		9	4	7	65
6	4	0	1		9	4	8	78
6	4	1	6		9	4	9	88
6	4	2	15		9	5	≤0	E
6	4	3	27		9	5	1	2
6	4	4	42		9	5	2	5
6	4	5	56		9	5	3	10
6	4	6	72		9	5	4	20
6	≥5	Any	E		9	5	5	32
9	0	≤0	E		9	5	6	45
9	0	1	25		9	5	7	58
9	0	2	36		9	5	8	70
9	0	3	49		9	5	9	83

9	0	4	61		9	≤6	Any	E
9	0	5	74					

Note. “E” means elimination. A larger value of RDS means higher desirability and any value of RDS is deemed higher than "E". #Pts denotes the number of evaluable patients treated at the current dose; #Tox denotes the number of evaluable patients who experience toxicity; #Eff denotes the number of evaluable patients who experience efficacy.

5.4 Definition of Dose-limiting Toxicities (DLTs):

Dose-limiting toxicities are defined as any CAR T cell-related ≥ grade 4 toxicity occurring within 21 days of infusion or ≥ grade 3 toxicity lasting more than 7 days, or ≥ grade 4 occurring within 21 days of infusion or ≥ grade 3 serious adverse events associated with TNF-α inhibitors lasting more than 7 days, with the following exceptions:

• Grade ≤ 4 CRS that responds to appropriate medical intervention within 3 days and recovers to grade ≤ 2

• Grade ≤ 3 neurological toxicity for < 3 days with recovery to grade ≤ 2

• Grade ≤ 4 tumor lysis syndrome lasting < 7 days

• Grade ≤ 3 neutropenia of any duration or grade 4 neutropenia lasting < 30 days

• Grade ≤ 3 anemia of any duration or grade 4 anemia lasting < 30 days

•Grade ≤ 3 thrombocytopenia of any duration or grade 4 thrombocytopenia of fewer than 30 days duration

• All cytopenias except neutropenia, anemia, and thrombocytopenia as described above.

• Fever of any grade, including febrile neutropenia

• Grade ≤ 3 diarrhea lasting < 72 hours

• Grade ≤ 3 nausea and/or vomiting lasting < 72 hours

• Grade ≤ 3 fatigue lasting < 7 days

• Grade ≤ 4 transaminases, bilirubin, creatinine kinase, blood urea nitrogen, or creatinine elevation lasting < 7 days

• Asymptomatic pancreatitis without clinical signs or symptoms of lipase elevation

• Any nonhematologic grade 3 clinical laboratory AE that is asymptomatic and rapidly reversible (returns to baseline or to grade ≤ 2 within 7 days).

• Any hematological disorders including neutropenia, anemia, thrombocytopenia, and leukopenia caused by infection or GVHD.

Definition of optimal biological dose (OBD):

OBD is defined as the minimum dose that maximizes the efficacy criteria and the OBD was explored as the recommended dose for phase 2.

5.5 Treatment Regimen

5.5.1 Premedication:

(1) After PBMCs were collected, about 5 days before cell infusion, patients planning to infuse CAR T cells were treated with fludarabine 30 mg/m^2 (body surface area) and cyclophosphamide 250 mg/m^2 (body surface area) per day for 3 days.

(2) After completion of lymphodepletion the CAR T-cell infusion must be given 48 hours later.

(3) Once approved by the sponsor, the chemotherapy regimen may be adjusted according to the tumor burden and other circumstances of the trial subjects.

5.5.2 Infusion Dose

CD33 CAR T cells are given in an inpatient setting. Based on the patient's weight (kg) and the assigned dose (/kg), the appropriate number of CAR T cells required will be calculated. The subject receives CD33 CAR T cells once intravenously. This study will be using the BOIN12 protocol with a dose exploration phase in Phase I and a dose expansion phase in Phase II. Patients enrolled sequentially in groups of 3.

CD33 CAR T cells will be given once intravenously at a standard dose of 5×10^5 cells/kg (with an allowance of ± 20%) in patients who received donor-derived or autologous CAR T cells. In cases where subjects are unable to achieve the anticipated starting dose due to issues in the preparation process, they should be assigned to the reduced dose group. This protocol aims to maximize the collection of cell numbers throughout the trial. However, if this is not feasible, investigators may still consider enrolling these subjects into the trial after careful evaluation, but it will be mandatory to document the administered dose within a minimum range of 1×10^5 CAR-T cells/kg (±20%) and a maximum range of 1×10^6 CAR-T cells/kg (±20%). Subjects who do not fall within these dose ranges will not be eligible for participation in the trial.

Redosing may be considered at the discretion of the treating physician, if complete remission was not achieved and the condition of the patients can tolerate an additional infusion.

5.5.3 Use of TNF-α Inhibitors

To control CRS, TNF-α inhibitors were used in combination with the infusion of CD33 CAR T cells. There are two types of TNF-α inhibitors: (1) etanercept, 25 mg, administered twice a week, 4 times, subcutaneously; (2) adalimumab, 20 mg, administered once every 2 weeks, subcutaneously; the changes in TNF-α levels need to be monitored while using TNF-α inhibitors. The specific duration of use is determined by the PI according to the patient's condition.

5.6 Subject Numbering

Before enrolment, each subject is identified by a screening number. S+ three digits is the screening principle for subjects in this clinical trial. According to the order in which the subjects attend the screening, the numbers will be distributed in order A '0' is added if there is no number in the hundreds and tens. For example, the first case's screening number was S001 and the tenth

case's screening number was S010. Enrolled subjects were numbered according to the order in which they were enrolled, and the trial number of the first subject was E001, followed sequentially.

5.7 Clinical Procedures

The clinical trial procedures include a screening and lymphodepletion period (D-14 to D-1), treatment and observation period (D0-D30), and extended observation period (>D30 after infusion). A timeline of the activities that will take place at each study visit with study participants is shown in Table 3. This includes all medical interventions and assessments used to determine eligibility for study participation, administration of treatment, collection of biological/biomedical specimens, decisions to discontinue study intervention, etc.

5.7.1 Screening and Preparation Period: D-14 to D-1

5.7.1.1 Screening Procedure

Before participating in the screening, subjects must sign the informed consent form. The patients aged 19-70 with self-knowledge ability need to sign the informed consent voluntarily; after the legal representative (Guardian) signs the informed consent, the children aged 1-7 can be recruited; the children aged 8-18 with self-knowledge ability need to sign the informed consent voluntarily, and the legal representative (Guardian) also needs to sign the informed consent. Screening will be carried out at approximately D-14 and only those subjects who meet all the inclusion criteria and none of the exclusion criteria will be allowed to participate in the clinical trial. In the screening period, the following tests should be evaluated:

(1) Demographic data and medical history

(2) vital signs measurements

(3) EOCG score

(4) physical examinations

(5) Examinations of the nervous system

(6) 12-lead ECG

(7) following laboratory tests

- Laboratory tests: blood routine, peripheral blood smear, plasma electrolytes, blood biochemical, urine routine, blood pregnancy test(only fertile female patients), CRP, β2-microglobulin and serum ferritin
- Coagulation tests for PT, APTT, TT, and fibrinogen
- Infectious disease screening for HBV, HCV, HIV, and TP

(8) Bone marrow examination

- cellular morphology, chromosome examination, common leukemia fusion genes (56 variants) screening, complete exon gene sequencing including blood tumor gene mutation, genetic susceptibility gene mutation analysis, flow cytometric immunoassay;

(9) Cranial MRI

(10) CSF examination: CSF routine, biochemical test, leukocyte classification counting, flow cytometry analysis of the proportion of leukemia cells

(11) Evaluable PET-CT of extramedullary tumor assessment.

(Also see summary in Table 3)

Table 3. Schedule of Assessments

Assessments[1]	Screening and Preparation Period		Treatment and Observation Period				Expanding Period
	Screening (D-14 to D-5)[2]	Lymphodepletion (D-5 to D-3)[3]	Assessment Before infusion	CAR T Cell Infusion (D0)[4]	(EOT) Visit (15 Day post last infusion)	(EOT) Visit (30 Day post last infusion)[5]	Extended observation (after D30)
Demographics, medical history	√						
Vital signs[6]	√	√	√	√	√	√	
Physical exam[7]	√	√	√	√	√	√	
ECOG performance status	√		√	√	√	√	
Oxygen saturation			√	√	√	√	
12-lead ECG[8]	√	√	√	√	√	√	
Hematology[9]	√	√	√	√	√	√	
Chemistry[10]	√	√	√	√	√	√	
Urine[11]	√			√			
Coagulation test[12]	√				√	√	
HBV, HCV,	√						

TP, HIV[13]							
EBV, CMV	√	√	√	√	√	√	√
Pregnancy test[14]	√						
Lymphocyte subsets[15]	√		√	√	√	√	
Cytokine[16]	√		√	√	√	√	
Bone marrow[17]	√		√	√	√	√	
CSF[17]	√						
functionally enhanced CD33 CAR T cells Administration[18]			√	√		√	
Radiographic tumor[19]	√					√	√
AEs[20] and con meds		√	√	√		√	√
Survival							√

1. Before conducting any protocol-specific assessments, written informed consent must be obtained.

2. Screening assessments will be performed before Lymphodepletion.

3. On D5, D4, and D3, subjects must complete daily tests before lymphodepletion chemotherapy.

4. All assessments should be performed before infusion.

5. Visits at the end of treatment should be carried out 15 days and 30 days after the infusion. All AEs will continue to be tracked until resolved.

6. Vital signs: systolic and diastolic blood pressure, respiration, pulse, and oral temperature. Vital signs should be obtained before infusing functionally enhanced CD33 CAR T cells, at the end of infusion, and after infusion.

7. At screening and the end of treatment, a full physical examination will be carried out. A directed physical examination will be performed at all other time points.

8. Before infusion, ECGs should be obtained at screening. If medically indicated, unscheduled assessments can be carried out at any time.

9. Hematology examination includes blood routine, complete blood cell count, and peripheral blood smear. Screening, baseline, and infused complete blood count and EOT visit. If medically indicated, unscheduled assessments can be carried out at any time.

10. Serum chemistries include serum electrolytes (Na, K, Cl), liver function (AST, ALT, alkaline phosphatase [ALP], γ-glutamyl transferase [GGT]), creatinine, total bilirubin, direct bilirubin, blood urea nitrogen [BUN], uric acid, albumin, total protein, albumin/globulin [A/G] ratio, glucose, CRP, β2-microglobulin and serum ferritin. Post CAR T cells infusion, hydroxybutyrate dehydrogenase [HBD], cholesterol [CHO], triglyceride [TG], calcium [Ca], and inorganic phosphate [IP] will be tested. Unscheduled assessments can be performed at any time if medically indicated.

11. Urine routine includes urine specific gravity [SG], the potential of hydrogen [PH], leukocyte [LEU], nitrite [NIT], protein [PRO], glucose [GLU], ketone [KET], urobilinogen [UBG], bilirubin [BIL], erythrocyte [ERY].

12. PT, APTT, TT, and fibrinogen are obtained on screening and EOT. If medically indicated, unscheduled assessments can be carried out at any time.

13. Virologic tests including HBsAg, hepatitis B core antibody, HCV antibody, and HIV antibody. Subjects with a negative HBsAg test and a positive HBcAb test should be quantified for HBV DNA. Subjects who are HCV antibody positive should have HCV DNA quantified.

14. A negative serum beta-human chorionic gonadotropin (β-HCG) pregnancy test must be obtained at the screening in women of reproductive potential.

15. The percentage and absolute myeloid subset count of CLL1$^+$, CD123$^+$, CD33$^+$

16. At predose, weekly, and EOT visits, cytokines including IL-6, IL-10, sCD25, INF-γ, and TNF-α will be measured.

17. Bone marrow examinations include cellular morphology, chromosome examination, common leukemia fusion genes (56 variants) screening, complete exon gene sequencing including blood tumor gene mutation, genetic susceptibility gene mutation analysis, and flow cytometric immunoassay; The persistence and count of CAR-T cells in BM will be detected at D4, D7, D11, D15, D30 and once a month after D30. If patients perform convulsions, the investigator should consider examining CAR T and tumor cells in CSF.

18. CAR T cells are planned to be administered by using a BOIN12 approach from dose 1 (DL-1): 5×10^5 (± 20%) to dose 2 (DL-2): 1×10^6 (± 20%). Below the lowest dose was infused at the PI's discretion.

19. Computed tomography (CT) or positron emission tomography (PET/CT) scans performed before informed consent (as described in the protocol) may be used within 28 days before enrollment. If it can be used consistently throughout the trial, a previous magnetic resonance imaging (MRI) scan may be used instead of a CT scan.

20. Adverse events will be collected up to 2 years post infusion or until starting subsequent treatment, whichever comes first. Only SAEs considered to be unlikely to be related to the study drug will be collected after 30 days.

5.7.1.2 Peripheral Blood Mononuclear Cell Collection

Subjects meeting all inclusion criteria and none of the exclusion criteria will receive a peripheral blood mononuclear cell collection of 3×10^{6}/kg using a blood cell separator for total peripheral blood lymphocytes. The operation was performed at Beijing Boren Hospital under the guidance of the doctor in charge of cell separation, and the PBMNC Collection and Observation Record Sheet would be filled in by the cell separator and charger. Subjects receiving PBMC must be closely monitored for symptoms and vital signs during cell collection. Adverse events and concomitant medications should be observed and recorded by the investigator, and increase the monitoring frequency of vital signs or other safety indicators per the actual situation. (Also see Summary in Table 3)

5.7.1.3 Lymphodepleting Chemotherapy (D-5 to D-3)

Before any chemotherapy pretreatment, subjects would undergo the following examinations:

(1) the weight measurement: Based on the subject's body surface area, the researchers calculated the required dose of chemotherapy drugs.

(2) Vital signs examination.

(3) laboratory examination, blood routine (blood leukocyte count, hemoglobin, blood platelet counting), the classification of white blood cells and CRP.

(4) Adverse event assessment.

Pretreatment with chemotherapy may be given to subjects who are deemed eligible by the investigator.

(1) After peripheral blood mononuclear cells (PBMCs) were collected, about 5 days before cell infusion, patients planning to infuse CAR T cells were treated with fludarabine 30 mg/m^2 (body surface area) and cyclophosphamide 250 mg/m^2 (body surface area) for 3 days.

(2) After completion of lymphodepletion, the CAR T-cell infusion must be given 48 hours later.

(3) Once approved by the sponsor, the chemotherapy regimen may be adjusted according to the tumor burden and other circumstances of the trial subjects. (Also see Summary in **Table 3**)

5.7.2 CAR T Treatment (D0) and Observation Period (D1 to D30)

5.7.2.1 Before CAR T-cell Infusion

The following tests should be evaluated:

(1) vital signs measurements

(2) Fingertip oxygen saturation

(3) AEs evaluation

(4) following laboratory tests

- Laboratory tests: blood routine, plasma electrolytes, blood biochemical, CRP, β2-microglobulin and serum ferritin
- Coagulation tests for PT, APTT, TT, and fibrinogen

(5) Biological effect test:

- The percentage and absolute cell count of $CD3^+$, $CD4^+$, $CD8^+$, $CD33^+$, central memory T cells (T_{CM}), and effector memory T cells (T_{EM}) in peripheral blood; The percentage and absolute cell count of neutrophils and monocytes
- cytokine: sCD25, IFN-γ, IL-6, IL-10, TNF-α

(6) CAR-T cell evaluation: the positive percentage and viability of harvested CAR T cells

(7) Bone marrow examination: cellular morphology of bone marrow smear, specific gene mutation analysis, flow cytometric immunoassay for MRD

(8) Specific protein quantification

Subjects deemed eligible will receive CAR T cell infusion, including leukemia relapse and whether the number of CAR T cells harvested meets dose criteria. Subjects would receive an intramuscular injection of 12.5-25mg promethazine hydrochloride and a slow intravenous injection of 10 mL 10% calcium gluconate approximately 30 minutes before intravenous infusion of CAR T cells.

5.7.2.2 CD33 CAR T cells Infusion: D0

CAR T cells are given in an inpatient setting. Based on the patient's weight (kg) and the assigned dose (/kg), the appropriate number of functionally enhanced CD33 CAR T cells required will be calculated. The subject receives CAR T cells once intravenously. This study will be using a BOIN12 approach from dose 1 (DL-1): 5×10^5 (±20%) to dose 2 (DL-2): 1×10^6 (±20%). Below the lowest dose was infused at the PI's discretion.

At the Clinical Research Centre, all dose infusions are performed under the supervision of specially trained staff. At least 24 hours after the injection, blood samples will not be taken from the same arm that was used for the intravenous injection. Subjects will be closely monitored during the infusion of the treatment and for a minimum of 2 hours after the infusion.

5.7.2.3 Post Infusion Period: D1 to D30

The following tests should be evaluated:

(1) vital signs measurements and Fingertip oxygen saturation: (once 4 hours; If abnormalities occur, 24 h life monitoring is performed until abnormal signs and/or oxygen saturation restore to a normal level. Check at least once or twice a week later);

(2) physical examinations (related to AML) (once or twice a day till performance is normal; check at least once or twice a week later).

(3) EOCG score (once or twice a day till performance is normal)

(4) 12-lead ECG: (test frequency determined by actual status; once or twice a week till performance is normal)

(5) following laboratory tests:

• Laboratory tests: blood routine, plasma electrolytes, blood biochemical (exam frequency determined by actual status; once or twice a week till performance normal)

CRP, β2-microglobulin and serum ferritin (once a week)

• Coagulation tests for PT, APTT, TT, and fibrinogen

(6) Biological effect test:

• The percentage and absolute cell count of $CD3^+$, $CD4^+$, $CD8^+$, $CD33^+$, central memory T cells (T_{CM}), and effector memory T cells (T_{EM}) in the peripheral blood; The percentage and absolute cell count of neutrophils and monocytes (once a week).

• cytokine: sCD25, IFNγ, IL-6, IL-10, TNFα (once a week)

(7) CAR-T cell evaluation: the persistence and count of CAR T cells in the peripheral blood at D4, D7, D11, D15, D21, D30, and once a month after D30. If patients perform convulsions, the investigator should consider examining CAR T cells in CSF.

(8) Blood lymphocyte subsets analysis (D15 and D30)

(9) Specific protein quantification (D30)

(10) Bone marrow examination (D15, D30) cellular morphology of bone marrow smear, specific gene mutation analysis, flow cytometric immunoassay for MRD;

(11) If there is extramedullary leukemia, PET-CT should be used to evaluate the tumor at the corresponding site: once within 30 days, then once a month until the tumor turns negative.

(12) Quantitative PCR detection of EBV and CMV viruses: once a week for 30 days and once a month afterward.

(13) adverse event assessment.

Adverse events, concomitant medications, and non-drug therapies were also observed and recorded during the observation period. During the observation period, the researchers need to evaluate the subjects according to the evaluation indexes such as the safety and efficacy of the subjects, and if the subject has a bone marrow transplant within approximately 30 days, they will be considered as withdrawals from the trial; if subjects receive a marrow transplant after approximately 30 days, the visit is considered complete.

5.7.3 Post-Treatment Expanding Period: after D30

After the first month of observation, subjects who are deemed ineligible for HSCT or other treatments that have a direct impact on the safety and efficacy evaluation of this trial will enter the extended observation period. Assessments of safety and illness should be carried out monthly or as required.

5.8 Concomitant Medications and Prohibited Medications

Some concomitant medications (ConMeds) may be used during the trial for the treatment of infusion reactions and other adverse events. During the trial, any other medication or treatment

used by the subject must be recorded in the accompanying treatment record, including the name of the medication, dosage, date of use and indications for use, etc. During the trial, you may not use any other anti-cancer therapies. Unless necessary, you should not use drugs that may interfere with the evaluation of the investigational product. Subjects receiving fresh CAR T cells were bridged to transplantation for about 30 days.

5.9 Treatment Compliance

During the screening phase, the participants or their legal representatives (guardians) should be given all the information, including the purpose of this trial, the situation of the study drug, the trial procedure, etc., and they should voluntarily participate in clinical trials. The trial subject must provide the telephone number, mailing address, and other contact information of the trial subject or legal representative (guardian) so that the researcher can notify the trial subject promptly for examination or treatment according to the trial protocol. The investigator should supervise the procedures for collecting, infusing, and testing the cells throughout the study cycle.

5.10 Termination Criteria

Trial discontinuation is the cessation of all testing in the course of a clinical trial that has not been completed per the protocol. The main purpose of stopping a trial is to protect the rights and interests of the trial subjects, to ensure the quality of the trial, and to avoid unnecessary economic losses.

(1) During the trial, significant errors were found in the trial protocol, which made it difficult for the drug to be evaluated.

(2) Under the premise of full protection of the safety and rights of the trial subjects, the researcher requests termination (e.g. for management reasons);

6. TRIAL OBSERVATION INDICATORS AND FOLLOW-UP

6.1 Safety Indicators

Safety evaluation indexes include:

1) Dose-limiting toxicity (DLT);

2) The incidence and severity of treatment-emergent adverse events (TEAE);

3) Other safety indicators include vital signs, a 12-lead electrocardiogram, and clinical laboratory examination results.

6.2 Pharmacokinetics (PK) Indicators

The index used to evaluate the pharmacokinetics is the expansion and persistence of CAR T cells present in the blood of the volunteers. On the 4th, 7th, 11th, 15th, 21st, and 30th day after infusion, CAR T cells in peripheral blood are detected by flow cytometry with recombinant CD33-Fc protein and APC-labeled anti-Fc secondary antibody. Once a month, on the 15th and 30th day after the infusion, CAR T cells are measured in the bone marrow. The percentage of CAR T cells in the lymphocytes and the absolute number of CAR-T cells in a given volume of blood are calculated. To monitor CAR-T cells with greater sensitivity, we also measure the number of copies of the CAR vector gene per microgram of genomic DNA from the peripheral blood mononuclear cells by quantitative PCR. The copy number threshold is determined to be ≥ 10.

6.3 Clinical Efficacy Indicators

response evaluation criteria including:

(1) ORR: CR, PR and DOR;

(2) PFS and OS;

6.4 Follow-up Visits

The 15-day end-of-treatment (EOT) visit following the infusion of CAR T cells was designed to evaluate the safety and efficacy of the patients. Patients will be evaluated for safety and efficacy at the 30-day EOT visit following infusion of functionally enhanced CD33 CAR T cells. Approximately 2 years after completing the infusion of functionally enhanced CD33 CAR T cells, subjects will be followed up for SAEs, confirmation of resolution of treatment-emergent AEs, and review of concomitant medications. It is acceptable to retrieve laboratory assessments performed at a facility close to the subject when laboratory assessments are needed to follow up on unresolved AEs.

Disease status follow-up visits

All subjects who responded to treatment should be followed up for disease status after CAR T cell infusion. Assessing for the disease should be done monthly or as needed.

Survival follow-up:

Each subject (including those with disease progression) will be followed for survival every 4 weeks (± 7 days) from infusion until death or withdrawal of consent. These visits may be by telephone, by e-mail, or by a visit from the person concerned or from the person's carer.

7. ADVERSE EVENTS

7.1 Definitions of Adverse Events

An AE is any adverse medical event and does not necessarily need to be causally related to lymphodepletion and CAR T cells. An adverse event can therefore be any new or aggravated

sign (including an abnormal laboratory finding for example), symptom, or disease temporally associated with the use of lymphodepletion and CAR T cells, PBMC collection, or TNF-α inhibitor treatment.

7.2 Obtaining Information of AEs

The investigator should report concisely all adverse events that have been directly observed or that have been spontaneously reported by the trial subject and those that have occurred after the period of observation should be assessed and determined by the investigator. Once the trial has started, subjects should be asked about adverse events regularly.

7.3 Record of Adverse Events

AEs during the screening period and the first dose of lymphodepletion, after lymphodepletion and 30 days after infusion, and during the extended phase (> 30 days) should be documented separately. In the extended phase, only serious AEs should be documented. According to the ASTCT 2019 consensus, CAR T cell-related AEs should be graded. According to the EBMT 2019 consensus, GVHD should be graded. Separate grades should be given for GVHD and CAR T cell-related AEs. Other AEs should be graded per CTCAE v5.0.

7.4 Follow-Up of AEs

If subjects withdraw from the trial because of the adverse event or if the adverse event is still in progress at the end of the trial, the investigator should follow up with subjects regularly, according to the clinical situation, until the adverse event is resolved, or until the subjects die, relapse, or switch to another treatment.

7.5 Categorization of AEs

Cytokine release syndrome (CRS) and neurotoxicity were graded according to ASTCT Consensus Grading for Cytokine Release Syndrome and Neurologic Toxicity Associated with Immune Effector Cells[10]. GVHD was graded according to the EBMT consensus[11]. It should be distinguished from CRS and ICANS. All AEs and clinically significant laboratory abnormalities are graded according to the Common Terminology Criteria for AEs, version 5.0[12]. For each term that is not specifically listed on the CTCAE scale, the intensity will be assigned a grade from 1 to 5 per the CTCAE guidelines:

Grade 1: Mild; asymptomatic or mild symptoms, clinical or diagnostic observations only; intervention not indicated.

Grade 2: Moderate; minimal, local, or noninvasive intervention indicated; limiting age-appropriate instrumental activities of daily living.

Grade 3: Severe or medically significant but not immediately life-threatening; hospitalization or prolongation of hospitalization indicated; disabling; limiting self-care activities of daily living.

Grade 4: Life-threatening consequences; urgent intervention indicated.

Grade 5: Death related to AE.

7.6 Relationship between Study Drug and AEs

Investigators should use what they know about the patient and the circumstances surrounding the event, and an evaluation of any potential alternative causes to determine whether or not an AE is considered to be related to the Investigational Product (IP). The following guidelines should be considered and followed:

• Temporal relationship of event onset to the initiation of Investigational Product

• Known association of the event with the study treatment or with similar treatments

• Known association of the event with the disease under study

• Presence of risk factors in the patient or use of concomitant medications known to increase the occurrence of the event

• Presence of non-treatment-related factors that are known to be associated with the occurrence of the event

Investigators should determine whether or not an AE is considered to be related to the Investigational Product and grade the co-relationship to 5 levels.

Investigators should determine whether an AE is:

• **Related:** The AE is known to occur with the study intervention, there is a reasonable possibility that the intervention caused the AE, or there is a temporal relationship between the intervention and event. Reasonable possibility means that there is evidence to suggest a causal relationship between the intervention and the AE. Positive dechallenge may have occurred.

• **Probably Related:** Reasonable evidence to suggest a causal relationship; the influence of other factors is unlikely. Positive dechallenge may have occurred.

• **Possibly Related:** Some evidence to suggest a causal relationship, but other factors may have contributed to the event.

• **Unlikely to be Related:** There is not a reasonable possibility that the intervention caused the event, there is no temporal relationship between the intervention and event onset, and an alternate etiology is likely.

• **Not Related:** It is impossible that the intervention caused the event, there is no relationship between the intervention and event onset.

7.7 Serious Adverse Events (SAE):

Serious adverse events are defined as grade 3 or higher (CTCAE version 5.0). Serious adverse events (SAEs) are any unexpected symptoms, signs, or other medical conditions that occur in subjects after PBMNC collection and cell infusion, whether or not in association with CAR T therapy.

(1) cause death or endanger life;

(2) causing permanent or severe disability or loss of function;

(3) cause congenital abnormalities or birth defects;

(4) adverse medical events, which are serious clinical events that may endanger the patient's health and require medical or surgical intervention.

7.8 Management of SAE

If subjects develop SAE, they should receive active treatment.

7.9 SAE Record and Report to IRB

The following situations require investigators to inform the IRB:

• All SAEs, except deaths due to progressive disease; In the event of an SAE, the investigator should complete, sign and date the Serious Adverse Event Report Form in as much detail as possible. The investigator must report the SAE to the hospital ethics committee, etc., within 24 hours of becoming aware of the SAE

• Any protocol deviations as required by IRB IND Safety Reports and any unexpected incidents or problems during the trial may be also reportable to the IRB according to IRB requirements.

**institution**	**Contact**	**Tel**
IRB of Beijing Boren Hospital	Yan Sun	83605200-502

8 DATA MANAGEMENT

8.1 Requirements for Data Filling

(1) The researcher should complete the form using a black pen, and handwriting should be clear;

(2) If the original records need to be amended, the researcher must amend them and no other person may amend them without authorization. Cross out the content to be modified with a single line, the modified content shall be written in the place crossed, and the name and date of modification shall be indicated. Do not use correction fluid or black for modification;

(3) All clinical trial test data should be recorded and the original report should be passed into the laboratory sheet space;

(4) To verify data that deviate significantly from clinical practice and are within the acceptable range, researchers should provide the necessary explanations. The name of the unit used shall be included in all laboratory test results;

(5) All records should be signed by the researcher upon completion and the case report form should be submitted to the principal investigator for review and signature upon completion.

8.2 Requirements of Data Monitoring by Inspectors

Regular monitoring by independent data monitors.

8.3 Database Establishment, Data Entry, and Inspection

To ensure the integrity and reliability of the data, an independent data center database and dedicated personnel are used to verify the data.

8.4 Coding of Standard Terms

None.

8.5 Data Locking and Exporting

It is responsible for independent data center databases.

8.6 Data Confidentiality

(1) All clinical trial documents and trial subject data must be submitted to the database at the Data Centre upon completion and retained by a designated person;

(2) Unless required by law, only members of the study group, sponsor, supervisor, relevant personnel of national administrative departments at all levels, relevant personnel of the hospital ethics committee, and the hospital clinical study management committee can have access to the medical records related to the subject name;

(3) In published papers or research reports, only serial numbers or initials may be used to identify the individual concerned;

(4) A confidentiality agreement is signed by all personnel participating in the clinical trial or having access to the clinical trial records.

9 STATISTICAL ANALYSIS PLAN

(This is a summary, and the full statistical analysis plan version 1.0 is in another separate document)

Filter Set: The filter set includes all patients who have signed informed consent or agreed to screen in the trial.

Baseline data collecting: All baseline data including gender, age, previous therapy history, and disease status will be collected from the screening period.

Adverse events (AEs): AEs occurring during the screening period and lymphodepletion, and until day 30 after CAR T-cell infusion, and expanding phase (> 30 days after CAR T-cell

infusion) should be documented separately. Only severe AEs are documented in the extended phase.

Efficacy analysis: CAR T cell anti-tumor activity will be also evaluated by: Preliminary evidence of treatment efficacy will be assessed in terms of ORR, PFS, and OS. ORR will be calculated as the percentage of patients with the best overall response of CR or PR and summarized by a cohort at 3 months.

PFS is defined as the time from the infusion day until disease progression or death (by any cause, in the absence of progression). In progression-free patients, PFS will be censored at the last evaluable tumor assessment (NCCN guideline). OS is defined as the elapsed time from infusion to death or the date of censoring. Patients who were alive or those lost to follow-up will be censored at the last date of contact (or the last date known to be alive). Additional censoring considerations for PFS and OS analyses will be described in the SAP. The Kaplan-Meier survival curves will also be presented.

If data is sufficient, ORR and PFS with the associated exact 2-sided 95% confidence interval will be presented. The proportion of subjects who experience the best response as CR, PR, or progressive disease will be calculated.

This project includes three cohorts for analysis as follows:

Cohort	Precondition Regimen	Planned dose of CD33 CAR T cells
1	250 mg/m^2 cylcophosphamide×3 days<br>30 mg/m^2 fludarabine×3 days	5×10^5 (± 20%) (4×10^5 up to and including 6×10^5) cells/kg
2	250 mg/m^2 cylcophosphamide×3 days<br>30 mg/m^2 fludarabine×3 days	1×10^6 (± 20%) (8×10^5 up to and including 1.2×10^6) cells/kg

Enrolled set: All enrolled subjects who received CAR T cells. The subjects who received insufficient doses of CAR T cells were also included in the enrolled set.

Full analysis set (FAS): All patients who received CAR T cells and could be evaluated within 30 days were included in FAS. The final safety assessment will include all subjects in FAS.

Safety analysis set (SS): All patients who received CAR T cells were included in SS.

10 QUALITY ASSURANCE AND QUALITY CONTROL

The applicant prepares the ethical application documents and conducts the clinical trial per the regulations and the protocol after obtaining ethical approval. The Standard Operating Procedures (SOPs) at the Beijing Boren Hospital should be made available to the monitors. Before the start of the trial, the investigator should be trained in the trial protocol so that the investigator fully understands and recognizes the specific connotation of the trial protocol and its indicators. To ensure that the conditions of the trial can meet the requirements of the protocol, the quality control personnel will review the baseline conditions. During the trial, the investigator should conscientiously carry out clinical procedures according to the requirements of the institution's standard operating procedure and the trial protocol, and make true, timely, complete, and standardized records. The quality control personnel should review the test procedure and the original records of the test. At the end of the trial, the research unit sorts out the relevant project documents, which are archived and stored after being checked by quality control personnel.

11. EMERGENCY MEASURES

The test site must be equipped with the necessary medical rescue equipment, first-aid supplies, and a standard operating procedure emergency plan. It shall be handled per the relevant standard operating procedures in the event of a medical emergency or accidental disaster.

12. INSPECTION

The investigator shall receive the necessary training to supervise and ensure that the following tasks are performed to verify that the rights and interests of all trial subjects are protected and that the trial protocol and related procedures are followed.

12.1 Inspections Before the Start of the Trial

(1) The clinical trial approval documents from the hospital ethics committee and the hospital clinical research management committee, including the approved clinical trial protocol and informed consent;

(2) Case report form;

(3) Verification and quality control procedures for the preparation of CAR T cells.

12.2 Inspections During the Trial

The inspector shall monitor the following contents every month:

(1) Subject inclusion progress;

(2) Whether the investigator is following the clinical study protocol;

(3) Informed consent signed by the subject;

(4) Filling out the case report form and other forms;

(5) All kinds of records and data preservation.

12.3 Inspections of the Trial Sites

(1) The hospital's immunotherapy laboratory has completed the preparation and verification of CD33 CAR T cells;

(2) The collection of the volunteers' cells was in the cell separation room of the hospital;

(3) Chemotherapy, CAR T infusion, and post-infusion observation should be performed in the ward and supervised by the investigator.

13. ETHICS AND INFORMED CONSENT

13.1 Regulations of Ethics

The Declaration of Helsinki and relevant regulations must be observed in the clinical trial. Before the start of the trial, IRB approval must be obtained before the clinical trial can be initiated. Any changes to this protocol during the trial must have the approval of the IRB or be documented. It is the investigator's responsibility to submit periodic interim reports to the IRB as required by the IRB and to notify the IRB of the completion of the trial.

13.2 Informed Consent

Before starting the trial, the investigator should have the IRBs written approval for the protocol and the written ICFs and any other written information to be provided to the participants. Participants were entitled to read and review the IRB-approved ICFs and other written information. The ICF should include a detailed description of the study procedures, risks and benefits, instructions, participants' rights, and compensation, if applicable. An investigator will explain the study to the participant in terms the participant understands and answers any questions the participant wants to ask. Investigators should explain their rights as research

participants, the procedures of the study, the risks and benefits, and the possible adverse effects. Participants have the right to review the written consent form and to ask any questions they may have before signing it. Before the screening period of the study, the ICF should be signed. Participants must be informed that participating is voluntary and that they are entitled to withdraw from the study at any time without prejudice. A copy of the signed informed consent form and the original informed consent form will be provided to the participant.

13.3 Confidentiality and Privacy

The investigators, their staff, and the sponsor (s) will maintain the confidentiality and privacy of the participants. This confidentiality also covers the examination of biological samples and genetic tests, in addition to the clinical information about the participants. The trial protocol, the documentation, the data, and any other information relating to the participant will be held in the strictest confidence. Without the prior written consent of the sponsor, no information about the trial or the data will be disclosed to unauthorized third parties. All of the research will be carried out in as private an environment as possible. The monitor, sponsor representatives, Institutional Review Board (IRB) representatives, and regulatory authorities have access to all documents and records maintained by the investigators.

14 ADVERSE EVENTS AND RISKS MANAGEMENT

In the event of any adverse reactions, subjects will be closely monitored during the infusion and for at least 24 hours after the infusion. Researchers should take prompt action to ensure the safety of subjects if adverse reactions occur, adverse events that may occur during the clinical trial, and how to prevent them are listed below.

14.1 Adverse Events Management during Leukapheresis

(1) In the process of circulating blood collection, the anticoagulant sodium citrate can cause hypocalcemia, which is manifested as numbness, chills, or convulsions in hands, feet, mouth, and lips;

(2) The use of sodium citrate injection for blood transfusion in circulating blood collection shall not exceed 1000 mL;

(3) 1000 ml blood was collected per cycle, and 10% calcium gluconate was taken orally 10-20 ml;

(4) Once numbness and chilliness occur in hands, feet, mouth, and lips, 10-20 mL of 10% calcium gluconate should be taken orally immediately. If the symptoms still cannot be relieved, 10 mL 10% calcium gluconate should be given for slow intravenous treatment.

14.2 Adverse Events Management during CAR T-Cell Infusion

14.2.1 Allergic reactions:

(1) Non-autologous components need to be added to the culture and preparation of cells, which may cause allergic reactions such as rash, laryngeal spasm, hypotension, and shock.

(2) Rash: Oral antihistamines.

(3) Laryngeal spasm: intravenous injection of an adrenocortical hormone, infusion, and tracheotomy if necessary.

(4) Patients with hypotension and shock: rapid intramuscular injection of epinephrine, intravenous injection of flumethasone, infusion, infusion of antihypertensive drugs, etc.

14.2.2 Chills, fever, fatigue, anorexia, systemic pain, and so on

(1) Despite adequate washing during the preparation of cell infusion, the cytokines and many of the above proteins in the supernatant of CAR T cell culture remain inevitably residual and can cause these symptoms after infusion.

(2) If the body temperature is greater than 39℃, give the antipyretic treatment such as Imathacin suppository anal plug or other antipyretic and analgesic drugs, bupleurum, and so on.

14.3 Adverse Events Management after CAR T-Cell Infusion

(1) The most common adverse event after cell infusion is cytokine release syndrome (CRS), and its main manifestations and management principles are shown in Table 4.

(2) Secondary infection of immunoglobulin deficiency: intravenous infusion of gamma globulin, and corresponding antibiotic treatment was given according to the condition;

(3) Other complications caused by chemotherapy were treated as complications after chemotherapy for leukemia.

14.4 Management of CRS and ICANS

Cytokine release syndrome (CRS) and neurotoxicity were graded according to ASTCT Consensus Grading for Cytokine Release Syndrome and Neurologic Toxicity Associated with Immune Effector Cells. Management of CRS and neurotoxicity and usage of tocilizumab were based on NCCN guidelines and the protocol of the center. Steroids (2 to 15 mg/kg/d methylprednisolone) were given by intravenous injection in patients with severe CRS (≥ grade 3) or having a high level of IL-6 (≥ 1000 pg/ml). As management of neurotoxicity, mannitol (2.5 ml/kg/dose) and furosemide (1 mg/kg/dose) was used intravenously to prevent and control intracranial hypertension. After infusion, intrathecal injection with dexamethasone (Dex) at 5 mg/dose was given when patients had symptoms or progression in the central nervous system

symptom (CNS). For patients who developed severe neurotoxicity with status epilepticus, benzodiazepine, Midazolam, and diazepam were used by intravenous injection as acute management, and antiepileptic drug therapy including Clonazepam and Dilantin was given until all symptoms were relieved. For patients who developed severe cerebral edema, intravenous dexamethasone or methylprednisolone was added besides intrathecal injection with Dex. Other common CRS symptoms and management principles are in Table 4:

Table 4. Major CRS Symptoms and Management Principles

Symptoms	Management
Fever	NSAIDs
Myalgia	NSAIDs
VLS; hypotension	Vasopressors
ARDS	CPAP
ARF	Dialysis
AHF	Cardiotonic drugs and diuresis
Dysfunction of liver	Hepatinica and PE (grade 4)
Leukopenia	Protective isolation
Infection	Pathogenic detection and antibiotics use (refer to the Sanford guide to antimicrobial therapy 2016)
Fibrinogenopenia	Replacement of fibrinogen or plasma

HLH/MAS	PE

Abbreviations: NSAIDs, non-steroidal anti-inflammatory drugs; VLS, vascular leak syndrome; ARDS, acute respiratory distress syndrome; CPAP, continuous positive airway pressure; ARF, acute renal failure; AHF, acute heart failure.PE, plasma exchange; HLH/MAS, Hemophagocytic Lymphohistiocytosis/Macrophage-activation Syndrome.

14.5 Management of GVHD

GVHD was graded according to EBMT consensus and needs to be distinguished from CRS. After the first evaluation on day 15, ruxolitinib will be given to prevent GVHD and evaluate the skin, liver, and diarrhea rejection according to the tables listed below. The interventions were given according to laboratory investigation, methylprednisolone (0.5-2 mg/kg/day) was given according to GVHD grade. If the intervention effect is poor, add mycophenolate mofetil at a dose of 0.25 g q12h. If sCD25 rises rapidly, accompanied by maculopapular skin rash within 1 day, basiliximab was used.

Table 5. Grading and Management of GVHD

stage	Skin	Liver	Intestinal tract
1	Maculopapular rash < 25% of body surface	Bilirubin 34–50 mmol/l	500 ml diarrhoea/d
2	Maculopapular rash 25–50% body surface	Bilirubin 51–102 mmol/l	1000 ml diarrhoea/d
3	Generalized erythroderma	Bilirubin 103–225 mmol/l	1500 ml diarrhoea/d
4	Generalized erythroderma with bullous formation and	Bilirubin > 255 mmol/l	Severe abdominal pain, with or without ileus

	desquamation		

Grade	Degree of organ involvement
I	Stage 1–2 skin rash; no gut involvement; no liver involvement; no decrease in clinical performance
II	Stage 1–3 skin rash; stage 1 gut involvement or stage 1 liver involvement (or both); mild decrease in clinical performance
III	Stage 2–3 skin rash; stage 2–3 gut involvement or 2–4 liver involvement (or both); marked decrease in clinical performance IV
IV	Similar to Grade III with stage 2–4 organ involvement and extreme decrease in clinical performance

Risk control measures are as follows:

(1) Subjects shall be selected in strict accordance with inclusion/exclusion criteria.

(2) The informed consent should inform the subjects of the possible risks of participating in the study.

(3) Check whether the first-aid drugs are available before the test and whether they are within the validity period, and check regularly during the test.

(4) Perfect the emergency plan before starting the trial, inform the emergency department of the hospital in advance of the adverse events that may occur in the clinical trial, and get assistance from the emergency department in case of emergency.

(5) Adverse reactions were monitored by researchers and nurses during the whole process of the test. The researcher should observe the adverse reactions of the subjects at any time, and the

adverse reactions should be treated symptomatically in time.

(6) If the adverse event or its sequelae persist, follow-up of the event is required even after the study drug is discontinued. This follow-up will continue until the adverse event or its sequelae are resolved.

(7) During the study, the doctor shall check the vital signs and general conditions of the subjects regularly, and take timely measures to deal with and record adverse events in case of any adverse events to ensure the safety of the subjects.

(8) Once serious adverse reactions occur, emergency transport shall be performed according to the relevant SOP.

14.6 Managements of Virus Activation

EBV: All patients who had a history of SCT were routinely orally given acyclovir as prophylaxis. In the patient who had EBV activation, ganciclovir (5 mg/kg q12h IV) was used continuously at the beginning of EBV activation. If the viral load was positive, the patients are treated with foscarnet sodium (60 mg/kg q8h IV) plus immunoglobulin (400 mg/kg for 5 days), and after 3 days, the virus copy number was reexamination and if the viral load was negative, the patients were treated with ganciclovir (5 mg/kg q12h IV). If the intervention effect is poor, give second-line antiviral treatment such as rituximab (375 mg/m^2).

CMV: All patients who had a history of SCT were routinely orally given acyclovir as prophylaxis. If the viral load was positive, the patients were treated with foscarnet sodium (60 mg/kg q8h IV) plus immunoglobulin (400 mg/kg for 5 days), and after 3 days, the virus copy number was reexamination and if the viral load was negative, the patients were treated with

acyclovir. If the intervention effect is poor, give second-line antiviral treatment such as CMV immunoglobulin (neutralizing antibody titer is 673 IU/ml).

BKV: The copy number of BKV in urine and blood should be detected if patients experienced urinary symptoms including frequency, urgency, and hematuria. If the viral load was positive, hydration and alkalization should be given.

Herpes virus and other viruses: the detection method is the same as CMV, acyclovir, and immunoglobulin were treated if the viral load was positive.

14.7 Risk control measures

Risk control measures are as follows:

(1) Subjects shall be selected in strict accordance with inclusion/exclusion criteria.

(2) The informed consent should inform the subjects of the possible risks of participating in the study.

(3) Check whether the first-aid drugs are available before the test and whether they are within the validity period, and check regularly during the test.

(4) Perfect the emergency plan before starting the trial, inform the emergency department of the hospital in advance of the adverse events that may occur in the clinical trial, and get assistance from the emergency department in case of emergency.

(5) Adverse reactions were monitored by researchers and nurses during the whole process of the test. The researcher should observe the adverse reactions of the subjects at any time, and the adverse reactions should be treated symptomatically in time.

(6) If the adverse event or its sequelae persist, follow-up of the event is required even after the study drug is discontinued. This follow-up will continue until the adverse event or its sequelae

are resolved.

(7) During the study, the doctor shall check the vital signs and general conditions of the subjects regularly, and take timely measures to deal with and record adverse events in case of any adverse events to ensure the safety of the subjects.

(8) Once serious adverse reactions occur, emergency transport shall be performed according to the relevant SOP.

15. PROTOCOL DEVIATION

Any intentional or unintentional deviation from the protocol during the trial can be classified as a deviation from the protocol. The number and percentage of patients with scheme deviations in the FAS set are listed according to a list of deviation categories, and the most important scheme deviations that led to the exclusion of trials are also summarised. A list is provided of all deviations from the scheme. If a deviation happens, the researcher should fill in the deviation plan record of the time of discovery, the process of occurrence of the event, the reason, and the corresponding treatment measures, and report it as required. At the end of the study, the researchers assessed the impact on the study data based on the seriousness of the discrepancy.

16. PUBLICATION OF ARTICLES AND CONFLICT OF INTEREST

The clinical trial results will be published by authors majorly from Beijing Boren Hospital and the author orders will be ranked by the principal investigator according to the contribution of the participants.

17. PROGRAMME REVISION

The IRB must review and formally approve any significant changes to the protocol.

18. REFERENCES

1. Oken, M.M., *et al.* Toxicity and response criteria of the Eastern Cooperative Oncology Group. *Am J Clin Oncol* **5**, 649-655 (1982).

2. Pan, J., *et al.* CD22 CAR T-cell therapy in refractory or relapsed B acute lymphoblastic leukemia. *Leukemia* **33**, 2854-2866 (2019).

3. Pan, J., *et al.* Frequent occurrence of CD19-negative relapse after CD19 CAR T and consolidation therapy in 14 TP53-mutated r/r B-ALL children. *Leukemia* **34**, 3382-3387 (2020).

4. Pan, J., *et al.* Sequential CD19-22 CAR T therapy induces sustained remission in children with r/r B-ALL. *Blood* **135**, 387-391 (2020).

5. Liu, F., *et al.* First-in-Human CLL1-CD33 Compound CAR T Cell Therapy Induces Complete Remission in Patients with Refractory Acute Myeloid Leukemia: Update on Phase 1 Clinical Trial. *Blood* **132**, 901-901 (2018).

6. Tambaro, F.P., *et al.* Autologous CD33-CAR-T cells for treatment of relapsed/refractory acute myelogenous leukemia. *Leukemia* **35**, 3282-3286 (2021).

7. Ataca Atilla, P., *et al.* Modulating TNFα activity allows transgenic IL15-Expressing CLL-1 CAR T cells to safely eliminate acute myeloid leukemia. *J Immunother Cancer* **8**(2020).

8. Technical Guiding Principles for Research and Evaluation of Cell Therapeutic Products, (formulated by China State Food and Drug Administration).

9. <Considerations-Development-CAR-T-Cell-Products_March-2022.pdf>.

10. Lee, D.W., *et al.* ASTCT Consensus Grading for Cytokine Release Syndrome and

Neurologic Toxicity Associated with Immune Effector Cells. *Biol Blood Marrow Transplant* **25**, 625-638 (2019).

11. Przepiorka, D.*, et al.* 1994 Consensus Conference on Acute GVHD Grading. *Bone Marrow Transplant* **15**, 825-828 (1995).

12. Common Terminology Criteria for Adverse Events (CTCAE) Version 5.0.

Open-Label, Nonrandomized, Single-Arm Phase 1 Study to Evaluate the Safety and Tolerability of Functionally Enhanced CD33 CAR T Cells in Subjects with Relapsed or Refractory Acute Myeloid Leukemia

Statistical Analysis Plan

**Protocol Number：**	**BRYY-IIT-LCYJ-2021-003**
**Protocol Version：**	**1.2**
**Plan number：**	**BRYY-IIT-LCYJ-2021-003**
**Plan Version：**	**1.1**
**Date：**	**2021.08.23**

Trial Sponsor: Beijing Boren Hospital

Assist in the development of statistical analysis plan Unit: Tianjin Yiqi Pharmaceutical Technology Development Co., Ltd

Statistical Analysis Plan Amendments

Statistical Analysis Plan Version 1.0, dated 1 March 2021 (Original)

Summary of Key Changes:

Statistical Analysis Plan Version 1.1, dated 23 August 2021

- **Changed the trial design from the traditional 3+3 dose-escalation scheme to the BOIN12 (Bayesian Optimized Interval Phase I/II trial design) scheme.**

Based on recent research results and the design of several CAR-T clinical trials, the BOIN12 design is based on both toxicity and efficacy considerations to explore the optimal biological dose compared to the traditional design. The toxicity of CAR-T therapy for AML is severe, and more attention should be paid to toxicity risk control. For safety reasons, we adopted the "BOIN12" design to evaluate the efficacy and toxicity of each dose.

- **Changed the preset infusion dose from 3 dose levels to 2 dose levels.**

Due to the effectiveness of the current low-dose group and the published data from other centers showing the high toxicity of CAR T treatment in AML, the maximum dose of 3: 5×10^6 (± 20%) CAR T cells/kg was dropped for safety reasons.

Table of Contents

1. STUDY OVERVIEW 6

2. STATISTICAL ANALYSES 14

3. DATA ANALYSIS GENERAL CONSIDERATIONS 18

4. SUBJECT DISPOSITION AND CHARACTERISTICS 19

5. MEDICAL HISTORY 21

6. SAFETY ANALYSES 23

7. ANALYSES OF SERUM PHARMACOKINETIC PARAMETERS 28

8. EFFICACY ANALYSES 28

9. DECISION FOR MISSING ROUTINE DISEASE ASSESSMENT 37

10. REPORTING OF PK PARAMETERS FOR DESCRIPTIVE STATISTICS 37

11. REFERENCES 39

ABBREVIATIONS

Abbreviations	Full Name
PAS	Pharmacokinetic set
CAR T	Chimeric antigen receptor T cell
ES	Enrolled set
FAS	Full analysis set
SS	Safety set
PAS	Pharmacokinetic set
PPS	Per protocol set
DLT	Dose-limiting toxicity
TEAE	Treatment-emergent adverse event
CR	Complete remission
ANC	Absolute Neutrophil count
CRi	Complete remission with incomplete hematologic recovery
PR	Partial remission
ORR	Overall response rate
SCT	Stem cell transplantation
DOR	Duration of remission
PFS	Progressive free survival
OS	Overall survival
CIF	Cumulative incidence function
IRC	Independent review committee
eCRF	Electronic case report form

**Abbreviations**	**Full Name**
LP	Lumbar puncture
ATC	Anatomical Therapeutic Chemical
EOT	End of Treatment

This statistical analysis plan applies only to the protocol " Open-Label, Non-randomized, Single-Arm Phase 1 Study to Evaluate the Safety and Tolerability of Functionally Enhanced CD33 CAR T Cells in Subjects with Relapsed or Refractory Acute Myeloid Leukemia" (version number: 1.2, version date: 2021.08.23).

1. STUDY OVERVIEW

1.1 Background

The University of Pennsylvania's Carl June team, in 2011, reported successful treatment of advanced CLL with CD19 CAR T, which generated global buzz and marked a significant milestone for CAR T-cell immunotherapy. Since then, CAR T-cell therapy has achieved remarkable breakthroughs in the treatment of B-cell malignancies, particularly refractory and relapsed acute B lymphoblastic leukemia. Many cases have demonstrated complete remissions. Beijing Boren Hospital conducted several clinical trials targeting refractory/relapsed acute B lymphoblastic leukemia, which yielded promising preliminary results published in Blood 2019 and Leukemia 2019-2020, featuring pertinent clinical trial data[1-3]. There is substantial clinical experience with side effect control of CAR T-related immunotherapy. Most AML tumor cells express CD33 antigen, which can be used for CAR-T therapy. Liu et al. first reported the efficacy and safety of CLL1-CD33 CAR T for refractory AML[4]. However, there are still challenges in the expansion and persistence of CAR T cells in r/r AML patients, which limits the large-scale application of this technique. To assess the efficacy and safety of CAR T therapy, we design a single-center, open, non-randomized, single-arm clinical trial of functionally enhanced CD33 CAR T cells in the treatment of refractory or relapsed AML.

During a clinical trial of autologous CD33 CAR T cells for r/r AML, three patients did not achieve remission and suffered from severe CRS, ICANS, and other adverse events, indicating significant barriers to CAR T treatment in AML[5]. TNF-α is a cytokine produced by monocytes, macrophages, and T cells, and is closely associated with inflammation and autoimmune diseases. It has been reported that TNF-α was dramatically elevated in mice treated with CLL-1 IL15 CAR T in an AML model, and early use of TNF-α inhibitors could control the occurrence of side effects while maintaining the antitumor effect[6], suggesting that

abnormal elevation of TNF-α may be closely related to the serious adverse events of CAR T treatment of AML. TNF-α inhibitors have been widely used clinically in the treatment of rheumatoid arthritis and ankylosing spondylitis, commonly adalimumab and etanercept, preventing CAR T-mediated inflammatory toxicity.

To further investigate the efficacy and safety of CAR T therapy in AML, our center designed a single-center, open, non-randomized, single-arm clinical trial of functionally optimized CD33 CAR T cells for refractory/relapsed acute myeloid leukemia at Beijing Boren Hospital.

1.2 Overall design

The overall design was a single-center, open, non-randomized, single-arm trial.

1.3 Primary Objectives

To assess the safety and tolerability of CD33 CAR T cells when administered intravenously (IV) in subjects with r/r AML.

1.4 Secondary Objectives

(1) To evaluate the anti-tumor activity and toxicity of CD33 CAR T cells administered in subjects with r/r AML;

(2) To characterize the pharmacokinetic (PK) profile of CD33 CAR T cells in r/r AML.

1.5 Study drugs

CD33 CAR T cells are prepared and verified in the Immunotherapy Laboratory and Laboratory Department of Beijing Boren Hospital.

1.6 study populations and sample size

This trial will enroll up to 25 patients with r/r AML to evaluate the safety and tolerability of CD33 CAR T cells. The trial design follows the BOIN12 (Bayesian Optimized Interval Phase I/II trial design) protocol. The protocol is pre-defined with 2 dose levels: Dose 1 (DL-1) at 5×10^5 (± 20%) CAR T cells/kg and Dose 2 (DL-2) at 1×10^6 (± 20%) CAR T

cells/kg. Once the optimal biological dose is determined in Phase I, Phase II will include 10 extra cases at the OBD dose. This will enroll up to 25 patients before the trial ends.

Sequential dosing principle: the first three subjects in each dose group will be enrolled in the one-by-one dosing regimen, the second subject can be dosed only after the first subject completes the safety evaluation 14 days after dosing, and the third subject can be dosed only after the second subject completes the safety evaluation 14 days after dosing. The trial can be continued only after the third subject has completed the safety evaluation. The decision to use the sequential dosing principle for subsequent subjects was made by the investigator after the safety evaluation.

The trial design follows the BOIN12 protocol with a dose exploration phase in Phase I and a dose expansion phase in Phase II. Patients enrolled sequentially in groups of 3. The upper target toxicity limit (ϕ_T) is proposed to be 35% and the lower target efficiency limit (ϕ_E) is proposed to be 25%. P_r ($\pi_T>\phi_T$|data) < C_T, C_T=0.95, Pr ($\pi_E<\phi_E$|data) < C_E, C_E=0.90. Below the minimum, the dose is decided by the PI whether to infuse. Figure 1 shows the BOIN12 flow chart:

1. Treat the first group of patients at the dose1 level.

2. Calculate the incidence of DLT at the current dose, assuming that the current dose is j:

(1) If the incidence of DLT > λ_d, lower the dose to j-1 to treat the next group of patients;

(2) If the incidence of DLT ≤ λ_e, select the dose with the highest utility score from {j-1, j, j+1} to treat the next group of patients according to the desirability score table；

(3) If the incidence of DLT is between (λ_e, λ_d], calculate the number of patients N treated at the current dose j：

If N < N^*, the next group of patients is treated with the dose with the highest utility score according to the desirability score table from {j-1, j, j+1};

If $N \geq N^*$, the next group of patients is treated with the dose with the highest utility score according to the desirability score table from {j-1, j}.

3. Repeat until the number of patients treated reaches the pre-specified maximum sample size of 25, stop the trial and select OBD as the dose that is acceptable and has the highest estimated utility.

Figure 1: Flow chart of the "BOIN12" design:

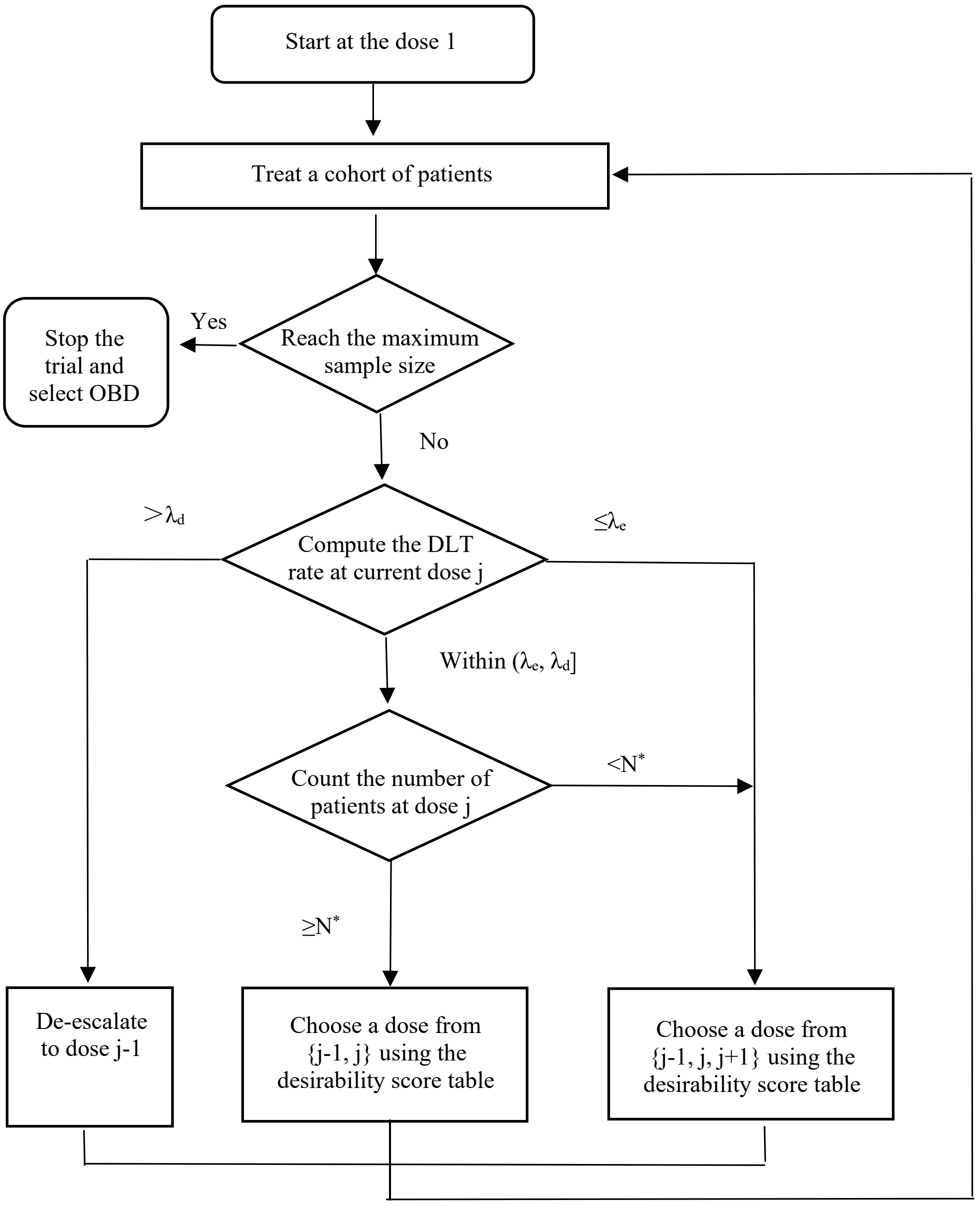

Note. λ_e=0.276 and λ_d=0.419 are escalation and de-escalation boundaries, respectively. N^*=6

Table 1: Escalation/De-escalation boundaries for the BOIN12 design

	**1**	**2**	**3**	**4**	**5**	**6**	**7**	**8**	**9**
Number of evaluable patients treated	1	2	3	4	5	6	7	8	9
Escalate if # of DLT≤	0	0	0	1	1	1	1	2	2
Deescalate if # of DLT≥	1	1	2	2	3	3	3	4	4
Eliminate if # of DLT≥	NA	NA	3	4	4	5	5	6	6

Table 2: Rank-Based Desirability Score (RDS) Table for the BOIN12 design.

**#Pts**	**#Tox**	**#Eff**	**RDS**		**#Pts**	**#Tox**	**#Eff**	**RDS**
0	0	0	60		9	0	6	85
3	0	0	35		9	0	7	94
3	0	1	55		9	0	8	99
3	0	2	76		9	0	9	102
3	0	3	91		9	1	≤0	E
3	1	0	24		9	1	1	17
3	1	1	44		9	1	2	29
3	1	2	63		9	1	3	40
3	1	3	80		9	1	4	53
3	2	0	13		9	1	5	65
3	2	1	31		9	1	6	78
3	2	2	48		9	1	7	88
3	2	3	69		9	1	8	97
3	≥3	Any	E		9	1	9	101
6	0	0	22		9	2	≤0	E

6	0	1	38		9	2	1	10
6	0	2	51		9	2	2	20
6	0	3	67		9	2	3	32
6	0	4	81		9	2	4	45
6	0	5	93		9	2	5	58
6	0	6	100		9	2	6	70
6	1	0	15		9	2	7	83
6	1	1	27		9	2	8	92
6	1	2	42		9	2	9	98
6	1	3	56		9	3	≤0	E
6	1	4	72		9	3	1	7
6	1	5	87		9	3	2	14
6	1	6	96		9	3	3	25
6	2	0	8		9	3	4	36
6	2	1	19		9	3	5	49
6	2	2	34		9	3	6	61
6	2	3	47		9	3	7	74
6	2	4	64		9	3	8	85
6	2	5	77		9	3	9	94
6	2	6	90		9	4	≤0	E
6	3	0	4		9	4	1	3
6	3	1	12		9	4	2	9
6	3	2	22		9	4	3	17
6	3	3	38		9	4	4	29

6	3	4	51		9	4	5	40
6	3	5	67		9	4	6	53
6	3	6	81		9	4	7	65
6	4	0	1		9	4	8	78
6	4	1	6		9	4	9	88
6	4	2	15		9	5	≤0	E
6	4	3	27		9	5	1	2
6	4	4	42		9	5	2	5
6	4	5	56		9	5	3	10
6	4	6	72		9	5	4	20
6	≥5	Any	E		9	5	5	32
9	0	≤0	E		9	5	6	45
9	0	1	25		9	5	7	58
9	0	2	36		9	5	8	70
9	0	3	49		9	5	9	83
9	0	4	61		9	≤6	Any	E
9	0	5	74					

Note. “E” means elimination. A larger value of RDS means higher desirability, and any value of RDS is deemed higher than "E". #Pts denotes the number of evaluable patients treated at current dose; #Tox denotes the number of evaluable patients who experience toxicity; #Eff denotes the number of evaluable patients who experience efficacy.

2. STATISTICAL ANALYSES

2.1 Analysis Sets

2.1.1 Screening sets

The screening set includes all patients who have signed informed consent and are being screened in the study.

2.1.2 Enrolled Set (ES)

The enrolled set included all subjects who participated in the study. The enrollment date was defined as the time when the patient met all clinical inclusion/exclusion criteria and received the first non-zero dose of lymphodepleting chemotherapy.

2.1.3 Full Analysis Set (FAS)

All patients who received CAR T cells are included. The final efficacy analysis will include patients in the FAS set.

2.1.4 Safety Analysis Set (SS)

All patients who received CD33 CAR T cells are included. The final safety analysis will include patients in the SS set. Note that the definition of FAS is the same as that of SS in this study.

2.1.5 Per Protocol Set (PPS)

The per-protocol set consisted of a subset of patients in the FAS who met the primary criteria for the study protocol.

The main protocol differences that led to the exclusion from PPS include:

- Disease other than AML diagnosed at baseline;
- Patients whose infusion dose did not reach the planned dose;
- Missing or incomplete disease records at baseline;

- CAR T cells from the patient or his or her transplant donor were infused into the patient without either (A) meeting all approved manufacturing criteria or (B) the time to infusion was less than 3 days from the last lymphodepleting chemotherapy.

2.1.6 Pharmacokinetic Analysis Set (PAS)

The pharmacokinetic analysis set consists of a subset of FAS patients who have at least one sample that provides assessable PK data (peripheral blood CAR T cell flow assay values and PCR copy number) for CAR T cell survival and expansion assessment. For correlation analysis of PK data, PAS will be used.

2.2 Study Follow-up

A 15-day EOT follow-up after infusion of CAR T cells should assess safety and efficacy.

A 30-day EOT follow-up after infusion of CAR T cells should be completed before initiation of subsequent transplantation.

Disease status follow-up: Patients who respond after CAR T-cell infusion should be followed for disease status. Monthly tumor assessments should be performed.

Survival follow-up: Each patient (including those with disease progression) will be followed up every 4 weeks (±7 days) for survival from infusion to death or withdrawal from the trial. The follow-up can be conducted by phone or email to the subject or their guardians.

2.3 Study drug use and study treatment

Study drug: patients or their transplant donor-derived CAR T cells.

Study treatment: This includes not only study drugs, such as CD33 CAR T cells but also non-zero dose lymphodepleting chemotherapy.

2.4 Date of the first receiving lymphodepleting chemotherapy

The date of first receiving lymphodepleting chemotherapy was defined as the first day of non-zero dose lymphodepleting chemotherapy and the index recorded in the "concomitant antitumor therapy" eCRF as "lymphatic clearance".

2.5 Date of CAR T cell infusion

The date of cell infusion is defined as receiving a non-zero dose of the study drug (CD33 CAR T cells) infusion and is recorded in the eCRF as a "drug".

2.6 Study days

The number of study days was defined as the difference between the date of the first infusion to CD33 CAR T cells (Day 0) and the evaluation date. If the evaluation occurs on or after the date of cell infusion, the number of study days will be the evaluation date - the date of cell infusion (Day 0). If the evaluation occurs before the date of cell infusion, the number of study days will be the evaluation date - the date of infusion (note: if the time occurs before cell infusion, the number of study days will be a negative number). For those patients who did not receive CAR T cell infusion, their study days will not be counted.

2.7 Baseline Definition

For the baseline definition of disease assessment, the baseline assessment was the closest measurement before the time of enrollment.

If both bone marrow aspiration and biopsy morphology results are obtained, the highest tumor cell burden will be considered and the corresponding assessment date will be used as a reference for the other assessments.

For the baseline definition of safety assessment (e.g., laboratory tests or vital signs tests), the baseline value is defined as the final measurement before the time of lymphodepleting chemotherapy.

2.8 Last contact date

The last contact date will be used as the censored date in the overall survival analysis.

For those patients whose survival is uncertain at the end date of the analysis, the last contact date should be designed to be on or before the end date as determined by the first column of Table 3. For each data source, at least the conditions to be met are listed in the second column of Table 3.

Table 3 Last contact date data source

Data source	Situations
Date of patient's last survival as recorded on the survival follow-up page	No
From the start/end date of further anti-tumor therapy	No missing medication/procedure terms
Start/end date of record page from which the drug was received	No missing drug names
Date of assessment of any special efficacy indicators available	Assessment results cannot be missing
Date of laboratory/PK data collection	Collection samples with non-missing values
Date of Vital Signs	Parameter values for at least one non-missing value
Date of performance status	Non-missing performance status
Date of AE start/end date	Terms for non-missing AE

2.9 Missing visits

For time-related response analyses (e.g., DOR, EFS), patients were considered censored if they discontinued the study due to missed visits.

2.10 Collection of data in the analysis

The final analysis of the primary and secondary endpoints of assessment will be performed after all treated patients with CD33 CAR T cells have completed the 30-day assessment or are in early discontinuation of the trial.

2.11 Time-to-event definition

The generic definition of the time event interval for the calculation.

Time-Event = Events occurrence date - start date (days)

If no post-baseline assessment results are available, the date of cell infusion will be defined as the end date, which is considered the deletion time of the last post-baseline assessment. The time-to-event variable will never have a negative value.

The deletion time is usually determined based on the appropriate response assessment date. Any response assessment is considered sufficient if the assessment is conducted and the result is not "unknown" or "incomplete".

3. Data Analysis General considerations

3.1 Overall consideration

Statistical analyses were completed using SPSS 26 or higher.

If not otherwise specified, one decimal place was retained for raw data; mean, standard deviation, median, minimum, and maximum values were retained to the same number of decimal places as the original data, up to a maximum of three decimal places; upper and lower confidence interval limits were retained to the same number of decimal places as the point estimates; and percentages were retained to one decimal place, or if the percentage was zero, this expression was ignored in the results.

If not specified, unplanned visits and off-site visits were excluded from the summary analysis for continuous variables (except when the baseline was an unplanned visit).

All available data are presented in a list sorted by center number, unique subject number, visit number, and visit date.

3.2 Statistical methods and test standards

For descriptive statistics of continuous variables, the mean, standard deviation, median, minimum, and maximum values were used; for enumeration and ranked data, the number of cases and percentages were provided; for time-to-event data, the Kaplan-Meier method was mostly used, providing the median survival time as well as the 95% confidence interval method.

3.3 Handling of missing values and outliers

All analyses are based on observed data, and filling in missing data is not considered; outliers, if any, will be discussed at the data review meeting to determine the handling method. If there are ">", "<", "≤", or "≥" values in the data, they are listed directly in the list, and when aggregating and analyzing, the analysis will be handled according to the values after removing the mathematical operation symbols.

The date format will be presented in IOS 8601 date format (MM/DD/YYYY). If the date has only a year and a month, the date will be presented in MM/YYYY format. If the date has only the year, the format will be YYYY, unless otherwise specified, and if the patient is missing a date because it is not applicable, the output will be "NA".

4. SUBJECT DISPOSITION AND CHARACTERISTICS

4.1 Subject disposition and analyzed population

Subjects volunteering to participate in the clinical trial will be screened and those who qualify will be screened into the study; the number and percentage of subjects who delayed receiving an infusion, received an infusion, completed the study, or withdrew from the study and the reasons for this will be counted; the reasons for each patient's screening failure will

be summarized and listed. The number of subjects in each analysis set will be provided according to the T cell groups receiving different donor sources with "all" columns. A list of subject distribution will also be provided.

4.2 Protocol deviation

The number and percentage of patients with protocol deviations in the FAS set will be listed via a list of deviation categories and will also summarize the major protocol deviations in the PPS set that led to exclusion from the trial.

A list of all protocol deviations will be provided.

4.3 Inclusion/exclusion criteria

Provide data that do not meet the inclusion or meet exclusion criteria.

4.4 Demographic and Baseline Characteristics

4.4.1 Demographic Data

Based on the FAS, descriptive key statistics for demographic and baseline characteristics will be provided by group and "all" columns. For continuous variables: age, the number of cases, mean, standard deviation, median, minimum, and maximum values are provided. For enumeration and ranked data: gender, provide the number and percentage of subjects in each category and provide a detailed list. The last demographic data before dosing, used as a baseline, was used for statistical analysis.

4.4.2 Medical history and AML disease characteristics

- Medical history and conditions, including symptoms at the time of informed consent, will be summarized and listed. Ongoing and historical medical conditions will be listed separately in the list. Summaries will be categorized by major system organ category and preferred terminology.
- To summarize the baseline tumor CD33 expression levels, MRD status, and morphological status derived from bone marrow assessment.

- Disease type (including ETP subgroups, tumor cell surface molecular expression characteristics).
- Aggregated CNS invasion, and extramedullary disease status of all AML patients at baseline.

4.4.3 Prior anti-tumor therapy

All previous anti-tumor drugs, radiotherapy, and SCT will be listed. The number of prior therapies will also be listed. For previously transplanted patients, the following indicators will be tabulated for the most recent transplant: time since enrollment, donor type, stem cell source (bone marrow or peripheral blood), and pretreatment regimen.

At the beginning of the study, patients will also be classified and aggregated according to their response status:

- Patients with primary refractory disease: if the patient has never had a morphological complete remission or an extramedullary lesion that has not achieved remission before the study.
- Patients with persistent refractory MRD positive disease: Patients who have been morphologically in complete remission without extramedullary lesions (or extramedullary lesions in complete remission) until the study, but whose tumor cells are detectable in the bone marrow by flow cytometry (not 0), excluding here patients who are MRD negative by flow cytometry, but positive for the fusion gene.
- Relapsed disease: Patients who are in complete remission from other therapies and have relapsed before the study, including bone marrow relapse and extramedullary relapse.

4.4.4 Cytogenetic abnormalities

The number and percentage of patients with cytogenetic abnormalities (yes/no) including fusion and mutant genes and with complex karyotypes (as defined by the criteria by

our laboratory) at the time of study entry are summarized. All cytogenetic abnormalities will be listed.

5. MEDICAL HISTORY (study treatment, emergency drugs, other concomitant therapies, and compliance)

- Prior and combined medications and meaningful non-pharmacologic treatments will be listed by the patient and described in summary by ATC category with preferred terminology before and after the start of cell infusion.
- Anti-tumor therapy (including lymphatic clearance) is received after enrollment but before the infusion of cells. The type of lymphatic clearance and the tumor burden (bone marrow or peripheral blood morphology and extramedullary tumor burden) of the patient after lymphatic clearance will also be summarized.
- The number of infused CD33 CAR T cells (cell count and cell count/kg) will be listed and summarized using descriptive statistics, the weight provided to the CD33 CAR T cells manufacturing laboratory will be used to calculate the weight-adjusted dose, and the transfection rate and viability of the CD33 CAR T cells will be recorded, whether it has been frozen or not, and if the frozen cells are infused, the cell count, viability and transfection rate of the CD33 CAR T cells will need to be recalculated after recovery. Due to the characteristics of donor CAR T cells, the HLA match will also be recorded.
- For severe CRS due to CD33 CAR T cells that will be treated with anti-cytokine drugs, the number of patients requiring anti-cytokine drugs for CRS will be summarized. The frequency and dose of drugs used will also be summarized in a list by preferred terminology.
- For GVHD due to donor CAR T cells that will be treated with anti-rejection drugs, the number of patients requiring anti-rejection drugs for GVHD will be summarized. The

frequency and dose of drugs used will also be tabulated and summarized by preferred terminology.

- Patients with abnormal hematology will require supportive hematology treatment after a 30-day efficacy assessment, and treatment will be summarized for each individual separately.
- For patients presenting with infection, the treatment will be summarized.
- Because CD33 CAR T cells are administered by a single infusion, no specific compliance with treatment other than CD33 CAR T cells needs to be summarized.
- For patients receiving a subsequent transplant, the following indicators will be tabulated: time of transplantation, donor type, stem cell source (bone marrow or peripheral blood), and pretreatment regimen.

6. SAFETY ANALYSIS

Safety evaluation indicators include.

(1) Dose-limiting toxicity (DLT)

(2) The incidence and severity of treatment-emergent adverse events (TEAE), etc.

(3) Other safety observation indicators include vital signs, 12-lead ECG and clinical laboratory test results, etc.

Table 4 summarizes the safety reporting periods and the patients included in each period. The post-infusion period will be the primary period for safety reporting.

Table 4 Safety reporting period

Period	Definition	Inclusion population
Pre-treatment	From the date of patient informed consent until the first lymphodepletion	Enrolled Patients
Lymphodepleting	From the first day of lymphodepletion	Patients receiving

chemotherapy period (this period is only for patients receiving lymphodepleting chemotherapy)	chemotherapy to the day before the infusion of CD33 CAR T cells	lymphodepleting chemotherapy
Post-infusion period	Within 30 days post CAR T cell infusion	Safety sets

6.1 Adverse Events

(Describe the events by frequency and incidence. The analysis plan needs to describe how the various adverse events/reactions are classified and summarized.)

Reporting of adverse events will be based on the Common Terminology Criteria for Adverse Events (CTCAE) version 5.0. CRS and GVHD grades will be based on the ASTCT[7] and EBMT consensus[8].

A summary table of AEs that started or worsened during the post-infusion period through the system organ class, preferred term, and severity (based on CTCAE 5.0 grade) will be provided. Patients with multiple grades of AE will be aggregated to the maximum grade recorded for that event. The frequency of grade 3 and 4 AEs will be aggregated separately.

- Adverse Events of Special Concern (AESC) include:

Cytokine release syndrome;

Central nervous system toxicity;

Graft-versus-host disease;

Infections (including viral infections, bacterial infections, etc.);

Hematologic toxicity.

In addition to investigator-reported adverse events, laboratory results analysis will be performed.

✧ **Pre-treatment:**

When using an enrolled set, adverse events that begin or worsen before lymphodepletion will be summarized separately as follows:

- Adverse events are classified by major organ category, preferred terminology, and highest grade.
- Severe adverse events are classified by major organ categories, the highest grade, and preferred terminology.

✧ **Lymphodepleting therapy Period:**

In addition, adverse events that begin or worsen during lymphodepleting therapy are summarized for all enrolled patients who receive lymphodepleting therapy.

The following tables will be generated:

- Adverse events, regardless of the relationship among drugs studied, are classified by major organ category, preferred terminology, and highest grade
- Severe adverse events, regardless of the relationship among drugs studied, classified by major organ category, preferred terminology, and highest grade
- For special adverse events, record the categories of major organs, the preferred terminology, and the highest grade

✧ **Post-infusion Period:**

The adverse events are evaluated between day 0 and day 30 post-infusion. Collecting adverse events will be stopped in patients who discontinue follow-up, are bridged to SCT, or receive other anti-leukemia therapy. A summary table of the following AE will be generated in the safety set:

- Adverse events, regardless of the relationship among drugs studied, are classified by major organ category, preferred terminology, and highest grade

- Severe adverse events, whether related to research drugs or not, are classified by major system organ category, preferred terminology, and highest grade

Death after infusion, classified by major system organ category and preferred terminology.

- Special adverse events, that occurred during the treatment progress, unrelated to study drugs (such as falling out of bed, trauma) classified by major organ category, preferred terminology, and highest grade

Serious adverse events need to be discussed through an independent review committee (IRC, independent review committee) on whether to discontinue research or not.

6.2 Laboratory Test

Analyses were according to the specific items (vital signs, physical examination, electrocardiogram examination, chest X-ray examination, etc.)

- For the laboratory tests covered by CTCAE, the biostatistics and reporting team of the study will grade the laboratory data accordingly.
- For laboratory tests with an undefined level of CTCAE, the results will be graded according to the normal range of the laboratory according to the low/normal/high classification.

The tables prepared by descriptive statistics will be generated: within 30 days after CAR T infusion.

According to CTCAE 5.0, hemocytopenia grades for absolute lymphocyte, absolute neutrophil, hemoglobin, platelet, or white blood cell counts will be obtained based on laboratory results. Patients with grade 3 or 4 hematopoietic cytopenia before lymphodepletion therapy and 30 days after infusion of CD33 CAR T cells are summarized. In patients with grade 3 or 4 hematopoietic cytopenia 30 days after infusion of CAR T cells,

the time to reach level 2 or below will be described in the table. If the patient fails to recover in the last laboratory evaluation, an examination will be performed at the next evaluation. The median recovery time at different time points (2nd month, 3rd month, etc.) will be summarized.

The following lists will be provided for the enrolled set:

- List the patients with abnormal laboratory results at CTCAE grade 3 or 4, as well as the corresponding CTCAE grade and classification according to the laboratory reference range.
- List the corresponding CTCAE grade and classification according to the laboratory reference range of all laboratory test results.

6.3 Cytokine Release Syndrome and Anti-Cytokine Release Syndrome Treatment

To explore the relationship between CRS and other indicators, because the limited research sample size is not enough to propose a scoring system, the goal of statistical analysis should be regarded as generating new scientific hypotheses and observing new trends.

Detailed information about CRS will be summarized in the disease response on the 30th day of the IRC assessment. The aggregated information includes the highest CRS level, the time of onset of CRS, the duration of CRS, concurrent infections, complications, and the use of anti-cytokine therapy, etc.

The relationship between the incidence of CRS and different donor sources of T cells will be evaluated with the chi-square test.

6.4 Graft-versus-host Disease and Treatment of Graft-versus-host Disease

To explore the relationship between GVHD produced by donor CAR T cell therapy and other indicators, because the limited research sample size is not enough to propose a scoring system, the goal of statistical analysis should be regarded as generating new

scientific hypotheses and observing new trends.

Detailed information about GVHD will be summarized in the disease response on the 30th day of the IRC assessment. The aggregated information included the highest GVHD level, the time of onset of GVHD, the duration of GVHD, concurrent infections, complications, and the use of anti-rejection therapy.

The relationship between the incidence of GVHD and different donor sources of T cells will be evaluated with the chi-square test.

6.5 Other Safety Data

Vital signs will be collected according to clinical needs.

7. BIOMARKER ANALYSIS

As a project standard, only biomarkers collected in the clinical database will be analyzed. Since the research is not sufficient to evaluate specific assumptions related to biomarkers, the objectives of this exploratory statistical analysis should be regarded as the generation of new scientific hypotheses. These hypotheses can be compared with the results found in the literature and can be verified by data from subsequent clinical trials.

Sometimes a decision may be made to discontinue sample collection for practical or strategic reasons (e.g., issues related to patient status and blood collection conditions), or to perform analysis of archival tumor samples/fresh tumor biopsies/fine-needle aspiration, or sample number or test-related issues. In such cases, the sample size may not be sufficient to perform rigorous data analysis and only the available data is listed and possibly summarized.

7.1 Biomarker Analysis Set

FAS set and safety set (SS) will be used for all biomarker analyses. The SS will be used to evaluate the association between biomarkers and safety data. Including: interleukin-6, interleukin-10, tumor necrosis factor-α, soluble CD25, ferritin, lactate dehydrogenase.

7.2 Data Processing of Serum Cytokine

The individual time curve of major inflammatory markers and cytokine parameters up to 1 month will be drawn.

8 EFFICACY ANALYSIS

8.1 Evaluation Indicators of Efficacy:

(1) Objective Remission Rate (ORR): Complete Remission Rate (CR), Partial Remission Rate (PR), and Duration of Remission (DOR)

(2) Progression-free Survival (PFS) and Overall Survival (OS)

8.2 Efficacy Assessment Standard

Table 5 Response Standard of NCCN[9,10]

Response Category	Definition
CR without minimal residual disease (CR_{MRD-})	If studied pretreatment, CR with negativity for a genetic marker by RT-qPCR, or CR with negativity by MFC.
Complete remission (CR)	Morphologic CR – transfusion independence<br>Bone marrow blasts <5%;<br>Absolute neutrophil count (ANC) ≥1.0×10^9/L (1,000/μL);<br>platelet count ≥100×10^9/L (100,000/μL)
CR with incomplete hematologic recovery (CRi)	All CR criteria except for residual neutropenia (<1.0×10^9/L [1,000/μL]) or thrombocytopenia (<100×10^9/L [100,000/μL])
Morphologic leukemia-free state (MLFS)	BM <5% blasts in an aspirate with spicules; at least 200 cells must be enumerated<br>No blasts with Auer rods or persistence of extramedullary disease

	If there is a question of residual leukemia, a BM aspirate/biopsy should be repeated in one week<br>A BM biopsy should be performed if spicules are absent from the aspirate sample.
Partial remission (PR)	Decrease of at least 50% in the percentage of blasts to 5% to 25% in the BM aspirate and the normalization of blood counts, as noted above
No response	Failure to qualify for CR/CRi/PR
Relapsed disease	Relapse following CR is defined as reappearance of leukemic blasts in the peripheral blood or the finding of more than 5% blasts in the BM, not attributable to another cause (eg, BM regeneration after consolidation therapy) or extramedullary relapse.

Minimal residual disease criteria by NCCN[10]

The role of MRD in prognosis and treatment is evolving. Participation in clinical trials is encouraged.

- MRD in AML refers to the presence of leukemic cells below the threshold of detection by conventional morphologic methods. MRD is a component of disease evaluation over the course of sequential therapy. If the patient is not treated in an academic center, there are commercially available tests available that can be used for MRD assessment. Patients whose disease achieved a CR by morphologic assessment alone can still harbor a large number of leukemic cells in the BM. The points discussed below are relevant to intensive approaches (induction chemotherapy) but have not been validated for other modalities of treatment.

- The most frequently employed methods for MRD assessment include real-time quantitative PCR (RQ-PCR) assays (ie, NPM1, CBFB::MYH11, RUNX1::RUNX1T1) and multicolor flow cytometry (MFC) assays specifically designed to detect abnormal MRD immunophenotypes. The threshold to define MRD^+ and MRD^- samples depends on the technique and subgroup of AML. NGS–based assays to detect mutated genes (targeted sequencing, 20–50 genes per panel) is not routinely used, as the sensitivity of PCR-based assays and flow cytometry is superior to what is achieved by conventional NGS. Mutations associated with clonal hematopoiesis of indeterminate potential (CHIP) and aging (ie, DNMT3A, TET2, potentially ASXL1) are also not considered reliable markers for MRD.
    - There are distinct differences between diagnostic threshold assessments and MRD assessments. If using flow cytometry to assess MRD, it is recommended that a specific MRD assay is utilized, but, most importantly, that it is interpreted by an experienced hematopathologist.
- Based on the techniques, the optimal sample for MRD assessment is either peripheral blood (NPM1 PCR-based techniques) or an early, dedicated pull of the BM aspirate (ie, other PCR, flow cytometry, NGS). The quality of the sample is of paramount importance to have reliable evaluation.
- Studies in both children and adults with AML have demonstrated the correlation between MRD and risks for relapse, as well as the prognostic significance of MRD measurements after initial induction therapy.
    - MRD positivity is not proof of relapse. However, a persistently positive MRD result after induction, which depends on the technique used and the study, is associated with an increased risk of relapse.
    - For patients with favorable-risk disease, if MRD is persistently positive after

induction and/or consolidation, consider a clinical trial or alternative therapies, including allogeneic HCT.

- Some evidence suggests MRD testing may be more prognostic than KIT mutation status in CBF-AML, but this determination depends on the method used to assess MRD and the trend of detectable MRD.
- After completion of therapy, "Molecular relapses" can predict hematologic relapses within a 3- to 6-month timeframe.

- Timing of MRD assessment:
  - Upon completion of initial induction.
  - Before allogeneic HCT.
  - Additional time points should be guided by the regimen used.

8.3 Clinical evidence of the establishment of CR/CR$_i$ and subsequent maintenance of CR/CRi without relapse

When CR or CRi is shown for the first time, a comprehensive response assessment is required, including assessment of peripheral blood, bone marrow, central nervous system symptoms, physical examination, and CSF assessment after lumbar puncture. If the patient does not undergo CR/CR$_i$ examination in the first month when clinical evidence of clinical remission is found through the peripheral blood and extramedullary disease assessment (physical examination and CNS) for the first time, bone marrow biopsy and CSF assessment through LP are also required to ensure that patients receive CR/CR$_i$ for the first time. Other bone marrow biopsies and CSF assessments through LP may also be recommended.

Therefore, after the initial acquisition of CR/CR$_i$, if the patient has no evidence of extramedullary disease (through physical examination and CNS assessment) and the circulating tumor cells in the peripheral blood are less than 5%, the clinical CR/CR$_i$ will be considered to have been maintained. To classify the best overall disease response as CR or

CR_i, within at least 1 month (30 days), the clinical evidence of lack of relapse should not be ensured with peripheral blood and extramedullary disease assessment (including physical examination and CNS symptom assessment) after initially reaching CR or CR_i. Note that if other evaluations are performed in the same evaluation time point (for example, bone marrow, CSF assessment through LP, CNS imaging, biopsies, etc.), remission status also needs to be displayed. The starting date was calculated as the initial evaluation date of CR or CR_i.

8.4 Date of Overall Response Assessment

The overall disease response date is the last date of all prescribed assessment time points (2 years) for all patients. Relapse or non-response can be assessed based on a partial assessment (for example, relapse is assessed from the blood only). The date of relapse or absence of response was calculated as the earliest date of all assessments showing relapse or absence of response.

8.5 Objective Remission Rate (ORR)

ORR is applicable within 3 months after CD33 CAR T cell infusion determined by IRC assessment. ORR is defined as the percentage of patients who have the best overall response to CR, CRi, and PR. Patients will be evaluated for safety and efficacy at the 15-day and 30-day following infusion of functionally enhanced CD33 CAR T cells. The best overall disease response is the best disease response recorded from the first CAR T-cell infusion to the starting of new anti-leukemia therapy (including SCT, but excluding purified $CD34^+$ stem cell infusion).

- The best overall response will be distributed in the following order:

1. CR; 2. CRi; 3. PR; 4. No response; 5. Unknown

The best overall disease response of the patient is always calculated according to the order of the disease response.

- To classify the best overall disease response as CR or CRi, after reaching the initial

CR or CRi, there must be obtained clinical evidence of no relapse through peripheral blood and extramedullary disease assessment (physical examination and CNS symptom assessment) after at least 1 month (30 days). Note that if there are other assessments of bone marrow and/or CSF in the same assessment time point, the remission status of these other assessments also needs to be displayed.

- If a patient has reached CR or CRi but has not been maintained for 30 days, the patient's best overall response will be considered as "no response". If the patient has reached CR or CRi without any follow-up response assessment, the patient's best overall response will be considered "CR or CRi".
- Calculate and report the proportion of patients with ORR.

ORR based on the data observed by the FAS. In addition, the respondent will be given a summary description of the event response (CR or CRi).

8.6 Duration of Remission (DOR)

The duration of remission is defined as the date from CR or CR_i response criteria first met to the date of relapse or death caused by potential cancer.

In DOR's main analysis (Method 1, in the following table), if the patient does not relapse or die from potential cancer before the data deadline, DOR will use the date of the last disease assessment as the censored date, which should be on or earlier than the earliest censored event date. The reasons for censor may be:

- loss of follow-up
- withdraw informed consent
- new anti-cancer therapy
- no longer provide a sufficiently effective assessment
- events that occur after at least two unplanned disease assessments

In addition, if any patient responds to CD33 CAR T-cell treatment but dies from a cause other than AML, death from a cause other than AML is considered a competitive risk event related to other events (relapse or death caused by AML). Sensitivity analysis will be used to analyze censored events due to other causes than AML.

Since SCT may be another effective anti-leukemic treatment option, the date of the SCT should be used as the censored date, rather than the date of the last disease assessment.

In the sensitivity analysis, the date of relapse or death after SCT (if attributed to potential cancer) is used to calculate DOR.

If the patient receives SCT after CR or CR_i, although data on individual disease response components (such as bone marrow) will not be collected, relapse or survival status after SCT will be recorded on the corresponding follow-up eCRF. Because the examination caused by SCT (Method 1) will lead to a down estimation of the relapse rate, it may not be appropriate for major analysis when a large number of patients choose to receive SCT.

Therefore, if at least one patient receives SCT during the remission phase of CAR T-cell infusion, the above sensitivity analysis will be performed. The planned DOR analysis is summarized in Table 5

Table 5 Analysis of Duration of Remission (DOR)

	Non-potential Cancer Deaths	SCT after Remission
Method 1	Competitive risk analysis	Censor time at SCT time
Method 2	Censor in the last disease assessment	Censor time at SCT time
Method 3	Competitive risk analysis	Ignore SCT
Method 4	Censor in the last disease assessment	Ignore SCT

DOR is evaluated only in patients with the best overall response to CR or CRi.

For Methods 1 and 3, CIF is used to estimate the probability of interested events when there is a competitive risk. These analyses are performed only if at least one patient has a competitive risk event.

For Methods 2 and 4, The KM method will be used to estimate the distribution function of DOR. If applicable, the median, min, and max values of DOR will be calculated.

For patients with recurrence, the following characteristics of the original relapse will be summarized:

- Original relapse site:

  Bone marrow or peripheral blood relapse;

  No extramedullary relapse;

  Unknown extramedullary state;

  Only extramedullary relapse;

  Relapse of extramedullary.

- CD33 status of initial bone marrow or peripheral blood relapse: determined by AML phenotype assessed by flow cytometry in bone marrow or peripheral blood;

  CD33 positive;

  CD33 low expression;

  CD33 negative;

  CD33 partial expression;

  Unknown;

If the status of CD33 is obtained from both bone marrow and peripheral blood, the bone marrow results will be used.

8.7 Progression-free survival (PFS)

Progression-free survival was defined as the earliest date from the first infusion of CD33 CAR T cells in patients who achieved objective remission to:

- Death for any cause after remission
- Relapse

If the patient has not relapsed and died for any reason before the cutoff date, PFS will use the date of the last disease assessment as the censoring date, which should be on or before the date of the earliest censoring event. The reason for the censor may be

- Lost of follow-up
- Withdraw consent
- The new anti-leukemia therapy
- No longer provide sufficient effective evaluation
- Events that occur after at least two unplanned disease assessments

In the main analysis of PFS, patients who are bridged to SCT during remission after infusion of CD33 CAR T cells would be censored at SCT. In addition, if SCT is performed in at least 1 patient after CD33 CAR T-cell infusion during remission, the date of recurrence or death after SCT (if attributed to underlying cancer) will be used in the calculation of DOR for PFS sensitivity analysis without censor SCT.

PFS will be evaluated in all patients, in FAS. PFS will be estimated using the Kaplan-Meier method. If applicable, the median PFS and 95% confidence intervals are calculated.

8.8 Overall survival (OS)

Overall survival is the time from the first infusion of CAR T cells to death from any cause.

Patients who have not died before the data cutoff date take their last contact date as the deletion date, which is defined as the last date they are known to be alive. If SCT is conducted, no censoring will be done. Therefore, patients who receive SCT should also be followed for survival.

All patients will be evaluated for OS in FAS. OS will be estimated using the Kaplan-Meier method. If applicable, the median of OS is calculated along with a 95% confidence interval.

9. Decision on Missing Conventional Disease Assessment

For some time points that were recorded at the end of the event (such as DOR), the censor or event classification may depend on the date of the last planned disease assessments.

The disease assessment schedule defined by the protocol is at baseline (1 day of time window), 30 days (7 days of time window), and each month thereafter (7 days of time window) until the earliest day when the following situation happens: disease progression or death, loss of follow-up, the start of another new anti-tumor treatment or completion all the evaluation time points.

10. PHARMACOKINETIC (PK) DATA ANALYSIS

Pharmacokinetic Evaluation Index: The pharmacokinetic evaluation index is the expansion and persistence of CAR T cells in the peripheral blood of the subjects.

The PAS set will be used for all PK summaries (tables and graphs). The FAS set will be used for PK data lists.

The absolute count and the percentage in lymphocytes of CAR T cells in peripheral blood (and CSF if applicable) are plotted and summarized by time point, as follows:

- The copy number of the DNA sequence of CAR vector measured by quantitative PCR
- CAR T cells and lymphocyte subgroups are measured by flow cytometry with different markers.

Results that have been reported but considered unreliable will be marked and excluded from the summary and PK parameter derivation.

- Analysis of individual patients, according to different dosage subgroups and different T cell donor sources.

11. References

1. Pan, J., *et al.* CD22 CAR T-cell therapy in refractory or relapsed B acute lymphoblastic leukemia. *Leukemia* **33**, 2854-2866 (2019).
2. Pan, J., *et al.* Frequent occurrence of CD19-negative relapse after CD19 CAR T and consolidation therapy in 14 TP53-mutated r/r B-ALL children. *Leukemia* **34**, 3382-3387 (2020).
3. Pan, J., *et al.* Sequential CD19-22 CAR T therapy induces sustained remission in children with r/r B-ALL. *Blood* **135**, 387-391 (2020).
4. Liu, F., *et al.* First-in-Human CLL1-CD33 Compound CAR T Cell Therapy Induces Complete Remission in Patients with Refractory Acute Myeloid Leukemia: Update on Phase 1 Clinical Trial. *Blood* **132**, 901-901 (2018).
5. Tambaro, F.P., *et al.* Autologous CD33-CAR-T cells for treatment of relapsed/refractory acute myelogenous leukemia. *Leukemia* **35**, 3282-3286 (2021).
6. Ataca Atilla, P., *et al.* Modulating TNFα activity allows transgenic IL15-Expressing CLL-1 CAR T cells to safely eliminate acute myeloid leukemia. *J Immunother Cancer* **8**(2020).
7. Lee, D.W., *et al.* ASTCT Consensus Grading for Cytokine Release Syndrome and Neurologic Toxicity Associated with Immune Effector Cells. *Biol Blood Marrow Transplant* **25**, 625-638 (2019).
8. Przepiorka, D., *et al.* 1994 Consensus Conference on Acute GVHD Grading. *Bone Marrow Transplant* **15**, 825-828 (1995).
9. Döhner, H., *et al.* Diagnosis and management of AML in adults: 2017 ELN recommendations from an international expert panel. *Blood* **129**, 424-447 (2017).
10. Pollyea, D.A., *et al.* NCCN Guidelines Insights: Acute Myeloid Leukemia, Version 2.2021. *J Natl Compr Canc Netw* **19**, 16-27 (2021).
