# Supplementary material for: C-JUN overexpressing CAR-T cells in acute myeloid leukemia: preclinical characterization and phase I trial
Source: Nat Commun. 2024 Jul 22;15:6155. doi: 10.1038/s41467-024-50485-9 (PMC11263573; doi:10.1038/s41467-024-50485-9)
Supplement: Supplementary file 3 — Reporting Summary [file 41467_2024_50485_MOESM3_ESM.pdf]

nature portfolio

Corresponding author(s): Xiaoming Feng

Last updated by author(s): May 27, 2024

Reporting Summary

Nature Portfolio wishes to improve the reproducibility of the work that we publish. This form provides structure for consistency and transparency in reporting. For further information on Nature Portfolio policies, see our Editorial Policies and the Editorial Policy Checklist.

Statistics

For all statistical analyses, confirm that the following items are present in the figure legend, table legend, main text, or Methods section.

n/a	Confirmed	
☐	☒	The exact sample size (*n*) for each experimental group/condition, given as a discrete number and unit of measurement
☐	☒	A statement on whether measurements were taken from distinct samples or whether the same sample was measured repeatedly
☐	☒	The statistical test(s) used AND whether they are one- or two-sided<br>*Only common tests should be described solely by name; describe more complex techniques in the Methods section.*
☒	☐	A description of all covariates tested
☐	☒	A description of any assumptions or corrections, such as tests of normality and adjustment for multiple comparisons
☐	☒	A full description of the statistical parameters including central tendency (e.g. means) or other basic estimates (e.g. regression coefficient) AND variation (e.g. standard deviation) or associated estimates of uncertainty (e.g. confidence intervals)
☐	☒	For null hypothesis testing, the test statistic (e.g. *F*, *t*, *r*) with confidence intervals, effect sizes, degrees of freedom and *P* value noted<br>*Give P values as exact values whenever suitable.*
☒	☐	For Bayesian analysis, information on the choice of priors and Markov chain Monte Carlo settings
☒	☐	For hierarchical and complex designs, identification of the appropriate level for tests and full reporting of outcomes
☒	☐	Estimates of effect sizes (e.g. Cohen's *d*, Pearson's *r*), indicating how they were calculated

Our web collection on statistics for biologists contains articles on many of the points above.

Software and code

Policy information about availability of computer code

Data collection	LSR Fortessa (BD Biosciences), Canto II (both BD Bioscience), FACS Aria II (BD Biosciences) for flow cytometry data acquisition;<br>Living Image ver 4.2 (PerkinElmer) for IVIS Spectrum bioluminescent imaging acquisition;<br>The RNA sequencing library was prepared and sequenced on Illumina NovaSeq 6000 platform.
Data analysis	FlowJo software ver 10.8.0 for analysis of flow cytometry results;<br>GraphPad Prism 9 for data analysis and visualization;<br>GSEA software (Broad Institute) for GSEA;<br>TBtools software for heatmaps;<br>Living Image ver 4.2 (PerkinElmer) for visualization and calculation of total luminescence.

For manuscripts utilizing custom algorithms or software that are central to the research but not yet described in published literature, software must be made available to editors and reviewers. We strongly encourage code deposition in a community repository (e.g. GitHub). See the Nature Portfolio guidelines for submitting code & software for further information.

Data

Policy information about availability of data

All manuscripts must include a data availability statement. This statement should provide the following information, where applicable:

- Accession codes, unique identifiers, or web links for publicly available datasets
- A description of any restrictions on data availability
- For clinical datasets or third party data, please ensure that the statement adheres to our policy

All requests for raw and analyzed data will be subject to review by the corresponding authors to determine whether there are any intellectual property or confidentiality considerations. Patient-related data not included in the manuscript may be restricted due to patient confidentiality. We have received IRB approval and have obtained consent to report data on these 4 patients. The clinical trial study protocol is available in the Supplementary Information file. The sequencing data have been deposited in the Genome Sequence Archive database under accession code HRA007312 and HRA004747 at https://ngdc.cncb.ac.cn/gsa-human/submit/hra/submit. All remaining data can be found in the Article, Supplementary Information, and Source Data files, and from the corresponding author upon reasonable request. Source data are provided with this paper.

Research involving human participants, their data, or biological material

Policy information about studies with human participants or human data. See also policy information about sex, gender (identity/presentation), and sexual orientation and race, ethnicity and racism.

Reporting on sex and gender	Sex: female or male. Consents have been obtained. Gender was not considered in the study design.
Reporting on race, ethnicity, or other socially relevant groupings	We do not use or collect data about ethnicity.
Population characteristics	In the preclinical studies, the samples were obtained from healthy donors and patients with diagnosed AML and ALL.<br>In the clinical trial, to be eligible to participate in this study, an individual must meet all of the following criteria: 1. Candidates with relapse or refractory CD33+ acute myeloid leukemia, who have progressed after treatment with all standard therapies or are intolerant of standard therapy, have limited prognosis with currently available therapies and had no available curative treatment options (such as SCT or chemotherapy); 2. Male or female, aged 1-70 years; 3. No serious allergic constitution; 4. Eastern Cooperative Oncology Group (ECOG) performance status score 0 to 2; 5. Have a life expectancy of at least 60 days based on the investigator's judgment; 6. CD33 positive in bone marrow or cerebrospinal fluid (CSF) by flow cytometry, or CD33 positive in tumor tissues by immunohistochemistry; (CD33 positive criteria: Flow cytometry: Positive: > 80% of tumor cells expressed CD33 and the MFI of CD33 is the same as that in normal myeloid cells; Dim: > 80% of tumor cells expressed CD33, but the MFI of CD33 is lower than that in normal myeloid cells as least as 1log; Partial positive: 20%-80% of tumor cells expressed CD33 and the MFI of CD33 is the same as that in normal myeloid cells. tumor tissue immunohistochemistry: Positive > 30% tumor cells expressed CD33); 7. Provide a signed informed consent before any screening procedure; subjects who voluntarily participate in the study should have the ability to understand and sign the informed consent form and be willing to follow the study visit schedule and relevant study procedure, as specified in the protocol. Candidates aged 19-70 years need to be sufficiently conscious and able to sign the treatment consent form and voluntary consent form. Pediatric patients aged 1-7 years could be recruited after signing an informed consent form by a legal surrogate (Guardian); pediatric patients aged 8-18 years need to be sufficiently conscious and voluntarily sign an informed consent form, and their legal surrogates (guardians) were also required to sign a written informed consent form.
Recruitment	Fresh PBMCs from healthy human blood donors were provided by TianJin Blood Center, and the recruitments of healthy human blood donors were approved by the Ethics Committee of the Institute of Hematology and Blood Diseases Hospital. All the participants signed the Informed Consent Form.<br>In the clinical trial, participants were recruited by the investigators pursuant to IRB approved methods to reduce the possibility of biased recruiting at Beijing GoBroad Boren Hospital.
Ethics oversight	All experiments involving human samples were approved by the Ethics Committee of the Institute of Hematology and Blood Diseases Hospital (approval number: NKRDP2021009-EC-2).<br>The Phase I, open-label study (NCT04835519) was conducted at Beijing GoBroad Boren Hospital. The study protocol was approved by the Institutional Review Board (IRB) of Beijing GoBroad Boren Hospital. Written informed consent was obtained from the patients or their legal guardians before enrollment (ethics approval number: 20210331-TY-001K). All clinical investigations were conducted in accordance with the Declaration of Helsinki principles.

Note that full information on the approval of the study protocol must also be provided in the manuscript.

Field-specific reporting

Please select the one below that is the best fit for your research. If you are not sure, read the appropriate sections before making your selection.

☒ Life sciences ☐ Behavioural & social sciences ☐ Ecological, evolutionary & environmental sciences

For a reference copy of the document with all sections, see nature.com/documents/nr-reporting-summary-flat.pdf

Life sciences study design

All studies must disclose on these points even when the disclosure is negative.

Sample size	In the preclinical experiments of our study, no statistical method was used to predetermine sample size. But all major in vitro experiments were performed with at least three independent biological replicates. Size of human samples were based on the sample availability and the consistency of measurable differences between groups.
Data exclusions	No data were excluded
Replication	All experimental replicates are described in the figure legends. Not applicable for clinical data.
Randomization	For all in vivo tumor models, mice were randomized to ensure equivalent tumor burden in groups. Randomization for in vitro experiments is not applicable.
Blinding	Blinding was not applicable to the clinical study due to the open-label, single arm nature of this study. No blinding protocols were used to generate the pre-clinical data.

Reporting for specific materials, systems and methods

We require information from authors about some types of materials, experimental systems and methods used in many studies. Here, indicate whether each material, system or method listed is relevant to your study. If you are not sure if a list item applies to your research, read the appropriate section before selecting a response.

Materials & experimental systems

n/a	Involved in the study	
☐	☒	Antibodies
☐	☒	Eukaryotic cell lines
☒	☐	Palaeontology and archaeology
☐	☒	Animals and other organisms
☐	☒	Clinical data
☒	☐	Dual use research of concern
☒	☐	Plants

Methods

n/a	Involved in the study	
☒	☐	ChIP-seq
☐	☒	Flow cytometry
☒	☐	MRI-based neuroimaging

Antibodies

Antibodies used

anti-human CD3 (Biolegend Cat#: 317335 Clone OKT3),
anti-human CD4 (Biolegend Cat#: 357410 Clone A161A1),
anti-human CD8 (Biolegend Cat#: 344711 Clone SK1),
anti-mouse CD45 (Biolegend Cat#: 103116 Clone 30-F11),
anti-human CD34 (Biolegend Cat#: 343519 Clone 581),
anti-human CD22 (Biolegend Cat#: 363505 Clone S-HCL-1),
anti-human CD38 (Biolegend Cat#: 356608 Clone HB-7),
anti-human CLL1 (Biolegend Cat#: 353605 Clone 50C1),
anti-human CD123 (Biolegend Cat#: 306005 Clone 6H6),
anti-human CD117(c-kit) (Biolegend Cat#: 375203 Clone S18022G),
anti-human CD366(Tim-3) (Biolegend Cat#: 345025 Clone F38-2F2),
anti-human CD233(LAG-3) (Biolegend Cat#: 369317 Clone 11C3C65),
anti-human CD62L (Biolegend Cat#: 304821 Clone DREG-56),
anti-human CD45RA (Biolegend Cat#: 304127 Clone HI100),
anti-human CD279(PD-1) (Biolegend Cat#: 329905 Clone EH12.2H7),
anti-human CD25 (Biolegend Cat#: 302610 Clone BC96),
anti-human CD86 (Biolegend Cat#: 374205 Clone BU 63),
anti-human CD69 (Biolegend Cat#: 310911 Clone FN50),
anti-human CD28 (Biolegend Cat#: 302925 Clone CD28.2),
anti-human CD137 (4-1BB) (Biolegend Cat#: 309809 Clone 4B4-1),
anti-human/mouse Granzyme B Recombinant Antibody (Biolegend Cat#: 372203 Clone QA16A02),
anti-human IFN-γ (Biolegend Cat#: 506527 Clone B27),
anti-human IL-2 (Biolegend Cat#: 500306 Clone MQ1-17H12),
anti-human IL-21 (Biolegend Cat#: 513003 Clone 3A3-N2),
anti-human CD155 (Biolegend Cat#: 337609 Clone SKIL4),
anti-ERK1/2 phospho(Thr202/Thr204) (Biolegend Cat#: 369516 Clone 6B8B69).

Validation

All antibodies used in this study are commercially avaliable. Antibodies have been validated and routinely tested as described in the

manufacturer's product information (Biolegend, eBioscience, CST and TransGen Biotech).

Eukaryotic cell lines

Policy information about cell lines and Sex and Gender in Research

Cell line source(s)	The human cell lines were derived from the Cell Resource Center of our institute in the study. These cell lines were Nalm6, Raji, SUP-B15, U937, THP-1, HL-60, 293T cells.
Authentication	STR Fingerprinting was conducted once per year.
Mycoplasma contamination	All cell lines were free of mycoplasma contamination
Commonly misidentified lines (See ICLAC register)	No commonly misidentified cell lines were used in the study.

Animals and other research organisms

Policy information about studies involving animals; ARRIVE guidelines recommended for reporting animal research, and Sex and Gender in Research

Laboratory animals	Six- to ten week female NOD.Cg-PrkdcscidIl2rgtm1Wjl/SzJ (NSG) mice were obtained from Beijing Biocytogen and no other animals were used in the study.
Wild animals	No wild animals were used in the study.
Reporting on sex	female
Field-collected samples	No field-collected samples were used in the study.
Ethics oversight	All animal experiments performed in this study were in strict accordance with institutional guidelines and were approved by the Institutional Animal Care and Use Committees of State Key Laboratory of Experimental Hematology (SKLEH). The mice were housed in a specific pathogen-free (SPF) environment.

Note that full information on the approval of the study protocol must also be provided in the manuscript.

Clinical data

Policy information about clinical studies

All manuscripts should comply with the ICMJEguidelines for publication of clinical research and a completedCONSORT checklist must be included with all submissions.

Clinical trial registration	ClinicalTrials.gov NCT04835519
Study protocol	The protocol is included in the Supplemental Materials
Data collection	All data was collected between April 12, 2021 and July 15, 2023 at Beijing GoBroad Boren Hospital. Clinical data was collected in eijing GoBroad Boren Hospital.
Outcomes	The primary objectives of this trial was to assess the safety and feasibility of C-JUN CD33 CAR T cells. Moreover, there were several secondary objectives: (1) To evaluate the anti-tumor activity of C-JUN CD33 CAR T cells in subjects with r/r AML. (2) To characterize the pharmacokinetic (PK) profile of C-JUN CD33 CAR T cells in r/r AML patients.

Flow Cytometry

Plots

Confirm that:

- [x] The axis labels state the marker and fluorochrome used (e.g. CD4-FITC).
- [x] The axis scales are clearly visible. Include numbers along axes only for bottom left plot of group (a 'group' is an analysis of identical markers).
- [x] All plots are contour plots with outliers or pseudocolor plots.
- [x] A numerical value for number of cells or percentage (with statistics) is provided.

Methodology

Sample preparation	Human T cells were derived from healthy individuals' peripheral blood and were obtained using the EasySepTM Human T cell Enrichment Kit (Stem Cell Technologies) in accordance with the description of the reagent. For intracellular cytokine staining was conducted by the FoxP3 Staining Buffer Set (eBioscience) in accordance with description of the reagent. For flow cytometry, cells at 4°C for 30 minutes in darkness.

Instrument	LSR Fortessa or CantoII (BD Bioscience); FACS Aria II, BD Biosciences
Software	FlowJo
Cell population abundance	Cell sorting was employed in this study. The post-sorted population purity was tested by flow cytometry and over 95% purity.
Gating strategy	Tipically, cells were first gated by FSC and SSC plots, then single cells were selected by FSC-H vs FSC-A. Finally, prior to the gate of interest, live cells were selected as defined by Live/Dead stain negativity. Detailed gating strategies were provided in supplementary information.

☒ Tick this box to confirm that a figure exemplifying the gating strategy is provided in the Supplementary Information.
